# Nanoscale Analysis beyond Imaging by Atomic Force Microscopy: Molecular Perspectives on Oncology and Neurodegeneration

**DOI:** 10.1002/smsc.202500351

**Published:** 2025-10-12

**Authors:** Carlos Marcuello, KeeSiang Lim, Giacomo Nisini, Vadim S. Pokrovsky, João Conde, Francesco Simone Ruggeri

**Affiliations:** ^1^ Laboratorio de Microscopias Avanzadas (LMA) Universidad de Zaragoza Zaragoza 50018 Spain; ^2^ Biofisika Institute CSIC‐Universidad del País Vasco/Euskal Herriko Unibertsitatea (UPV/EHU) 48940 Leioa Spain; ^3^ WPI‐Nano Life Science Institute Kanazawa University Ishikawa 920‐1192 Japan; ^4^ Physical Chemistry and Soft Matter Wageningen University Stippeneng 4 6708 WE Wageningen Netherlands; ^5^ Organic Chemistry Wageningen University Stippeneng 4 6708 WE Wageningen Netherlands; ^6^ Patrice Lumumba People's Friendship University 117198 Moscow Russia; ^7^ Comprehensive Health Research Centre (CHRC) Faculdade de Ciências Médicas (FCM) Universidade Nova de Lisboa 1099‐085 Lisboa Portugal

**Keywords:** amyloidogenic proteins, atomic force microscopy, biomolecular processes, cancer diseases, force spectroscopy, nano‐chemical analysis, neurodegeneration

## Abstract

Nanobiomedicine promises to revolutionize life quality and expectancy of patients with cognitive impairment and cancer malignancies, via unraveling key molecular processes related to their onset useful as biomarkers of disease to develop and improve the efficacy of therapies. However, it is still a challenge understanding and identifying these molecular mechanisms as biomarkers of disease, because of their high‐level of polymorphism and nanoscale dimensions. Here, it provides a review work linking the potential and capabilities of atomic force microscopy (AFM) technologies in unraveling beyond imaging the common and hidden properties of transient and nanosized molecular processes in cancer and neurodegeneration. This study highlights the most prominent operational modes of AFM to achieve morphological, mechanical, and chemical characterization of the molecular processes leading to these diseases. Finally, it outlines the advantages of AFM compared with other techniques to guide newcomers and stakeholders toward potential future avenues opened by AFM methods in nanobiomedicine.

## Graphical abstract

Cancer and neurodegeneration share interconnected nanosized molecular pathways. Novel nanobiomedicine approaches promise targeted biomarkers and therapies. Atomic force microscopy (AFM) is invaluable to characterize nanosized biomolecular processes beyond imaging. AFM methods allow multimodal nano‐imaging, mechanical and chemical analysis. Multiparameter nano‐information is key to define biomarkers of disease. Nanobiomarkers pave the way to monitor disease onset, progression, interventions.

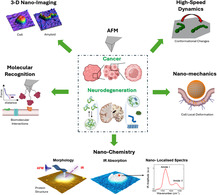

## 1 Introduction

Improving quality of life and prolonging life expectancy are currently key challenges for society.^[^ ^1^, ^2^ ^]^ We need to reduce burden of disease and health inequalities of citizens, resulting from an unhealthy lifestyle and environment, to improve lifespan and quality of elderly population.^[^ ^3^ ^]^ One of the main cornerstones to overcome these challenges is the development of novel targeted therapies to fight debilitating and fatal human diseases, such as cancer and neurodegeneration.^[^ ^4^, ^5^, ^6^, ^7^, ^8^ ^]^

According to recently published epidemiological data, the global occurrence of neurodegenerative disorders and cancer together was of more than 30 million new cases per year,^[^ ^1^, ^2^ ^]^ for a total of ≈160 and ≈250 million disability‐adjusted life years, respectively. These diseases together lead to almost 20 million deaths per year. The World Health Organization (WHO) estimates increased incidence of ≈50% for both cancer and neurodegenerative disorders, respectively, over the next three decades. The care expenses associated to these diseases will cost over 20 trillion dollars over the year 2050.^[^ ^9^, ^10^ ^]^

The multifaceted mechanisms involved in cancer and neurodegenerative diseases cover a wide array of factors, including internal molecular and genetic factors combined with extrinsic nutritional, lifestyle, and environmental factors.^[^ ^11^, ^12^, ^13^, ^14^ ^]^ Gene expression and fusion is crucial for disease progression and can act as a potential prognostic indicator.^[^ ^15^, ^16^ ^]^ Furthermore, polymorphisms and a large number of protein variants and interactions,^[^ ^5^, ^17^ ^]^ combined with genetics, nutrition, and lifestyle, can drive complex relationships and molecular mechanisms of cancer and neurodegeneration onset and progression, which are yet to be elucidated.^[^ ^13^, ^14^ ^]^

Within genetic and molecular factors, the structural state of biomolecules at the nanoscale is key for human diseases onset and progression, with significant impact dictated by the folding, structure, and supramolecular assembly state of protein. External environmental factors can trigger pathological transitions between different protein states in our organism, which are in turn highly regulated by chaperones keeping protein in a physiological state.^[^ ^18^ ^]^ For example, redox conditions can promote the activation of phosphorylation signaling cascades that control nucleosome and proteasome formation for the breakdown of DNA^[^ ^19^ ^]^ and proteins,^[^ ^20^ ^]^ respectively. Then, aberrant chaperones behavior fails to regulate protein thermodynamic stability and conformations, which trigger protein misfolding and aggregation effects deregulating cell and organism homeostasis.^[^ ^16^, ^21^ ^]^ Moreover, metal ions disbalance can significantly affect tertiary and quaternary structure of proteins, leading to misfolding and altering their biological function.^[^ ^22^ ^]^ These misfolded proteins may have the ability to surpass the components of proteostasis that are not recognizable by proteosomes,^[^ ^21^ ^]^ thereby favoring disease progression.

Cancer and neurodegenerative diseases further share highly interconnected intrinsic molecular pathways, such as mitochondrial dysfunction,^[^ ^23^, ^24^ ^]^ oxidative stress,^[^ ^24^, ^25^ ^]^ protein degradation, misfolding, and aggregation.^[^ ^16^, ^26^ ^]^ For instance, reactive oxygen species (ROS) production is implicated in mitochondrial DNA mutations leading to cancer,^[^ ^27^ ^]^ fatty acid (FA) oxidation, and subsequent formation of lipoperoxidation products driving brain toxicity.^[^ ^25^, ^28^ ^]^ The pathophysiology of both diseases is also closely linked by the deregulation of the glycogen synthase kinase 3β (GSK3β) and sirtuin pathways, which can induce both malignancies.^[^ ^29^ ^]^ Some efforts have been devoted to this field, such as the design of artificial transcription factors that have shown promising capabilities in controlling tunable gene expression and restoring normal cell regulation;^[^ ^30^ ^]^ however, precise knowledge of their mechanisms of action is still elusive. Misfolding, alteration of condensed phase and aggregation of more than 50 proteins is associated with cancer^[^ ^26^ ^]^ and neurodegeneration,^[^ ^24^ ^]^ including p53 (cancer),^[^ ^5^ ^]^ Aβ/tau (Alzheimer's disease),^[^ ^31^, ^32^ ^]^ α‐synuclein (Parkinson's disease),^[^ ^33^ ^]^ prion proteins^[^ ^34^ ^]^ (Creutzfeldt‐Jakob disease), fused in sarcoma (FUS, Amyotrophic lateral sclerosis, cancer),^[^ ^35^, ^36^ ^]^ and huntingtin (Huntington's disease).^[^ ^37^ ^]^ The nature of the aggregates related to cytotoxicity,^[^ ^38^ ^]^ and the mechanisms by which they contribute to disease are still unclear. However, the abundancy of these proteins in human biofluids has been correlated with disease onset and progression.^[^ ^32^, ^39^, ^40^, ^41^ ^]^ Moreover, antibody‐based pharmacotherapies have targeted neurodegeneration by depleting toxic amyloid aggregates from brains of patients but yet with modest results.^[^ ^42^, ^43^ ^]^ Notwithstanding these efforts, effective disease‐modifying treatments nor cures are yet available for neurodegeneration or cancer. The above‐described illustrative examples evidence the importance of identifying molecular processes suitable as biomarkers of disease onset and progression, as well as useful to tailor therapies.

To unravel external and internal factors contributing to the onset of cancer and dementia, large international efforts have led to development of a wide array of analytical methods for characterizing the physicochemical properties of proteins, cells, and tissues. Many bulk techniques are now available to study average chemical and structural properties of biomolecular systems and protein misfolding and self‐assembly processes, including small‐angle X‐ray scattering (SAXS), nuclear magnetic resonance (NMR) spectroscopy, dynamic light scattering (DLS), thioflavin T (ThT) fluorescence assays, infrared (IR) and Raman spectroscopy, and circular dichroism (CD).^[^ ^44^, ^45^ ^]^ Among these methods, kinetic measurements by ThT and chemical kinetics theory are powerful tools that can be applied to analyze the chain of events characterizing protein aggregation at the microscopic level.^[^ ^16^ ^]^ At the larger biological scale of cellular properties, flow cytometry is used to distinguish the size and internal complexity differences of cells, by detecting scattered light at different angles or using fluorescently tagged antibodies to visualize cell surface and cytoplasmic antigens. The concentration of specific analytes within cells can be detected using enzyme‐linked immunosorbent assays (ELISA). The kinetics of complex biological interactions and their stoichiometry can be quantified by surface plasmon resonance (SPR)^[^ ^46^ ^]^ or fluorescence cross‐correlation spectroscopy (FCCS).^[^ ^47^ ^]^ Moreover, cellular and biomolecular interactions can be addressed via bioluminescence resonance energy transfer (BRET) measurements.^[^ ^48^ ^]^ These tools share a common capability for measuring cellular and biomolecular adhesion properties. The mechanical properties of the bulk tissue can be investigated by ultrasonic testing,^[^ ^49^ ^]^ multifrequency magnetic resonance elastography (MRE)^[^ ^50^ ^]^ or micro‐indentation using a Berkovich tip.^[^ ^51^ ^]^ These techniques can be combined with deep learning‐based cell‐tracking methods to predict modeling for experimental design and assist analysis of large datasets.^[^ ^52^ ^]^ However, the main limitations of the above‐described bulk techniques are their lack of sensitivity to unravel transient phenomena, single molecule events, and hidden short‐lived cellular states.

Single‐molecule techniques have emerged as promising alternatives to overcome the drawbacks of bulk approaches, which are unable to characterize the heterogeneity of complex biomolecular pathways and cellular heterogeneous states. Optical tweezers (OT) can trap single molecules and cells by using a highly focused laser beam.^[^ ^53^ ^]^ For instance, OT monitor the force interactions displayed at cell interfaces during cellular migration to explore the membrane forces that guide the uptake of particles into cell bodies.^[^ ^54^ ^]^ Alternatively, magnetic tweezers (MT) can manipulate objects by the precise movement of paramagnetic beads through externally applied magnetic fields. MT offers excellent results in stretching biomolecules^[^ ^55^ ^]^ and unravelling cellular nanomechanics.^[^ ^56^ ^]^ The outstanding noise‐threshold force detection limits of OT and MT are below 15 pN, but these tools do not allow the imaging and visualization of the tested samples.

Atomic force microscopy (AFM) is a multiparametric technique capable of nano‐imaging, determining a variety of physicochemical properties and investigating the interplay between the structure; chemistry; adhesion; and mechanics of cellular systems, biomolecules, and their exerted biology functions.^[^ ^57^ ^]^ AFM reconstructs the 3D morphology of a sample on a flat surface by monitoring the distance‐dependent interaction forces between a sharp probe and the sample, with Ångström sensitivity on the vertical axis and lateral spatial resolution in the range of ≈0.5–10 nm, achieving pure single‐molecule sensitivity.^[^ ^44^, ^45^ ^]^ High‐speed AFM (HS‐AFM) enables video‐rate image acquisition to monitor biomolecular structure and dynamics in motion with a resolution well below 1 s per frame in in liquid physiological conditions.^[^ ^58^ ^]^ The nanomechanical properties of the sample can also be assessed using the AFM cantilever as a force sensor and the tip as a nano‐indenter, which causes elastic sample deformations after applying a load force.^[^ ^59^ ^]^ The use of chemically functionalized tips allow AFM‐force spectroscopy (AFM‐FS) measuring the specific intra‐ and intermolecular adhesion forces when the tip interacts with the most external sample surface;^[^ ^60^ ^]^ the unbinding events are then measured through force‐distance curves when the tip is retracted and the formed biology complex dissociates. Molecular recognition imaging studies are conducted by AFM‐FS when the setup operates in the low‐force regime and the unspecific tip‐sample forces become negligible.^[^ ^61^ ^]^ The development of fast AFM‐FS has further allowed simultaneous and quantitative mapping of morphology and nanomechanical properties of cells and tissues,^[^ ^62^ ^]^ as well as to study the changes in stiffness of biomolecules and proteins, for instance, as a function of their structural polymorphism^[^ ^63^ ^]^ and variations in sequence.^[^ ^64^ ^]^ AFM can be further combined with the chemical recognition power of vibrational spectroscopy, such as Raman^[^ ^65^ ^]^ and infrared (IR) spectroscopy.^[^ ^66^, ^67^ ^]^ The combination of the high spatial resolution of AFM with the chemical recognition power of IR has led to the development of infrared nanospectroscopy (AFM‐IR) to enable simultaneous multimodal nanoscale imaging of the morphology and mechanical and chemical properties. AFM‐IR analysis can be performed both in liquid^[^ ^68^ ^]^ and air environments,^[^ ^64^, ^69^ ^]^ down to single‐molecule detection of protein chemical–structural properties.^[^ ^66^, ^70^ ^]^

In contrast to other nanoscale techniques, such as electron microscopy (EM), AFM allows 3D nanoscale analysis with no need for class‐averaging and beyond imaging and multimodal analysis of the physicochemical properties of biomolecules, cells, and tissues. As further advantage, AFM can be carried out in any media, such as air, vacuum, and liquid mimicking physiological conditions (buffers and temperature) occurring in living organisms. An essential advantage of AFM is its ability to measure living cells in a liquid environment at an appropriate temperature (37 °C) to provide physiologically relevant conditions.^[^ ^71^, ^72^ ^]^ AFM requires only minimal quantity of sample (μL); it does not require labelling (label free) or the addition of external contrast agents compared with other techniques, such as scanning electron microscopy (SEM). Coating the bio‐sourced sample surface with sputtered contrast agents could drive unwanted artifacts, such as potential sample damage and modifications of its topography.^[^ ^73^ ^]^

AFM‐based technologies have been then successfully applied to address the molecular action mechanisms of several human pathologies or to act as sensitive biosensor for detection of stage of disease, in the case of pathologies caused by: viruses and bacteria;^[^ ^74^ ^]^ maligns cancers, such as breast,^[^ ^75^ ^]^ cervical,^[^ ^76^ ^]^ and ovarian;^[^ ^77^ ^]^ and protein misfolding diseases, such as the neurodegenerative Parkinson's disease (PD),^[^ ^33^ ^]^ Alzheimer's disease (AD),^[^ ^32^ ^]^ amyotrophic lateral sclerosis (ALS),^[^ ^78^ ^]^ Huntington's disease (HD),^[^ ^37^ ^]^ ataxias,^[^ ^79^ ^]^ and Creutzfeldt–Jakob prion disease.^[^ ^34^ ^]^

In cancer research, AFM offer a noninvasive platform for the nano‐analysis of cells and tissues, such providing unprecedented details on cell membrane shape and stiffness.^[^ ^75^, ^80^ ^]^ Imaging can be used to differentiate between cancer and normal cells and evaluate the interactions between neighboring cells.^[^ ^71^ ^]^ AFM‐FS can measure nanomechanical differences between tumor nodes and neighboring tissues with exquisite precision, allowing to identify single cancer cells.^[^ ^81^, ^82^ ^]^ AFM‐FS has been further used to image and measure the stiffness of intracellular compartments and protein structural components within cells, such as microtubules.^[^ ^83^, ^84^ ^]^ AFM‐IR can further provide correlative information on surface topography and chemical signature of cells, allowing the identification of specific biomarkers that may be crucial in distinguishing between different types of cancer cells.^[^ ^85^ ^]^ These capabilities make AFM‐methods a strong contender in the field for detection, diagnosis; having the potential to monitor the effectiveness of treatments^[^ ^86^ ^]^ by observing the changes in mechanical and chemical properties before and after treatment. This could lead to personalized and effective treatment strategies to improve the prognosis of cancer patients.^[^ ^71^ ^]^

In neurodegeneration, the above described features have been used for studying the structure, mechanical and chemical properties of misfolded protein, and their amyloid assemblies.^[^ ^44^, ^45^, ^87^ ^]^ Nano‐imaging can detect and discriminate different amyloid species at different aggregation time occurring in vitro, for instance, to study of α‐synuclein,^[^ ^88^, ^89^, ^90^ ^]^ prion,^[^ ^34^, ^91^, ^92^ ^]^ huntingtin,^[^ ^37^, ^64^, ^93^, ^94^ ^]^ Aβ,^[^ ^95^, ^96^ ^]^ FUS,^[^ ^35^, ^78^ ^]^ as well as in vivo human biofluids^[^ ^40^, ^41^, ^97^ ^]^ and tissues.^[^ ^32^, ^33^, ^98^ ^]^ AFM empowers performing ultrastructural studies of protein aggregates and understand how their morphology changes with the variation in internal factors in living organisms or with extrinsic factors in vitro.^[^ ^99^ ^]^ AFM‐FS and nanomechanical imaging methods accurately probe the mechanical properties of protein aggregates and help understand how they influence amyloid toxicity.^[^ ^63^, ^89^, ^100^ ^]^ Moreover, AFM‐IR can be exploited to collect crucial information on the secondary and quaternary structures of protein and their self‐assembly state, to study how the chemical signature of aggregates is related to their toxicity.^[^ ^35^, ^64^, ^70^ ^]^

In this review work, we highlight in detail these achievements and further potential of AFM‐based methods to perform 3D nanoscale characterization beyond imaging of key molecular mechanisms involved in the onset and progression of cancer and neurodegenerative diseases. In the frame of the study of these disorders, we first illustrate the basic principles of sub‐nanometer revolved AFM imaging and video‐rate HS‐AFM to study the 3D morphology and heterogeneity of single molecules and cells. We then delve into force spectroscopy‐based molecular recognition and mechanical nanoscale analysis to unravel adhesion and stiffness properties. We finally highlight the latest developments of AFM‐IR to empower nanochemical analysis of submolecular and subcellular physicochemical structural states. This review work not only highlights most recent breakthroughs but also summarizes common molecular mechanisms between these apparently unrelated pathologies. These approaches and information may lead to the development of next‐generation nanobiomedicine approaches and identification of novel therapeutic targets.

## 2 Molecular Mechanisms of Cancer and Neurodegeneration

### 2.1 Cancer Diseases

Cancer, a disease characterized by the uncontrolled growth and spread of pathological cells, is a global health concern.^[^ ^1^, ^101^ ^]^ Cancer arises from the transformation of normal cells into tumor cells in a multistage process that generally progresses from precancerous lesions to malignant tumors. These changes are the result of the interaction between the individual person genetic factors and external agents,^[^ ^11^ ^]^ such as toxins, ultraviolet, and ionizing radiation.^[^ ^102^ ^]^

**Figure 1:**
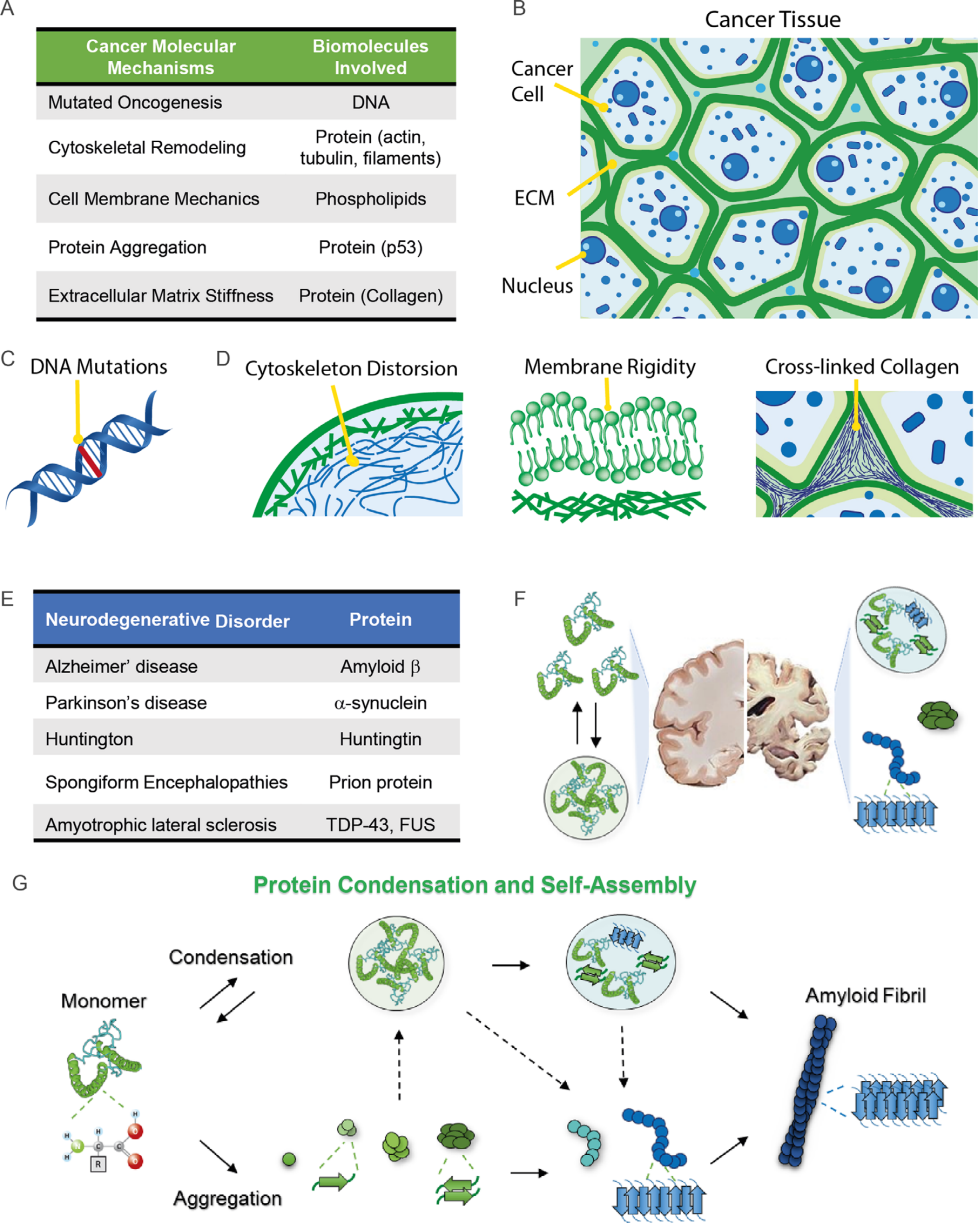
Common biomolecular processes in cancer and neurodegeneration diseases. A) Molecular mechanism associated to typically involved biomolecules. B) Schematics of human tissue and the C,D) associated molecular mechanisms of cancer: Mutations in certain oncogenes and gene‐suppressors are primary causes of malignant transformation of normal cells; dysregulated growth of cancer cells affect proteins of extracellular matrix, which undergo significant remodeling, including changes in the composition and organization of collagen fibers, leading to increased cross‐linking and enhanced matrix stiffness; mutations and environmental pressure can cause cytoskeleton structures to change, resulting in enhanced contractility; and changes in membrane mechanical properties due to acidic environments and compressive force promote cancer cells invasion. E) Misfolding and aggregation of protein and peptides are associated with the onset of devasting neurodegenerative disorders. F) Protein misfolding causes the structural change of protein structure and liquid‐liquid phase separated state to form gel‐like condensates and amyloid aggregates in the brain of patients. G) General depiction of protein condensation and its recently unraveled link with amyloid formation.

Several key genetic and molecular mechanisms are related to the onset and progression of cancer, such as mutated oncogenesis and/or genes suppression,^[^ ^15^, ^27^, ^103^ ^]^ extracellular matrix (ECM),^[^ ^71^, ^104^ ^]^ cytoskeletal remodeling,^[^ ^105^ ^]^ and altered membrane rigidity^[^ ^104^, ^106^ ^]^ (**Figure** **1**A). These molecular mechanisms drive proliferation and formation of cancer tissue (Figure 1B).

DNA alterations affect cell growth, division, differentiation, and their survival (Figure 1C).^[^ ^15^, ^27^, ^107^ ^]^ These genetic mutations can be inherited or acquired, leading to the dysfunction of critical regulatory pathways. Key processes involved include activation of oncogenes, loss of tumor suppressor genes, and defects in DNA repair mechanisms.^[^ ^108^ ^]^ Environmental factors, such as viruses also lead to damage the host DNA in cells, which can trigger uncontrolled cell growth and division.^[^ ^12^ ^]^ Additionally, cancer cells can evade immune detection and create an environment that supports their growth, while inhibiting normal cellular functions.^[^ ^104^, ^109^ ^]^ Cancer cells often exhibit altered metabolism, which allows them to survive and proliferate under conditions that are unfavorable to normal cells.^[^ ^109^, ^110^ ^]^ The ability of cancer cells to metastasize and invade other organs and tissues is a critical step in the progression to a lethal disease.^[^ ^104^, ^111^ ^]^

Alterations in the design and organization of the cytoskeleton and membrane cellular structures, as well as the extracellular matrix, manifest as altered mechanical properties of tumors (Figure 1D).^[^ ^81^, ^112^ ^]^ The stiffness of certain types of cancer, such as solid tumors located within soft tissues, enables their detection by physical palpation. Despite increased density of cancer tissue, most scientific literature indicates that cancer cells are generally softer than their normal counterparts, more deformable, and more motile.^[^ ^113^ ^]^ These changes have been described for example for breast,^[^ ^75^ ^]^ bladder,^[^ ^80^ ^]^ and many other cancer cells.^[^ ^71^, ^114^ ^]^ Increased motility enhances the invasive properties of cells, allowing them to penetrate neighboring tissues and vessels, ultimately facilitating metastasis.^[^ ^112^ ^]^

A key mechanisms responsible for the mechanical changes in cancer cells is cytoskeletal remodelling.^[^ ^105^ ^]^ The cytoskeleton, a network of protein‐based polymers (actin, tubulin, and intermediate filaments), maintains the shape of the cell, secures intracellular organelles at specific positions, enables cell movement, and plays a crucial role in cell division. In cancer cells, mutations and environmental pressure can cause structural distortions of the cytoskeleton; resulting in enhanced contractility, generating tension on the cell membrane, and further pulling on the surrounding ECM. Membrane tension is another key molecular mechanism that leads to mechanical changes in cancer cells.^[^ ^112^ ^]^ Healthy cells maintain a delicate balance of forces on their cell membranes to preserve their shape and volume. However, cancer cells often exhibit altered chemical composition and membrane tension. Cancer cell membranes exhibit increased phosphatidylethanolamine (PE) and negatively charged phosphatidylserine (PS) lipids, lower cholesterol and long‐chain ceramide levels, and higher polyunsaturated FAs. Membrane tension is primarily due to changes in cytoskeletal structure and ion channel activity, which can promote cell migration and invasion and contribute to cancer progression.^[^ ^112^ ^]^ The uncontrolled proliferation of tumor cells leads to their dense accumulation within a defined space. Cytoskeletal and membrane tension are, in turn, transmitted through the ECM. Consequently, the ECM undergoes significant remodeling, including changes in the composition and organization of collagen fibers, leading to increased cross‐linking and enhanced matrix stiffness. Prolonged compressive stress can induce collagen fiber deposition and parallel alignment.^[^ ^115^ ^]^ Also, tumors are often characterized by disturbed “in–out” fluid balance in the interstitial space and increased interstitial fluid pressure (IFP).^[^ ^104^, ^110^ ^]^ This is primarily due to the abnormal growth of blood vessels within the tumor, leading to the leakage of fluid into the interstitial space and poor lymphatic drainage. The resulting fluid buildup contributes to mechanical pressure within the tumor, creating a vicious cycle that further enhances tumor stiffness and aggressiveness.

Understanding at the nanoscale the altered morphological,^[^ ^116^ ^]^ mechanical and chemical properties^[^ ^117^, ^118^ ^]^ of individual cells and tumor nodes driven by cytoskeletal remodeling,^[^ ^105^ ^]^ membrane tension alterations,^[^ ^112^ ^]^ and extracellular matrix impairment^[^ ^104^, ^119^ ^]^ can provide valuable insights into disease progression, diagnostics, and treatment. For instance, altered stiffness and increased motility of cancer cells could be used as biomarkers for cancer diagnosis,^[^ ^106^, ^120^, ^121^, ^122^ ^]^ as well as critical molecular information for understanding the pathogenesis of cancer and developing new therapeutic strategies.

### 2.2 Neurodegenerative Disorders

Neurodegenerative diseases belong to a class of diseases called amyloidosis or misfolding disorders, which involve the misfolding of specific proteins or peptides, leading to the subsequent formation of self‐assemblies termed amyloids (Figure 1E).^[^ ^16^, ^123^ ^]^ The majority (>90%) and most common disease, such as AD and PD, are predominantly sporadic;^[^ ^16^ ^]^ however, there exist hereditary forms of neurodegenerative disorders, such as HD,^[^ ^124^ ^]^ and infectious diseases, such as spongiform encephalopathies.^[^ ^16^ ^]^ The majority of these pathologies share common characteristics, such as cognitive deficits, behavioral disturbances, and motor impairment.

These similarities in symptom manifestation are also associated with a common etiology at the microscopic and nanoscopic level. It is widely accepted that neurodegenerative diseases are associated with protein misfolding and aggregation.^[^ ^16^, ^24^ ^]^ This idea was proposed several decades ago after the identification of protein aggregates in brain biopsies of patients with AD (Figure 1F).^[^ ^125^ ^]^ Despite obvious differences in the sequences of different proteins which undergo aggregation, the intermediate and final species produced during amyloid formation share common universal features (Figure 1G).^[^ ^123^ ^]^ The final insoluble products of aggregation, namely amyloid fibrils, are all characterized by a universal cross‐β‐sheet structure, independent of the initial monomer. The cross β‐sheet structure consists of β‐strand repeating subunits, forming a hydrogen‐bonded β‐sheet, perpendicularly oriented with respect to the fibril axis. All amyloid fibrils show nanometers scale diameter and lengths of up to several micrometers.^[^ ^44^ ^]^

Originally, in the framework of the so‐called amyloid hypothesis, it was postulated that neurodegenerative diseases are caused by the formation and accumulation of amyloids in the brain.^[^ ^125^ ^]^ However, several consistent pathological observations suggested that amyloid fibrils may not be toxic agents and led to the reconsideration of this hypothesis.^[^ ^125^ ^]^ These findings include the following: Amyloid fibrils derived from different proteins were found in the post‐mortem tissues of people not affected by neurodegeneration; amyloid load did not always correlate with disease onset or severity; the absence of a correlation between the extent of fibril formation and neurodegeneration in AD animal models;^[^ ^126^, ^127^ ^]^ and therapeutic clearance of amyloid plaques in humans did not yet fully result in reversal or improvement in clinical symptoms of AD.^[^ ^125^ ^]^ These evidences boosted the research for alternatives to the amyloid hypothesis and led to the formulation of the oligomer hypothesis, which states that oligomers, rather than fibrils, may be the primary cause of toxicity and cell death in AD^[^ ^32^ ^]^ and PD.^[^ ^33^, ^123^ ^]^

Oligomers are intermediate, low‐molecular‐weight aggregates on‐pathway and off‐pathway of amyloid fibrils formation (Figure 1G). Structurally, they exhibit a mixture of size, shape, and secondary structure conformation^[^ ^6^ ^]^ often containing intermolecular cross‐β‐sheet conformations. However, compared with amyloid fibrils, oligomeric intermediates often exhibit poorer binding to the amyloid‐specific dyes thioflavin T/S (ThT/S) and Congo red,^[^ ^128^ ^]^ thus indicating they do not fully acquire the cross‐β structure characteristic of amyloid fibrils. Similar to amyloid fibrils, oligomers display high polymorphism and heterogeneous dynamic and structural properties. This high degree of polymorphism has shown that it is unlikely that a specific molecule or antibody would recognize all types of oligomers formed by one protein, thus hampering the development of successful pharmacological approaches.^[^ ^123^ ^]^ Furthermore, the intrinsic heterogeneity and metastability of oligomers make difficult to isolate and investigate their structural, functional, and toxic properties of a single oligomeric species.

Notwithstanding this challenge, several studies have shown that the different physical‐chemical properties of oligomers can lead to different mechanisms of toxicity, including membrane permeabilization, calcium dysregulation, mitochondrial damage, inflammation, and oxidative stress.^[^ ^6^, ^16^, ^17^ ^]^ Hydrophobic oligomers were observed to interact more readily with lipid membranes, while size and shape of the aggregates may determine their affinity of binding to receptors. Further successful attempts demonstrated specific mechanisms of toxicity exerted by recently discovered elongated oligomeric species, termed protofilaments.^[^ ^32^, ^38^, ^89^, ^98^ ^]^ These protofilament species lack a mature cross‐sheet structure and exhibit a high degree of flexibility, allowing the formation of an annular pore‐like structure.^[^ ^98^ ^]^ In vitro studies have shown that mutations of the amyloid‐β (Aβ) peptide and α‐synuclein (αS) protein, which are linked to the onset of AD and PD respectively, may promote the formation of amyloid pores with toroidal shape on the surface of the cell membrane causing its permeabilization leading to cellular toxicity.^[^ ^123^ ^]^

In the last years, the process of protein aggregation has been placed in the broader context of protein liquid–liquid phase separation (LLPS, Figure 1G).^[^ ^129^ ^]^ Many proteins associated with the onset of neurodegenerative diseases undergo LLPS, such as αS,^[^ ^130^, ^131^ ^]^ Tau,^[^ ^132^ ^]^ Huntingtin,^[^ ^129^ ^]^ FUS,^[^ ^133^ ^]^ and TAR DNA‐binding protein 43 (TDP‐43).^[^ ^134^ ^]^ The protein‐rich phase resulting from phase separation consists of liquid‐like condensates, also known as biomolecular condensates. These condensate act as membraneless organelles in our cells, which include nucleoli,^[^ ^135^ ^]^ stress granules,^[^ ^136^ ^]^ and Cajal bodies,^[^ ^137^ ^]^ fulfilling crucial tasks for cellular functioning, such as gene expression, response and adaptation to stress, and ribosome biogenesis.^[^ ^135^, ^138^ ^]^ However, the attractive interactions needed to initiate phase separation, and the increased protein concentration within condensates, may also prepare the ground for the formation of irreversible fibrillar aggregates that are related to protein malfunction and the onset of neurodegeneration.^[^ ^139^ ^]^ Indeed, it has been shown that liquid‐like condensates can act as intermediates in amyloid formation via a liquid‐to‐solid transition.^[^ ^140^ ^]^ For example, the low‐complexity domain of hnRNPA1 and FUS, proteins involved in ALS, showed that condensates promote fibril formation via their interface.^[^ ^78^, ^130^ ^]^ The liquid‐to‐solid transition is thus not spatially uniform within a condensate, and the interface between the dense and dilute phases of condensates can promote amyloid formation by inducing conformational changes and affect fibril nucleation.^[^ ^141^ ^]^ Thus, protein condensation may act as key molecular mechanisms regulating health protein function versus protein malfunction in disease and neurodegeneration.

Overall, the current state‐of‐the‐art suggests that the heterogeneous molecular interplay at the nanoscale between phase separation, protein misfolding and amyloid formation is key for the onset and progression of neurodegenerative disorders. Thus, single‐molecule techniques, such as AFM, have the potential to unravel these molecular mechanisms and provide fruitful avenues for the development of targeted therapeutics against neurodegeneration.^[^ ^8^ ^]^

## 3 3D Morphology Nano‐Imaging by AFM

AFM has emerged as one of the most powerful and versatile single‐molecule techniques because of the possibility of acquiring 3D morphology maps of biological specimens, with Ångström resolution on the vertical axis (<0.1 nm, Z), and at best with ≈0.5–1 nm lateral resolution on the XY axis,^[^ ^45^, ^142^ ^]^ in both air and their native liquid environment.^[^ ^45^, ^143^, ^144^ ^]^ The limited resolution on XY direction is inherent to AFM convolution effect, where the tip apex size and shape dictate the lateral resolution on the scanned feature.^[^ ^45^ ^]^ AFM nano‐imaging can detect key hallmarks linked to proliferation and inhibition of cancer processes in tissue, as well as unravel the processes of amyloid formation in vitro and ex vivo. This knowledge could significantly aid in a better understanding of the molecular mechanisms underlying these diseases, opening promising gates in the development of fast and point‐of‐care diagnostic methodologies.

### 3.1 Principles

**Figure 2:**
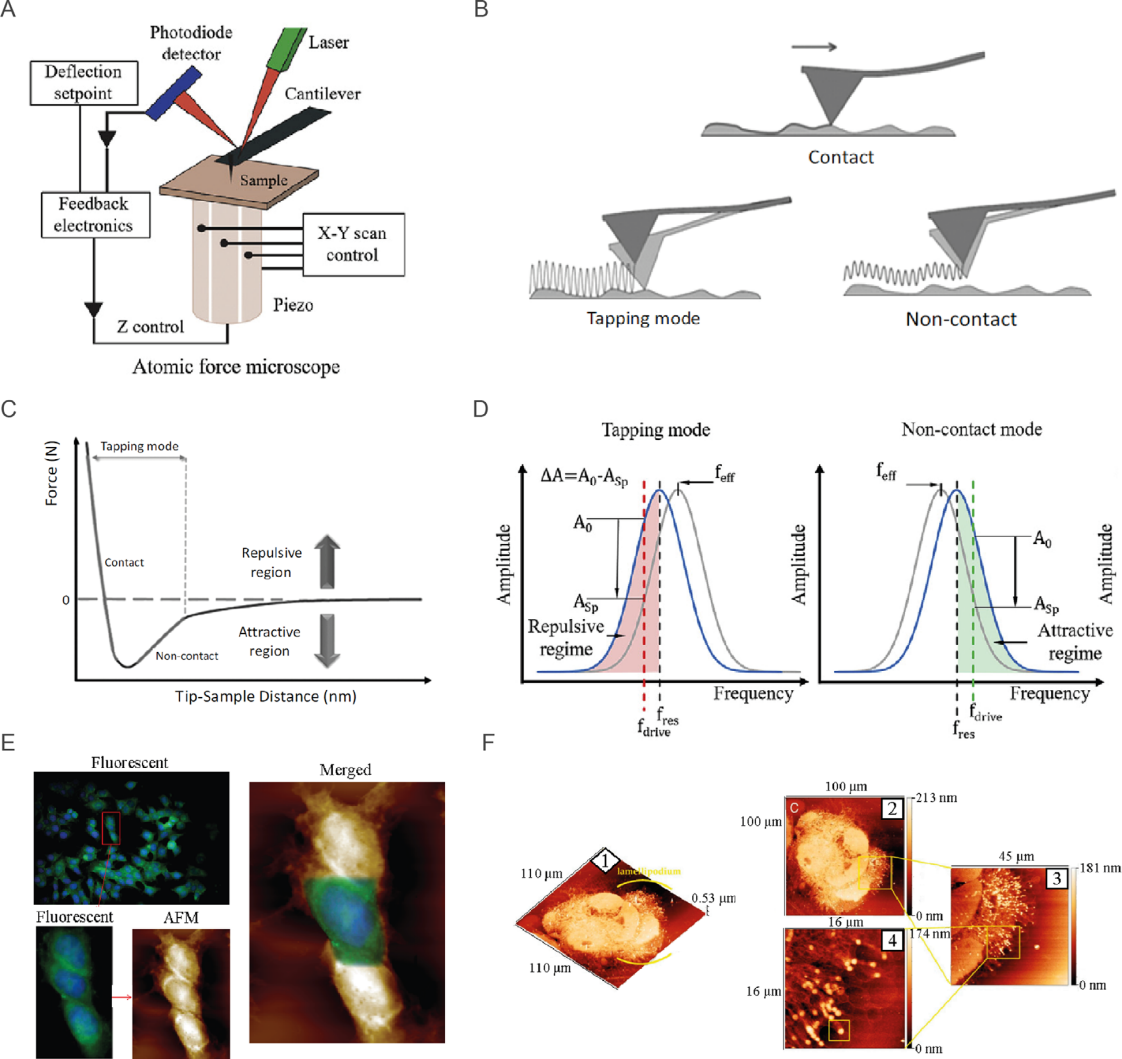
AFM working principles and example of application to cancer cells. A) Simplified representation of an atomic force microscope (adapted and printed from ref. [144]). B) Schematic of AFM scanning modes and C) related AFM tip‐sample potential energy representation. D) Schematic representation of a change in the effective frequency *f*_eff_ of oscillation in tapping mode and noncontact mode. E) MCF‐7 breast cancer cells labeled with anti‐vasodilator‐stimulated phosphoprotein (VASP) antibody and 4′‐6‐diamino‐2‐phenylindole (DAPI) dye and visualized by fluorescent microscopy combined with AFM (green and blue colors, respectively). Printed with permission from,^[^ ^176^ ^]^ Copyright 2019, Wiley. F) Morphology of HCC38 human breast cell lamellipodium after exposure to X‐rays at different scan sizes. 1) 110 μm × 110 μm (3D‐map); 2) 110 μm × 110 μm; 3) 45 μm × 45 μm; and 4) 16 μm × 16 μm. Printed with permission from,^[^ ^210^ ^]^ Copyright 2021, Multidisciplinary Digital Publishing Institute.

AFM setup was developed using several structural components (**Figure** **2**A).^[^ ^45^, ^144^ ^]^ The AFM probe is placed at the end of a cantilever, which can be considered a flexible oscillator with multiple resonance frequencies.^[^ ^145^ ^]^ The probes commonly used in AFM are made of silicon or silicon nitride, which can be pyramidal, cylindrical, or conical in shape, with an apical radius typically between 1 and 50 nm.^[^ ^45^ ^]^ Spherical colloidal probes with larger radius up to above 10 microns are commonly used to avoid damaging the structural integrity of cells.^[^ ^146^, ^147^ ^]^ The cantilever is mounted in a holder to perform measurements. Cantilevers can have different geometries, the most common of which are triangular or rectangular, typically 10–200 μm in length.^[^ ^145^ ^]^ Normally, the holder is customized depending on whether the measurements are performed in liquid or air environments. When the experiments are conducted in liquid media, if the system does not use a specific liquid cell, the holder can be sealed to the substrate by an accessory *O*‐ring system to prevent buffer release which can damage the piezoelectric scanner tube. AFM measurements with an environmental chamber can precisely control the relative humidity or temperature by coupling a Peltier heating stage accessory.^[^ ^148^ ^]^

In the simplest operational mode of AFM, termed contact mode, the force (*F*) sensed by the probe at the end of the flexible cantilever follows the linear Hookean relationship Equation (1)^[^ ^45^ ^]^

(1) F(x)=−kΔx

where *k* (N/m) is the spring constant of the AFM cantilever and Δ*x* (m) is the cantilever deflection that typically ranges from 0.1 to 1000 nm. This enables the routine measurement of forces in the order of 5 ≈ 10^−12^ N.^[^ ^149^ ^]^ Cantilever deflection is typically monitored by a laser beam reflected off its external side onto a position‐sensitive photodetector (PSD).^[^ ^144^, ^145^ ^]^ PSD is divided into four quadrants, and the relative laser position is recorded with the cantilever bending caused by the AFM tip‐sample interaction, providing information about the surface topography.

To acquire 3D morphology data, the sample needs to be deposited and immobilized on an, possibly atomically, flat substrate. Commonly used substrates include mica, mica‐APTES, highly ordered pyrolytic graphite (HOPG), gold, silicon, zinc sulphide (ZnS), zinc selenide (ZnSe), and glass among others.^[^ ^45^ ^]^ In this context, different types of functionalization allow differential adsorption and avoid non‐desirable dragging during scanning.^[^ ^150^ ^]^ Electrostatic and covalent strategies have been developed to achieve this purpose.^[^ ^151^ ^]^ Yet, the process of deposition of biological samples on the surface of choice is widely based on hands deposition. Thus, it is highly dependent on the manual skills of the operator. A generic methodology of sample preparation conceptually consists of the following key steps:^[^ ^152^, ^153^ ^]^ i) deposition of the sample onto a solid surface; ii) rinsing with water or ideally with the working buffer to detach weakly attached molecules, for measurements in liquid environment; iii) and drying the sample for measurements in air. These steps together take an amount of time ranging from a few seconds to several minutes. The time and concentration of deposition of the sample via physio‐ or chemisorption must be carefully evaluated, since during the deposition time several artifacts can often occur and frustrates quantitative AFM studies of biological samples.^[^ ^152^ ^]^ Indeed, the accurate control of the quantity of biomolecules in the sample deposited on a surface is challenging. Moreover, during the time of adsorption, the biomolecules could orient, align and self‐assemble following the crystalline order of the surface.^[^ ^152^ ^]^ Moreover, the use of substrates with a fixed charge state do not guarantee that the full content of the sample in solution will be attached on the surface after rinsing and/or the drying steps. In the case of cells, growing them directly on biocompatible of functionalized surfaces, such as glass, ZnSe, ZnS, CaF, and BaF, is a suitable approach. However, for biomolecules and biofluids, electrostatic repulsion and competitive binding between different molecules in solution can reduce the fraction of the adsorbed molecule on the surface. While these deposition artifacts can be greatly reduced by expert users, they are a primary cause of misinterpretation of the content and biophysical properties of heterogeneous biological systems by unexperienced users and newcomers. To overcome the limitations of manual preparation of AFM samples, recently a lab‐on‐a‐chip microfluidic spray deposition has been proposed.^[^ ^152^, ^153^ ^]^ This microfluidic spray handles valuable biological sample depositing them in a single step on a surface in the form of droplets of subpicoliter volume evaporating in a time scale of milliseconds. The single‐step deposition and the millisecond drying allow preserving the full heterogeneity and molecular conformation of the biological sample under investigation for nano‐analysis by conventional AFM imaging, as well as by more advanced modes further described in this review.

After the sample is deposited on, a surface is then mounted on a piezoelectric scanner made of a ceramic material which is able to move the sample with respect to the probe in the vertical (Z) and horizontal (X, Y) directions.^[^ ^45^, ^144^, ^145^ ^]^ The piezoelectric elements expand or contract in response to externally applied voltages, thereby enabling precise sample positioning in the three‐axis direction. Open‐loop piezoelectric scanners assume that the displacement is linearly proportional to the voltage; thus, they are subjected to hysteresis, creep, and cross‐coupling that negatively affect their performance.^[^ ^154^ ^]^ The eventual nonlinearity effects and skew errors generated by open‐loop scanners can be eliminated by applying image‐field corrections^[^ ^155^ ^]^ or by using more advanced close‐loop scanners, which can be coupled with feedforward vibrational control sensors to reduce noise.^[^ ^156^ ^]^ Positive position feedback (PPF) control can be also implemented to compensate cross‐coupling scan‐induced vibration.^[^ ^157^ ^]^ Further efforts have been devoted to developing nano‐positioning stages to control the close‐loop velocity to improve performance compared with PFF systems.^[^ ^158^ ^]^ Electronic controllers are finally responsible for processing photodetector signals by integrated lock‐in‐based alternating current detection. Proportional‐integral‐derivative (PID) controllers manage fast feedback to acquisition signals using a closed‐loop system; iterative learning algorithms have been developed to tune PID parameters and optimize AFM‐acquired signals.^[^ ^159^ ^]^

AFM can be operated in different imaging modes divided into static and dynamic ones.^[^ ^45^, ^142^ ^]^ Figure 2B shows some of the most commonly used imaging modes, with their respective cantilever amplitude modulation motions according to the excitation frequency.^[^ ^45^ ^]^ First, in static contact mode, the AFM tip is in close contact with the surface of the sample. The feedback is settled by the cantilever deflection, as measured by the 4‐quadrants photodiode; in this mode of operation, strong repulsive and frictional forces dominate (Figure 2C). Thus, AFM probes with a low spring constant are recommended to avoid damaging sample integrity.^[^ ^160^ ^]^ However, the contact mode is not ideal for soft biological matter measurements, because the lateral shear forces exerted by the cantilever tip can distort the biological structure of the assessed samples. To overcome the limitations of contact‐mode AFM, alternative operational dynamic modes have been developed, which can be divided into: amplitude modulation (AM) and frequency modulation (FM) modes, Force‐Volume, and PeakForce Tapping.^[^ ^142^ ^]^

Both AM and FM can be operated in the single‐frequency (SF) and multifrequency (MF) modes.^[^ ^142^, ^143^ ^]^ AFM dynamic modes are based on intermittent contact between the tip and the sample because of the constant oscillation of the cantilever. In AM (or FM), the cantilever amplitude (or frequency) is the controlled feedback and changes in the amplitude (frequency) oscillation detect variations in the Z direction owing to the topography contribution of the sample surface.^[^ ^45^ ^]^ For this purpose, the cantilever is driven close to its resonance frequency (*f*_0_),which is given by the lever spring constant and mass (*m*) (Equation 2).^[^ ^145^ ^]^

(2) $$f_{0}=\sqrt{\frac{k}{m}}$$

*f*_0_ shifts when the AFM probe approaches the sample surface because of changes in the effective spring constant (*k*_eff_), which are induced by the sensed tip‐sample forces, leading to a damped harmonic oscillator (Equation 3).^[^ ^145^ ^]^

(3) $$k_{\text{eff}}=k_{0}-\frac{\partial F}{\partial z}$$

In AM, the feedback loop controls the oscillation amplitude, and two main regimes can be differentiated (Figure 2D): i) tapping, when the oscillation is close‐below to its resonance frequency, and the tip‐sample interaction is a dynamic interplay among attractive (e.g., Van der Waals or electrostatic interactions) and repulsive forces (Pauli repulsion),^[^ ^45^ ^]^ and ii) noncontact, when the oscillation is close‐above to its resonance frequency, and the tip mainly senses long‐range van der Waals interaction forces.^[^ ^45^ ^]^ Moreover, multifrequency AFM (MF‐AFM) was also developed to map multiple sample properties during the imaging acquisition.^[^ ^59^, ^161^ ^]^ MF‐AFM operates at higher Eigenmode excitation frequencies, and the leverage of the nonlinear dynamics of cantilever oscillations with nonlinear sample force potentials allows the simultaneous detection of different sample properties, such as viscoelasticity.^[^ ^162^ ^]^ Optimization of the cantilever architecture enables the tuning of higher‐order Eigenmodes to increase the spatial‐temporal image resolution.^[^ ^163^ ^]^

Force volume allows imaging of the morphology of soft matter using as control feedback the force of interaction between the tip and the sample in contact mode, allowing to collect complementary mechanical information along with morphology by recording cantilever deflection during approach‐retraction cycles.^[^ ^164^ ^]^ Force values (*F*) are measured at each coordinate of the surface (x,y), as a function of the distance from the sample (z), to retrieve its morphology.^[^ ^57^ ^]^ Therefore, a force‐volume map contains force values per each spatial coordinate (x,y,z). When the tip is withdrawn from a pixel, it is modulated by a triangular function to move to the next point; thus, lateral forces are minimized avoiding damage of soft samples. However, time of acquisition are significantly slower than AFM dynamic modes, such as AM and FM. To overcome the drawbacks of force‐volume imaging, PeakForce tapping has been developed.^[^ ^63^, ^165^, ^166^ ^]^ Peakforce tapping retrieves the morphology of the sample by intermittently contacting the tip at frequencies well below the cantilever resonances, usually between 0.25 and 2.0 kHz, and using as feedback a peak force setpoint.^[^ ^167^ ^]^ The peak force setpoint is the maximum value of the force imposed to the sample and can be as small as 10 pN, thus preventing sample damage.^[^ ^168^ ^]^ Differently from force‐volume, leveraging a sinusoidal modulation of the z‐position of the tip, rather than a triangular one, further avoids resonances at the inflection point and allows to acquire morphological and mechanical information as in conventional dynamic modes.^[^ ^63^, ^69^ ^]^

Overall, MF‐AFM, AM(FM)‐AFM, and Peakforce modulations are the most common and suitable options for imaging soft‐matter samples. Although not independent, two different types of resolution should be distinguished: lateral and vertical.^[^ ^45^ ^]^ The vertical resolution is limited by both noise from the detection system and thermal fluctuations of the cantilever and is normally on the order of 10 pm.^[^ ^45^ ^]^ Lateral resolution is affected by three main factors: the instrumental resolution, defined by the ratio between the size and number of pixels of the image, the precision and sensitivity of the piezoelectric scanner movement along the XY direction, and the radius of the scanning tip. Additionally, for lateral resolution, tip convolution should be considered in AFM imaging measurements. This broadening effect is based on the finite volume of the AFM tip which may obscure boundary location points.^[^ ^45^ ^]^ Deconvolution correction methods are employed to reconstruct the gathered AFM images and obtain detailed morphological structures of the scanned features after the algorithm iteration steps according to the tip geometry.^[^ ^169^ ^]^ Furthermore, these smart algorithm frameworks can be extended to identify the relationships between the tip geometry and surface roughness, providing robust models to quantify other types of tip‐sample surface effects.^[^ ^170^ ^]^ Thus, the selection of the AFM probe directly depends on the purpose of the measurements and nature of the scanned sample. Different tip shapes and sizes are commercially available. For ultrasensitive studies, it is possible to control the growth of a single carbon nanotube on the AFM tip apex, achieving lateral resolutions below 1 nm.^[^ ^171^ ^]^ For the morphological study of cancer cells and amyloidogenic proteins in physiological conditions soft AFM probes (*k* = 0.2–10 N/m) with sharp tips (nominal radius < 5 nm) and dynamic imaging modes are recommended.

### 3.2 Nano‐Imaging of Biomolecular Mechanisms in Cancer

High‐resolution nanoscopic imaging of cancer cells and tissues allows for a detailed examination of their morphology, including dynamic modifications of the cellular membrane.^[^ ^116^ ^]^ For example, surface topography roughness has been demonstrated to act as an efficient marker to track cancer cell response.^[^ ^172^ ^]^ The overexpression of scaffold/matrix‐associated region 1‐binding protein (SMAR1) resulted in a loss of cellular roughness, and the migration dynamics of tumor spheroids in 3D cell cultures was proved dependent on cellular roughness.^[^ ^116^ ^]^ SMAR1 protein triggers the activation of the tumor suppressor p53 through serine‐15 residue phosphorylation,^[^ ^173^ ^]^ driving the activation of the p21 downstream effector.^[^ ^174^ ^]^ Consequently control SMAR1‐siRNA treated cells in mouse melanoma (B16F1), human embryonic kidney 293, and human breast cancer (MCF‐7) cell lines showed decreasing cellular roughness, and different migration dynamics.^[^ ^116^ ^]^ All these findings highlight the strong impact of membrane architecture on cancer cell behavior. Accurate topography characterization is not only relevant for cancer cells but also for those bodies to which they interact. For example, anisotropic topographic microgratings can modulate the migration pattern of breast cancer cells via microtubule reorganization.^[^ ^175^ ^]^

AFM can also be coupled with other techniques, such as fluorescence microscopy, to gather complementary information about cancer cell signaling events, enabling a deeper understanding of cellular processes. For example, MCF‐7 breast cancer cells have been immunolabeled with 4′‐6‐diamino‐2‐phenylindole (DAPI, blue) to visualize the cell nucleus, and with antivasodilator‐stimulated phosphoprotein (VASP, green) antibodies to visualize lamellipodia formation (Figure 2E).^[^ ^176^, ^177^ ^]^ This dual labeling is crucial since cancer typically leads to deregulation of the cell cycle and abnormalities in DNA accumulation, promoting the lamellipodia formation by actin polymerization, which is closely related to cell migration and invasion in cancer. The high‐spatial resolution of AFM have allowed visualizing the 3D structure of the actin networks within the lamellipodium, in the case of cells subjected to procarcinogenic soft X‐ray irradiation (Figure 2F).^[^ ^178^ ^]^ The versatility of combining AFM nanoscale imaging combined with fluorescence microscopy is further highlighted by the possibility to study dynamic processes, such as cancer cell phagocytosis mediated by fragment crystallizable (Fc) macrophage receptors.^[^ ^179^, ^180^ ^]^ During this process the fast actin cytoskeleton reorganization allows the proper engulfment of tumor cells,^[^ ^181^ ^]^ via the formation of cellular membrane protrusions. AFM was able to discern the morphological changes in cellular membrane ultra‐microstructures during the stages of macrophage phagocytosis.^[^ ^180^ ^]^ Beyond merely observing, several strategies have been proposed to enhance this recognition and attack approach, via increasing cancer cell roughness by incorporating gold nanoparticles to making them more susceptible to detection and engulfment by Fc macrophage receptors.^[^ ^182^ ^]^

Finally, the combination of AFM with machine learning has opened new avenues for differentiating cancer cell lines with different degrees of aggressiveness.^[^ ^183^ ^]^ For example, machine learning significantly enhanced the accuracy of identifying neoplastic malignancy levels of two genetically altered colon cancer cell lines (HT29 and Csk) from nearly 70% to 94%.^[^ ^184^ ^]^ Machine learning converted AFM imaging data to surface parameters, creating a database after training subsets to optimize their sensitivity and accuracy by cross‐validation.^[^ ^52^ ^]^ Furthermore, support vector machine algorithms could achieve up to 82.5% accuracy for prostate cancer cells, when logistic regression‐classified frameworks were based on the feature selection method.^[^ ^185^ ^]^ These compelling examples corroborate the robustness of data interpretation when nano‐imaging is combined with machine learning methods. This powerful synergy offers a promising tool to unequivocally distinguish healthy cells and tissues from their carcinogenic counterparts.

### 3.3 Nano‐Imaging of Biomolecular Processes in Neurodegeneration

During the onset of neurodegeneration, such as in the case of PD, HD, and AD, normally soluble peptides or proteins (such as Aβ, tau, α‐synuclein, and huntingtin) form a variety of nanoscale sized and heterogeneous soluble oligomeric intermediates and insoluble fibrillar amyloid aggregates, which have a high degree of polymorphism. These proteins lose their physiological role(s) and aggregate into species with potential novel and different neurotoxic functions related to their different shapes and content of cross‐β structure. The involved toxicity mechanisms include membrane permeabilization, calcium dysregulation, mitochondrial damage, inflammation, and oxidative stress.^[^ ^6^, ^17^, ^38^, ^124^ ^]^ Although oligomers are considered key actors, the nature of the aggregates most prone to cause cytotoxicity, and the mechanisms by which these aggregates contribute to disease are still unclear. Thus, it is necessary to interrogate the properties of the formed heterogeneous species to understand how they might be related to disease.

**Figure 3:**
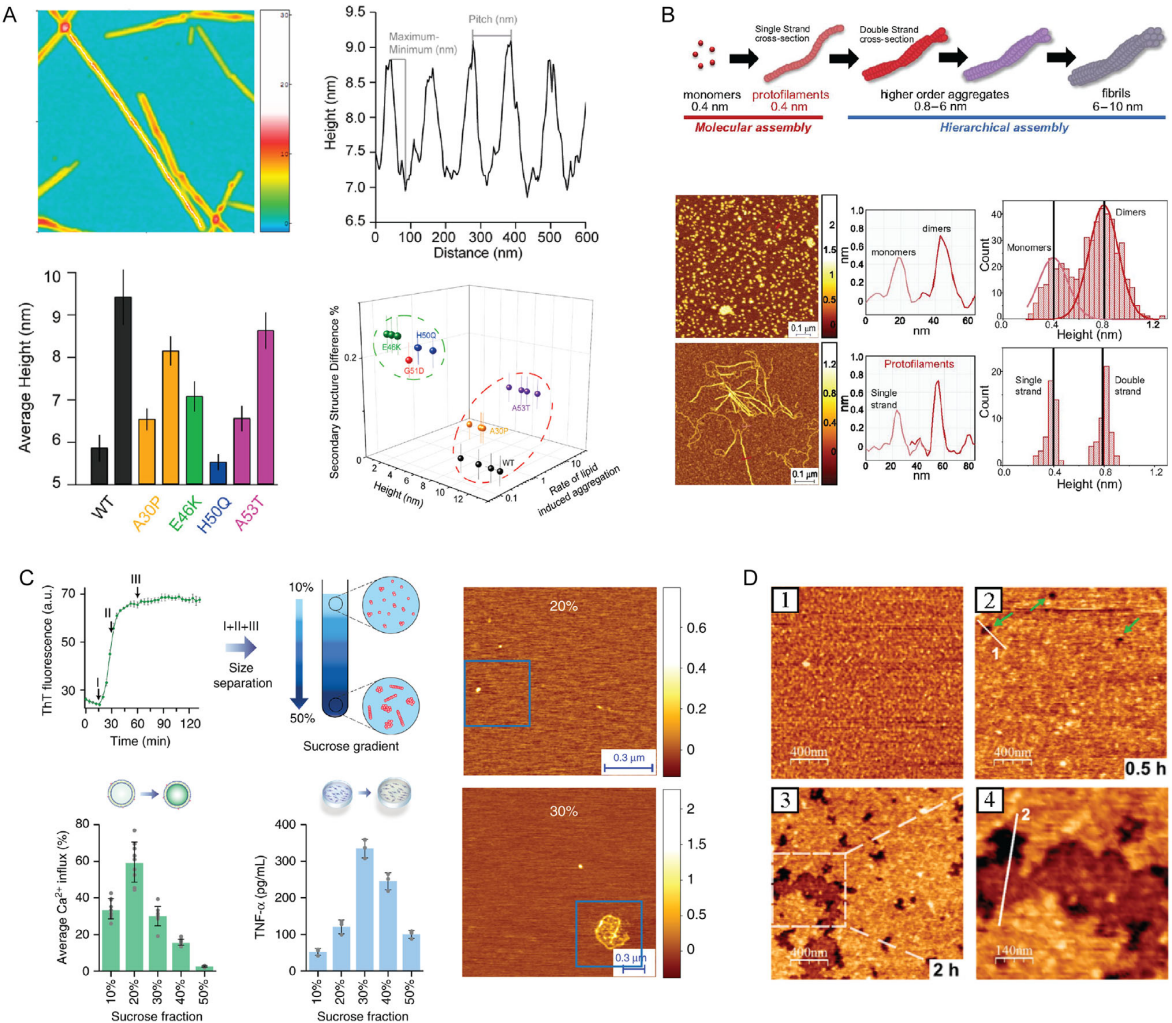
In vitro nano‐imaging AFM studies of amyloids. A) Polymorphism of the fibrils formed by WT α‐synuclein, and the mutational variants associated with familial PD, adapted and printed with permission from,^[^ ^88^ ^]^ Copyright 2020. American Chemical Society. B) Model of α‐synuclein fibril formation (adapted and printed with permission from,^[^ ^89^ ^]^ Copyright 2018. Proceedings of the National Academy of Sciences) showing the i) initial molecular assembly leading to the newly identified single‐strand protofilaments (in light red) and ii) subsequent hierarchical assembly of mature amyloid fibrils. Aggregation time course monitored by high‐resolution AFM imaging, details of the cross‐sectional height of oligomeric and protofibrillar species and single‐molecule statistical analysis, with the respective histogram distributions of the cross‐sectional height of the species observed. C) Aβ_42_ aggregates of diverse sizes exhibit different relative toxicity by distinct mechanisms studied by the lipid bilayer permeability and ELISA assays, adapted and printed with permission from,^[^ ^38^ ^]^ Copyright 2019. Springer Nature. D) Morphology of natural brain total lipid extracts layer 1) before and 2) after the 30 min exposure of S100A9. Green arrows indicate the formation of defects in the lipid layer. 3) Lipid layer after 2 h of S100A9 exposure. 4) Magnification area from the white dashed square area of (3). Printed with permission from,^[^ ^201^ ^]^ Copyright 2023, Elsevier.

**Figure 4:**
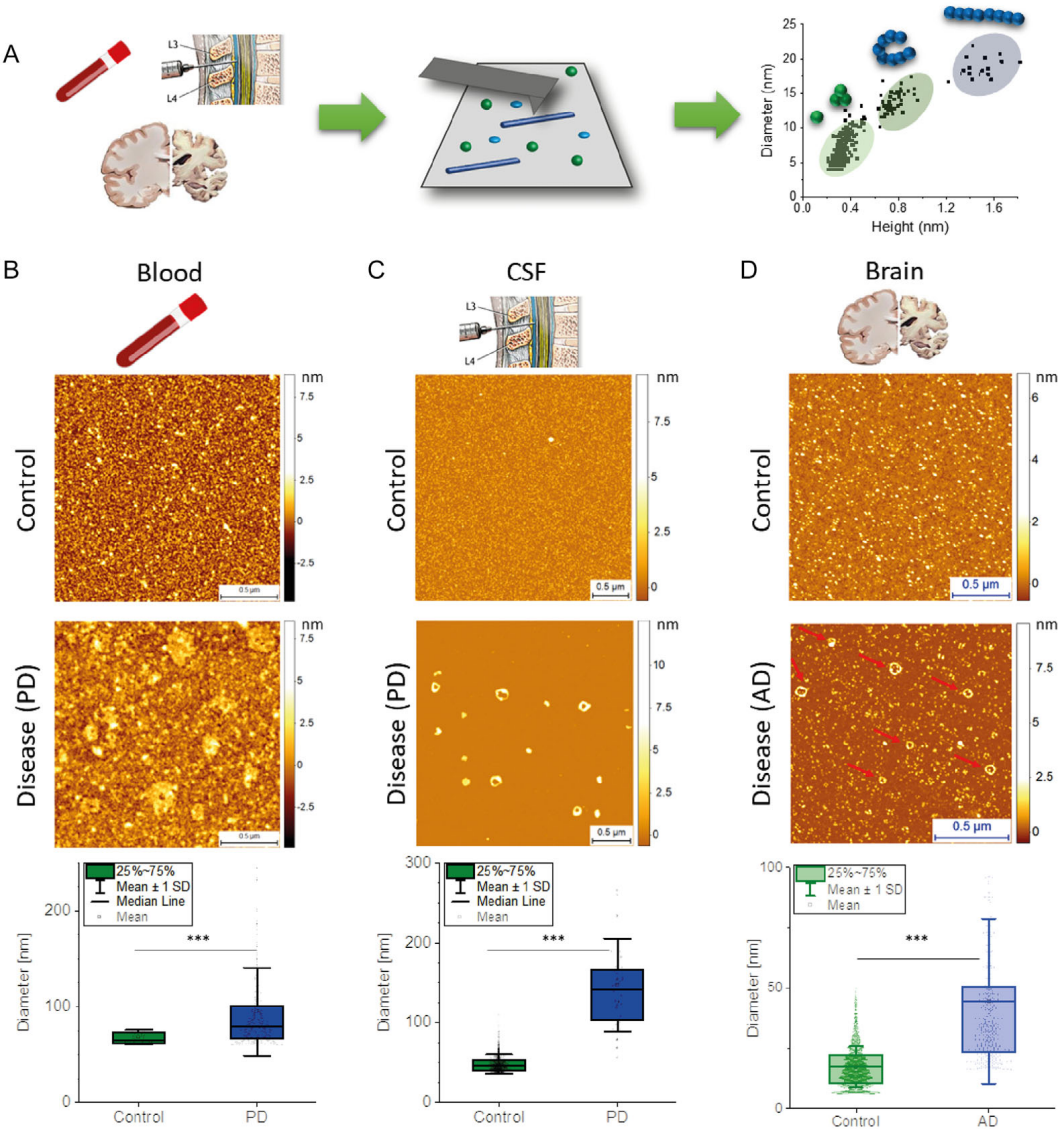
Ex vivo nano‐imaging of protein aggregation in human biofluids and tissue. A) AFM‐based nano‐imaging to detect and quantify amyloids shape/size in human samples. B) blood,^[^ ^40^ ^]^ C) cerebrospinal fluid (CSF),^[^ ^41^, ^97^ ^]^ and D) brain samples^[^ ^32^, ^33^ ^]^ of control versus disease (AD/PD) imaged with AFM single‐molecule analysis of the detected amyloids (red arrows, only diameter for simplicity). In all cases nano‐imaging statistically discriminated control versus disease human biofluids and tissues by evaluating the number and shape of protofilaments and spheroidal oligomers. Data adapted and printed with permission from;^[^ ^32^, ^40^ ^]^ Copyright 2021, Oxford Academy & Copyright 2022, Oxford Academy, respectively.

AFM offers an invaluable tool to carry out 3D morphological analysis at the single‐molecule level of the several amyloid oligomeric and fibrillar species, occurring during protein aggregation.^[^ ^186^ ^]^ The analysis can be performed with subnanometer resolution both in vitro (**Figure** **3**)^[^ ^88^, ^89^, ^96^ ^]^ and ex vivo biofluids and tissues (**Figure** **4**).^[^ ^32^, ^33^, ^40^, ^97^, ^98^ ^]^ Ex vivo, AFM has demonstrated to differentiate nanoscale‐sized amyloids in healthy controls versus disease patients with neurodegeneration, in the case of AD and PD. These ex vivo studies highlighted a direct correlation between the size and structure of amyloid fibrils and their toxicity. Thus, single‐molecule analysis of these species offers a powerful method to study the molecular mechanisms underlying the onset of neurodegeneration, with in vitro systems acting as ideal model systems. The general possibility of analyzing the subnanometric morphology at several time points during protein aggregation can shed light on the heterogeneity of the amyloid species formed and their pathway of formation. AFM can visualize the monomeric and early oligomeric forms present during the lag‐phase of the aggregation, and the structural properties of single‐amyloid fibrils.^[^ ^90^, ^186^ ^]^ By measuring height, length, diameter, and weight, AFM has demonstrated the ability to distinguish morphologically different populations, such as dimeric and trimeric aggregates, as well as the formation of multiple polymorphic fibrillar species. Within the tens of proteins and peptides associated to misfolding, aggregation and disease onset, AFM has empowered to study the oligomerization and fibrillation process of wild‐type and mutated forms of α‐synuclein, Aβ peptide, insulin, ataxin‐3, and huntingtin protein (Figure 3).^[^ ^90^, ^94^ ^]^

We first discuss in detail the case of α‐synuclein aggregation in the context of PD. AFM has enabled visualization of how the intermediate amyloid oligomeric species and pathway of aggregation leading to amyloid fibrils can be modified by i) point mutations (Figure 3A),^[^ ^88^, ^90^ ^]^ causing the early onset of PD, and ii) by ions and natural compounds.^[^ ^187^, ^188^ ^]^ The information provided by AFM is key to understanding how to develop drugs that can interfere with and possibly delay the process of aggregation and amyloid formation in vivo. Furthermore, AFM can provide resolved structural information to target amyloid formation using pharmacological approaches. AFM proved that mature amyloid fibrils are formed by the hierarchical self‐assembly^[^ ^189^ ^]^ of Ångström sized single‐strand protofilaments, which are oligomeric species forming both in vitro (Figure 3B) and in vivo biofluids (blood serum, cerebrospinal fluid (CSF), Figure 4A,B) causing high inflammatory response.^[^ ^33^, ^40^, ^89^ ^]^ These protofilaments are the smallest elementary unit in the hierarchical assembly of amyloid fibrils and are made of a monomeric chain, which further twists together through specific side chain interactions into higher‐order protofilaments, leading to cross‐β sheet and amyloid formation. Initially, α‐synuclein forms only monomers and dimers of heights about 0.4 nm and 0.8 nm, respectively.^[^ ^89^ ^]^ After incubation, α‐synuclein formed two polymorphs of elongated protofilament species, with subnanometer scale diameters and micrometers lengths. The first species had a cross‐sectional height of 0.3–0.4 nm, corresponding to the height of monomeric α‐synuclein. The second species had a cross‐sectional height of 0.7–0.9 nm, corresponding to the cross‐sectional height of the dimers. Larger incubation times showed that protofilaments are on pathway of mature amyloid fibril formation. A comparison of AFM measurements with single‐molecule fluorescence measurements indicated that the critical size for α‐synuclein to form a stable mature amyloid fibril is ≈70 monomers.^[^ ^190^ ^]^ Preventing aggregates from reaching this critical size may be a key cellular protective mechanism.

In the framework of AD, AFM imaging has also fundamentally contributed to elucidating the molecular processes underlying the misfolding, early aggregation, and fibrillization of Aβ_42_, Aβ_40_, and Aβ peptide fragments. Similar to PD, AFM has been used to detect in vitro (Figure 3C) and in vivo protofilaments (Figure 4C), protofibrillar aggregates, and oligomeric species, which have been suggested to be toxic via membrane permeabilization and enhancing inflammatory response.^[^ ^32^, ^38^, ^191^ ^]^ As a key finding, AFM was applied to support the proof that different Aβ aggregates give rise in vitro to cellular toxicity via different mechanisms, such as inflammatory response and membrane permeabilization (Figure 3C),^[^ ^38^ ^]^ which was confirmed by ex vivo analysis showing that soluble aggregates are present in the early stages of AD,^[^ ^32^ ^]^ and further change in size and mechanism of toxicity during AD progression.^[^ ^38^ ^]^ AFM was further applied to complement the mechanistic insights obtained by ThT chemical kinetics analysis to prove independently that secondary nucleation events are the key mechanisms dominating the kinetics of pathology‐related aggregation and fibrillization of Aβ_42_. For the first time, secondary nucleation events were directly visualized at the fibril surface, and the polymorphism of the aggregated fibrillar structures was successfully studied.^[^ ^192^ ^]^

The ability to investigate the properties of a single amyloid species as a function of time or external factors further empowers the possibility of understanding how the aggregation network or cytotoxic species could be targeted by pharmacological approaches. AFM has shown that the length of Aβ_42_ fibrils increases upon incubation after reaching the plateau phase of ThT kinetics.^[^ ^96^ ^]^ This time‐dependent change in the length of Aβ_42_ fibril growth up to 410 ± 30 nm after 24 h of incubation was also dependent on the media exchange and buffer conditions from HEPES to PBS.^[^ ^193^ ^]^ AFM was further used to study the stabilization of neurotoxic oligomers of Aβ_40_ by endogenous dopamine metabolites (DOPAL), suggesting that metabolite imbalance could influence the early development of AD.^[^ ^194^ ^]^ External agents not only affect fibril length, but also affect oligomeric and fibrillar diameters and overall shape. It was recently observed that oligomeric species incubated with different metal ions (Al^3+^, Cu^2+^, Fe^2+^, and Zn^2+^) differ in their structure and toxicity.^[^ ^195^ ^]^ In the presence of Zn^2+^ ions, the length distribution of Aβ_40_ fibrils was unchanged, but the height dimensions of Aβ_40_ features increased >3 fold. This increase in fibril diameter is due to Zn^2+^ ions promoting the hydrophobicity of Aβ_40_/Aβ_42_ fibrils, which directly impacts the aggregation tendency of these fibrils. Similarly, trodusquemine, a natural compound extracted from sharks, can enhance the rate of aggregation by promoting monomer‐dependent secondary nucleation, forming fibrils shorter in length but with higher diameters that correlate with reduced toxicity of the on‐pathway oligomeric aggregates.^[^ ^187^ ^]^

In relationship to the onset of genetic HD, AFM has been widely applied to study the effect of polyglutamine (polyQ) expansion and post‐translational modifications on the propensity of huntingtin protein aggregation.^[^ ^94^ ^]^ Indeed, HD is genetically caused by a polyQ expansion higher than a threshold of 36Q. To unravel differences in amyloid formation below and above the polyQ pathogenic threshold, AFM imaging showed that the aggregation propensity, smoothness, and structural order of amyloid fibrils increased as a function of the polyQ content.^[^ ^64^ ^]^ Further studies have shown that the aggregation propensity can be further regulated (accelerated or reduced) by post‐translational modifications in the N‐terminus of the huntingtin exon‐1.^[^ ^37^, ^93^, ^196^ ^]^

AFM has been further applied to investigate several other proteins, alone and co‐aggregating, and synergistic effects between proteins should not be underestimated. It was reported that calcium ions stabilize S100A9 protein structure and inhibit the formation of amyloid fibrils,^[^ ^197^ ^]^ but have an antagonistic effect on Aβ aggregates,^[^ ^198^ ^]^ and produce conformational changes in the β‐sheet content of superoxide dismutase‐1, which modulates fibril formation.^[^ ^199^ ^]^ The underlying mechanisms of Aβ_42_ fibril formation are indeed prompted by heteromolecular co‐aggregation reactions with S100A9, yielding fibrils with lengths of 750 ± 80 nm.^[^ ^193^ ^]^ Finally, AFM imaging was also used to determine the interaction and local aberrations of cellular membranes with amyloidogenic proteins. Lipid membranes rich in cholesterol promote Aβ_42_ aggregation, although AFM proved that the structural properties of the produced amyloid fibrils were not altered.^[^ ^200^ ^]^ The natural brain total lipid extract (BTLE) membrane model (Figure 3D) is negatively affected by the presence of S100A9 protein.^[^ ^201^ ^]^ Small holes were created on the BTLE membrane 30 min after the injection of S100A9 causing extensive loss of lipid membrane integrity 2 h after S100A9 addition, suggesting a neurotoxic action of S100A9.

## 4 High‐Speed Nano‐Imaging via AFM

Biomolecules exhibit inherent structural dynamics and undergo rapid conformational alterations in response to their interactions with other biomolecules or various physicochemical stimuli.^[^ ^202^ ^]^ The comprehensive elucidation of these conformational dynamics is of paramount importance as it unveils the potential underlying mechanisms implicated in prevalent human diseases, including, but not limited to, cancers and neurodegenerative disorders. We have shown in chapter 3, that conventional AFM is suitable for high‐resolution imaging. Nonetheless, the temporal resolution of conventional AFM is suboptimal when aiming to record dynamic of biomolecules. Advances in high‐speed AFM (HS‐AFM) have recently addressed these limitations.^[^ ^58^, ^203^ ^]^ HS‐AFM has opened the possibility of performing video‐rate nanoimaging to examine intricate molecular properties, biomolecular interactions, and modifications in organelle structure and function within the realms of both cancer and neuroscience research.^[^ ^204^, ^205^, ^206^, ^207^ ^]^

### 4.1 Principles

Various sophisticated structural methodologies have been devised to examine the native structure and conformational dynamics of biomolecules.^[^ ^208^ ^]^ The objective was to achieve real‐time and real‐space visualization of the dynamic behavior of biomolecules under physiological environments.^[^ ^202^ ^]^ Among these methodologies, advanced techniques, such as cryo‐electron microscopy (cryo‐EM) and X‐ray crystallography,^[^ ^209^, ^210^ ^]^ offer a high level of precision in capturing the structural details of biomolecules. However, despite their excellent performance, these methodologies are constrained by their static nature, which limits their capacity to portray the structures of the swiftly evolving and transient intermediates.^[^ ^211^ ^]^ In contrast, structural methodologies, such as nuclear magnetic resonance (NMR) and Förster resonance energy Transfer (FRET),^[^ ^208^, ^212^ ^]^ can discern rapid conformational changes in biomolecules. However, these tools are inherently limited in their ability to achieve direct real‐time visualization of dynamic biomolecules. Consequently, computational simulations are indispensable to faithfully recapitulate the innate dynamic properties of biomolecules.^[^ ^213^, ^214^ ^]^ The synergy between experimental techniques and computational approaches is crucial for a comprehensive understanding of the intricate conformational dynamics of biomolecules.

HS‐AFM works similarly to AFM (paragraph 4), but incorporates a dynamic feedback controller and uses a short cantilever endowed with a small spring constant and high resonance frequency in fluid conditions. This combination renders HS‐AFM proficient for fast scanning.^[^ ^203^ ^]^ The dynamic feedback controller mitigates tip‐sample interactions, thereby diminishing mechanical stress.^[^ ^203^ ^]^ In addition, it precludes the dissipation of elevated temperatures resulting from cantilever oscillations, thereby safeguarding the integrity of the sample during the scanning process.^[^ ^203^ ^]^

High‐speed atomic force microscopy (HS‐AFM) operates in amplitude modulation (AM) mode.^[^ ^215^ ^]^ Unlike conventional setups, the cantilever in HS‐AFM is oriented upward, facing the sample. It oscillates vertically near its resonant frequency under piezoacoustic or photothermal excitation. As in conventional imaging, the tip intermittently contacts the sample surface leading to a reduction of its oscillation amplitude. In addition to raster scanning in the x–y plane, vertical (z‐axis) scanning is regulated by a fast feedback loop that maintains a constant cantilever amplitude at a predefined setpoint, thereby ensuring stable tip–sample contact forces.

All HS‐AFM components are engineered for rapid responses to accommodate high‐speed scanning. In aqueous environments, a common choice for imaging are short silicon nitride cantilevers, which exhibit resonance frequencies between 400 and 1200 kHz, with spring constants of k = 0.1–0.2 N/m and quality factors of Q = 1–2. The z‐scanner also possesses a high resonance frequency, typically in the 200–400 kHz range.^[^ ^215^ ^]^ By applying control on quality factor Q‐control, the cantilever's quality factor is reduced to 1–2, enabling rapid response to excitation signals. This combination renders HS‐AFM proficient for fast scanning.^[^ ^203^ ^]^

To maintain consistent tip–sample interaction even over topographically complex surfaces, a dynamic proportional–integral–derivative (PID) controller adaptively tunes its gain in real time.^[^ ^215^ ^]^ Gentle tip–sample contact is achieved by limiting the energy dissipation per contact event to below a few units of thermal energy k_B_T. The energy lost from the vibrating cantilever is transiently transferred to the sample and rapidly dissipated into the surrounding buffer, preventing local heating or sample damage. Under these conditions, the tip can tap the sample surface multiple times without compromising its structural integrity. The dynamic feedback controller mitigates tip‐sample interactions, thereby diminishing mechanical stress.^[^ ^203^ ^]^ In addition, it precludes the dissipation of elevated temperatures resulting from cantilever oscillations, thereby safeguarding the integrity of the sample during the scanning process.^[^ ^203^ ^]^

HS‐AFM has further undergone enhancements encompassing a significantly enlarged scanning area at the micron scale with megapixel resolution^[^ ^216^ ^]^ and unprecedented scanning speeds down to the order of magnitude of 50 milliseconds for a 100 × 100 pixel image.^[^ ^58^ ^]^ Furthermore, HS‐AFM has been integrated with computational analyses, augmenting its imaging capabilities using a synergistic approach.^[^ ^217^ ^]^ The superior spatiotemporal resolution of HS‐AFM facilitates direct, real‐time visualization of biomolecules,^[^ ^74^ ^]^ organelles,^[^ ^218^, ^219^ ^]^ and cellular entities.^[^ ^220^ ^]^ This capability enables an in‐depth investigation of their native conformation, intrinsic structural dynamics, molecular interactions, organelle and cellular functions.

### 4.2 Video‐Rate Imaging of Biomolecular Processes in Cancer

Carcinogenesis is a multifaceted pathogenic process characterized by the simultaneous occurrence of various aberrations, collectively steering the acquisition of cancer hallmarks, including oncogenic fusion, histone modifications, and nucleus morphological differences. HS‐AFM has empowered to visualize these dynamic processes with high spatiotemporal resolution.

Oncogenic fusion proteins are prevalent in numerous cancers and emerge as consequence of diverse processes including chromosomal translocation, errors in DNA replication and repair, and aberrations in RNA splicing.^[^ ^15^ ^]^ The genes involved in many oncogenic fusion proteins are primarily kinases and transcription factors.^[^ ^103^ ^]^ The classic fusion of two kinase genes, *BCR* and *ABL*, forms a constitutively active kinase protein that drives leukemic cell proliferation in 95% of chronic myeloid leukemia patients.^[^ ^103^ ^]^ The discovery of BCR‐ABL tyrosine kinase inhibitors, Imatinib (Gleevec), has driven the progression of molecular‐targeted therapies against cancers.

**Figure 5:**
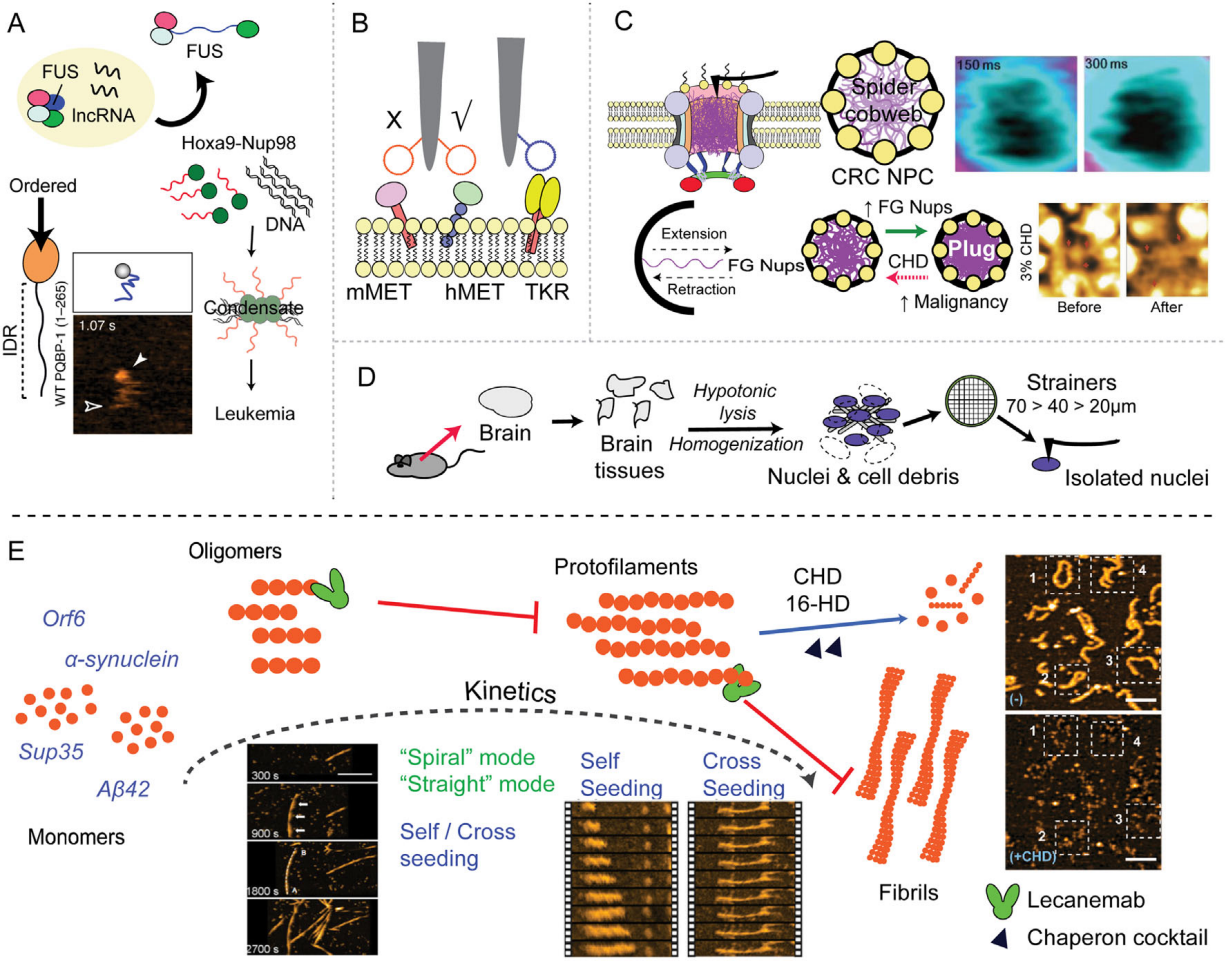
Video‐rate nano‐imaging by AFM in cancer and neurodegeneration. Cancer: A) Spatiotemporal resolution of HS‐AFM enabled visualization of intrinsically disordered regions (IDRs) of proteins.^[^ ^36^ ^]^ For instance, to investigate the mechanism of fusion cancer‐related proteins with IDRs, such as HOXA9‐Nup98, driving oncogene expressions by forming liquid condensates; adapted and printed with permission from,^[^ ^205^ ^]^ Copyright 2021, Springer Nature. B) Functionalized cantilevers could be used for single molecule‐recognition of tyrosine kinase receptors (TKRs) that are frequently overexpressed in cancer cells, such as macrocyclic aMD4 conjugated cantilever only recognizes human MET receptor (hMET) but not mouse MET receptor (mMET). Adapted and printed with permission from,^[^ ^377^ ^]^ Copyright 2021, American Chemical Society. C) Aberrant nuclear structures, including nuclear pore complex (NPC), were commonly found in cancer cells, and HS‐AFM was used to study NPC of colorectal cancer (CRC) cells. Results demonstrated that FG‐Nups in central channel resembled spider cobweb; printed with permission from,^[^ ^218^ ^]^ Copyright 2017, American Chemical Society. Dynamic extension and retraction of FG‐Nup filaments have been compared between CRC and normal colon cells. Furthermore, FG‐Nups underwent liquid–liquid phase separation (LLPS) to form a central plug to accelerate nuclear transport to enhance cancer malignancy. Disruption of central plug by an anti‐LLPS chemical, CHD, was filmed in a real‐time manner; printed with permission from,^[^ ^219^ ^]^ Copyright 2020, Elsevier. D) A rapid nuclei isolation protocol was further developed to isolate nuclei from tissues for HS‐AFM imaging. This protocol could be feasible to isolate nuclei from clinical samples of cancer patients for nanoscopic imaging; printed with permission from,^[^ ^378^ ^]^ Copyright 2024, Multidisciplinary Digital Publishing Institute. Neurodegeneration: E) HS‐AFM was applied to investigate amyloid fibrillation, including polymorphism and secondary nucleation phenomena (self‐seeding or cross‐seeding) in fibril elongation and stabilization; printed with permission from,^[^ ^225^, ^226^ ^]^ Copyright 2016, Proceedings of the National Academy of Sciences & Copyright 2020, American Chemical Society, respectively. HS‐AFM was similarly used to study the dynamic formation of cytotoxic fibrils that cause NDs or neurological complication of COVID‐19 could be observed via HS‐AFM; printed with permission from,^[^ ^228^ ^]^ Copyright 2023, American Chemical Society. HS‐AFM was performed to elucidate the effects of potential antibodies,^[^ ^95^ ^]^ chaperones,^[^ ^229^ ^]^ and chemicals^[^ ^228^ ^]^ (1,6‐HD, CHD) in eliminating the cytotoxic oligomers, protofilaments, or fibrils.

HS‐AFM nano‐imaging has enabled the study of the nanoscale conformational dynamics of oncogenic fusion proteins, particularly their response to potential drug compounds. Oncogenic fusion proteins with transcription factor genes alter chromatin organization, driving carcinogenesis by either promoting oncogene expression or suppressing tumor suppressor genes. Robust observation of dynamic interactions between RNA/DNA‐binding proteins and nucleic acids can now be recorded using HS‐AFM (**Figure** **5**A), as for the dynamic conformational changes of FUS protein during interactions with noncoding RNAs.^[^ ^36^ ^]^ DNA/RNA binding proteins are involved in certain cancers; hence, the insights from FUS protein interactions with long noncoding RNA (lncRNA) or promoter‐associated noncoding RNA (pncRNA) may offer crucial information about its role in regulating the post‐DNA damage cell cycle.^[^ ^36^ ^]^ Studies have shown that transcription factors fuse with nontranscription factor intrinsically disordered regions (IDRs), forming liquid condensates via liquid–liquid phase separation in the nucleus. For instance, FUS (IDR segment) was fused with DDIT3 (DNA‐binding domain), and Nup98 (IDR segment) was fused with HOXA9 (DNA‐binding domain).^[^ ^103^ ^]^ These condensates created isolated regions for enhanced oncogene expression. Moreover, multiple enhancers can gather into condensates enabling cooperative activity that amplifies oncogenic transcription. Such clusters are known as super enhancers. Coactivators with IDRs such as BRD4 and MED1 undergo phase separation to form condensates, thereby compartmentalizing super‐enhancers and boosting transcription activation.^[^ ^221^ ^]^ Interestingly, certain enhancers sequestered within condensates are associated with radio resistance in cancer cells.^[^ ^222^ ^]^

HS‐AFM with its exceptional spatiotemporal resolution has further enabled real‐time imaging of IDR dynamics under physiological buffer conditions.^[^ ^205^ ^]^ Indeed, characterizing IDRs presents significant challenges for conventional structural approaches such as cryo‐EM and X‐Ray crystallography, as these regions typically lack fixed structures. Moreover, the lack of direct visualization and limited temporal resolution of conventional approaches hinder our understanding of the dynamic processes underlying condensate formations. HS‐AFM was successfully applied to visualize both reversible and irreversible condensate formation events, such as those involving histone H2A‐DNA interactions.^[^ ^206^ ^]^ By capturing the transient and interchangeable conformations of disordered proteins, HS‐AFM nanoimaging has offered critical insights into the biophysical properties of condensates and their formation, thereby providing a valuable platform for evaluating therapeutics targeting pathogenic condensates associated with cancer progression and metastasis (Figure 5A). In epigenetic regulation of gene expression, post‐translational modification of histone tails further plays a critical role in modulating nucleosome dynamics, thereby influencing transcription activation or repression. Nonetheless, direct visualization of the effects of histone modification on nucleosome behavior has remained a major challenge for conventional structural and imaging approaches. Recent advances using HS‐AFM have enabled nanoscopic observation of dynamics of nucleosomes,^[^ ^220^ ^]^ providing new opportunities to elucidate the structural consequences of histone modification. These studies have uncovered key biophysical changes in nucleosome architecture and dynamics, offering deeper insight into the relationship between epigenetic modification and cancer.

Tyrosine kinase receptors (TKRs) are also often overexpressed in cancers to promote proliferation and to inhibit cell death. While TKRs expression can be evaluated via immunohistochemistry, conventional methods are limited in resolving single‐molecule conformation and dynamics, especially dimerization events on the cell membrane. To address this challenge, a synthetic macrocyclic peptide (aMD4)‐conjugated cantilever (Figure 5B) was used for HS‐AFM nanoimaging for rapid, single‐molecule recognition imaging of the human tyrosine kinase hepatocyte growth factor (hMET).^[^ ^221^ ^]^ The results of this study suggest that HS‐AFM could be used as nanoplatform for TKR inhibitors screening and as a molecular diagnostic tool for precise detection of TKR expression and dynamics on the surface of cancer cells.

Cancer cells also exhibit distinct morphological differences in their organelles, particularly in their nuclei. These nuclei often display abnormal shapes, pleomorphisms, and increased chromatin densities. The pathological hallmark is a high nucleus‐to‐cytoplasm (N/C) ratio, reflecting the hyperproliferation state of cancer. These features suggest that carcinogenesis may affect nuclear integrity. Early studies employing AFM compared the differences in the stiffness of nuclei between cancer and normal cells. In addition to altered nuclear physical properties, signal transduction between the cytoplasm and nucleus is often disrupted in cancer. Nucleocytoplasmic trafficking is orchestrated by nuclear pore complexes (NPC) embedded in the nuclear envelope (Figure 5C). The mega‐Dalton NPC, comprising of over 30 nucleoporins (Nups), uses specific Nups with phenylalanine‐glycine repeats (FG‐Nups) to establish a selective nanogate. Large biomolecules, forming complexes with nuclear transport receptors (karyopherin α and β), subsequently engage with FG‐Nups for nuclear entry or exit. AFM was first used to apply forces on the breast cancer cell line (MCF10A), revealing that NPC stretch enhances the nuclear entry of YAP protein, and indicating the NPC's mechanosensing regulatory capability.^[^ ^223^ ^]^ However, the temporal constraint of AFM for real‐time recording was limited to the observation of NPCs during nucleocytoplasmic transports, such as the flexibility of pore size, dynamics of FG filaments, and conformational dynamics of NPCs.

To overcome this limitation, HS‐AFM was used to unveil the highly dynamic nature of FG‐Nups and real‐time central plug formation in the *Xenopus laevis* oocyte NPC on a millisecond timescale.^[^ ^207^ ^]^ This approach revealed that the cytoplasmic orifice was lined with highly dynamic, flexible FG Nups that rapidly extend and retract. These FG Nups transiently entangle in the central channel without forming a stable meshwork, suggesting that the NPC barrier consists of fluctuating FG Nups appearing as a central plug when averaged over space and time. HS‐AFM nanoimaging was performed on NPC of nuclei isolated from colorectal cancer cell line, HCT116, revealing the structure of FG Nups in central channel of NPC, resembling the “spider‐cobweb” model. They also reported that FG‐Nup barriers in dying cells were deformed in response to anti‐cancer drug MLN8237/alisertib.^[^ ^218^ ^]^ These findings highlighted the potential of HS‐AFM as intracellular nano‐endoscopy tool, which may be useful for the development of personalized, nucleus‐targeted nanodrug delivery systems. In a subsequent study,^[^ ^219^ ^]^ HS‐AFM was employed as a spatiotemporal tracking method to visualize FG‐Nups organization in NPCs of CRC cells and organoids (Figure 5C). Single‐filament tracking revealed heterogeneous thickness between normal colon and CRC models, influencing filament motions. Given FG‐Nups overexpression in cancers, treatment with the LLPS inhibitor *trans*‐1,2‐cyclohexanediol reduced central plug size with limited reversibility in aggressive models, supporting a reversible FG‐Nup self‐assembly model underlying partial plug formation (Figure 5C). HS‐AFM enables direct tracking and manipulation of a native FG‐Nup filaments, long considered elusive. In addition to FG Nup dynamics, NPC components could function as tethering sites for super enhancers (SEs), thereby influencing target gene expression by facilitating mRNA export. Further studies also used HS‐AFM imaging to capture the structural dynamics of Nup153 IDR, which trapped SEs and upregulated oncogene TP63 expression through mRNA export.^[^ ^224^ ^]^ Recently, rapid strainer microfiltration (RSM), a rapid nuclei isolation technique, was developed to isolate nuclei from mouse brain tissue for HS‐AFM nanoimaging (Figure 5D). These results highlighted the possibility of HS‐AFM nanoimaging of nuclei from tumor samples of patients with cancer for research or diagnostic purposes.

### 4.3 Video‐Rate Imaging of Biomolecular Processes in Neurodegeneration

The high spatiotemporal resolution of HS‐AFM has made it a powerful tool for elucidating fundamental questions in amyloid research by enabling direct, real‐time visualization of structural dynamics of amyloid species at the nanoscale, which is imperative for tackling neurodegenerative diseases (Figure 5E).

HS‐AFM nano‐imaging has revealed the structural dynamics of Aβ_42_ oligomeric states, such as trimers, pentamers, and heptamers, which may be implicated via different neurotoxic effects to the onset of AD.^[^ ^204^ ^]^ To contribute to this overcome this knowledge gap, HS‐AFM has been used to investigate whether low and high molecular weight Aβ_42_ peptides follow distinct self‐assembly pathway, uncovering divergent mechanisms of real‐time aggregation.^[^ ^225^ ^]^ HS‐AFM has also proven capable of capturing the progressive transition of amyloidogenic oligomers into mature fibrils. This capability was exemplified in studies of the yeast prion Sup35, where both the oligomer‐to‐fibril transformation and subsequent fibril growth were visualized in real time.^[^ ^91^ ^]^

HS‐AFM has further provided insights into the polymorphism of amyloid fibrils, capturing structural heterogeneity such as helical/spiral, rod‐like, and hybrid morphologies, thereby helping answer how fibril architecture varies with environmental and molecular conditions.^[^ ^225^ ^]^ Beyond the final fibrillar structure, HS‐AFM enables the dynamic tracking of fibrillation, revealing how factors like ionic strength influence aggregation pathways. For example, experiments using KCl or NaCl buffers showed that KCl promoted both helical and rod‐like fibrils, whereas NaCl favored predominantly helical structures.^[^ ^225^ ^]^ These findings raise important questions about the role of monovalent cations in modulating amyloid fibrils morphology, formation and their stability.

Furthermore, HS‐AFM identified two distinct seeding mechanisms of α‐synuclein: self‐seeding and cross seeding, highlighting their differential effects on fibril elongation kinetics and structural integrity.^[^ ^226^ ^]^ Such insights could help dissect the molecular basis of strain variability and propagation of synucleinopathies. By answering critical questions about the kinetics, pathways, and structural diversity of amyloid assembly, HS‐AFM provides key molecular underpinnings of protein misfolding diseases with unprecedented resolution.

Specific viral proteins are also amyloidogenic, potentially contributing to neurological complications observed in some viral infections. SARS‐CoV‐2 ORF6 demonstrated self‐assembly behavior, forming protofilaments that were also internalized by lung cancer cells (PC9 and A549) and subsequently aggregated within the cellular environment. These findings raise key mechanistic questions about viral protein misfolding, aggregation, and interaction with host machinery, and their potential roles in long‐term neurological symptoms, such as brain fog and dementia in post‐COVID‐19 patients.^[^ ^227^ ^]^ HS‐AFM has offered a powerful nanoscopic platform to address these questions by enabling real‐time visualization of ORF6 oligomerization dynamics, elucidating intermediate structures, and characterizing the kinetics of protofilament formation under near‐physiological conditions.^[^ ^228^ ^]^ This capability has provided critical insights into the molecular basis of virus‐induced neuropathology and may inform the development of therapeutic strategies targeting protein aggregation.

HS‐AFM can finally serve also as a nanoscopic platform to assess the effectiveness of potential therapeutic monoclonal antibodies or drugs in modulating or inhibiting aggregates formation or allowing the degradation of toxic protein aggregates (Figure 5E). For example, HS‐AFM has revealed that the monoclonal anti‐Aβ antibody, Lecanemab binds to oligomers and protofilaments, thereby preventing the formation of larger Aβ aggregates, shedding light on its mechanism of action and raising the question of how specific antibody‐aggregate interactions determine therapeutic efficacy.^[^ ^95^ ^]^ Similarly, nano‐imaging showed that a combination of three molecular chaperones (SP70, HSP40, and HSP110) effectively degraded α‐synuclein protofilaments without accumulating cytotoxic oligomeric intermediates,^[^ ^229^ ^]^ answering critical questions about how proteostasis network dismantle pathological aggregates. Finally, HS‐AFM nanoimaging demonstrated that SARS‐CoV‐2 ORF6 protofilaments were sensitive to aliphatic alcohols, urea, and SDS, implicating hydrophobic interactions as essential to protofilament stability.^[^ ^228^ ^]^ These findings highlight the power of HS‐AFM in deciphering the molecular basis of protein aggregation and in evaluating the mode of action to therapeutic interventions at nanoscopic resolution.

## 5 Force Spectroscopy and Molecular Recognition Imaging

Adhesion forces have a strong significance in the pathogenesis of cancer and amyloidogenic diseases, and they can be used to identify the fingerprints of these diseases by molecular recognition. In AFM force spectroscopy (AFM‐FS), a flexible cantilever functions as a force sensor to identify, image, and measure molecular interactions at the nanoscale.^[^ ^57^, ^145^ ^]^ By measuring the deflection of the cantilever in real time during data acquisition, it is possible to detect and study these interactions with high specificity and piconewton sensitivity. AFM‐FS measurements revealed that the modulation of mechanical forces is a key determinant in the molecular progress of neurodegenerative^[^ ^230^ ^]^ and cancer diseases.^[^ ^85^ ^]^ Thus, understanding the interaction forces is essential for discerning the underlying mechanisms of cancer and amyloidogenic diseases, which may further contribute to the development of innovative therapies against these disorders. Table 1, Supporting Information, shows key mechanostability parameters found in biological systems associated to these malignancies.

### 5.1 Principles

**Figure 6:**
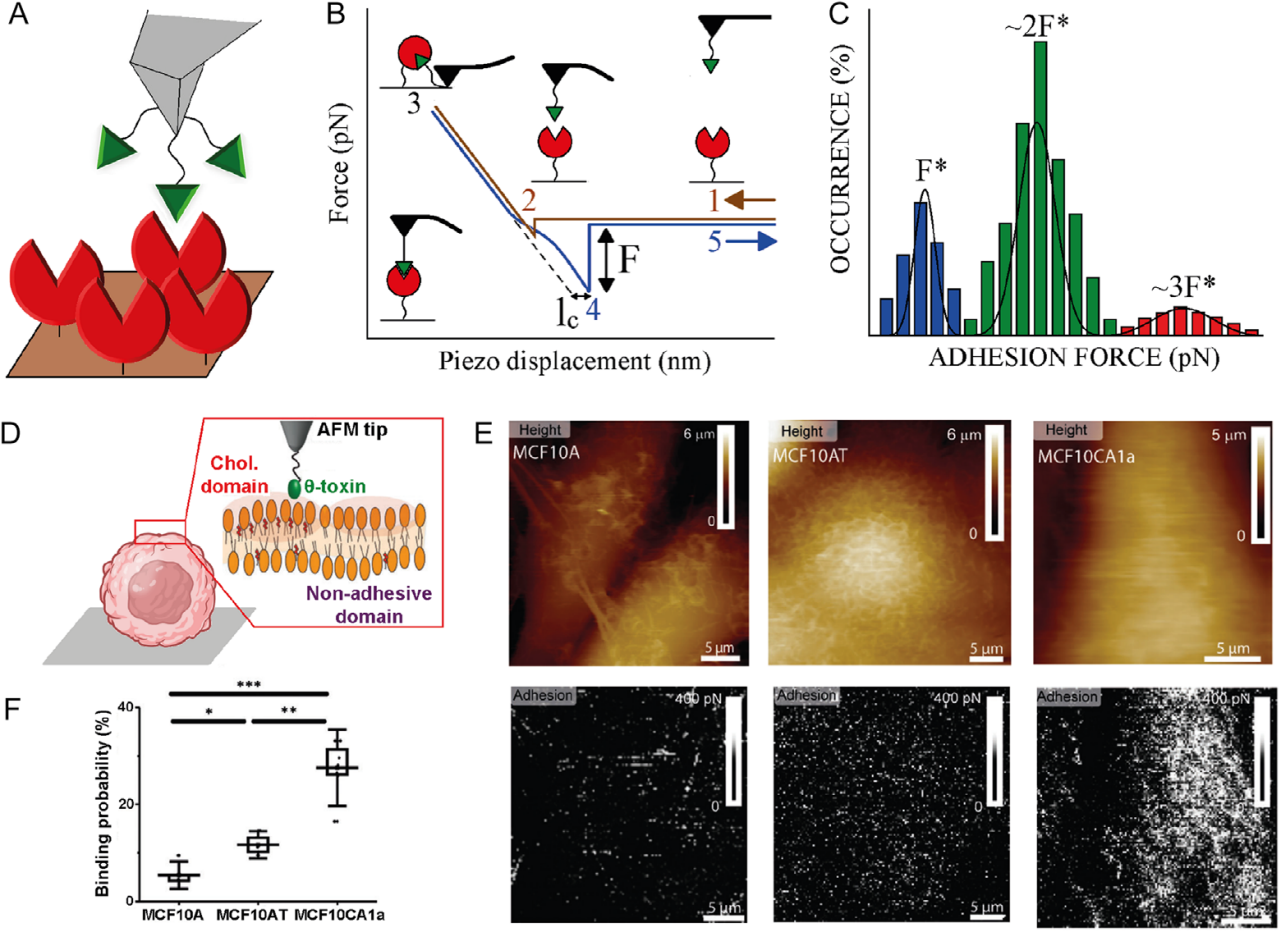
Force spectroscopy and molecular recognition in cancer research. A) Schematic representation of the location concerning the biomolecule attached on the surface (red “pac‐man” shape) and the partner linked onto the AFM tip (green triangles). B) Force‐distance curve profile for a specific tip‐sample unbinding event (point 4). Approach and withdrawal cycles indicated by the brown and blue arrows, respectively. *F* and *lc* correspond to the rupture force of the complex formed and the length once the flexible linker molecule is fully stretched, respectively. C) Statistical analysis devoted for the unbinding force data gathered in AFM‐FS experiments. Multievents (indicated by the green and red bars corresponding to the simultaneous breakage of two and tree complexes, respectively) can be observed. *F* ^*^ is the most probable rupture force related to one single complex. D) Representation that illustrates the AFM tip bioconjugation with θ‐toxin facing the plasma membranes of breast cancer cells. E) Representative topography and adhesion maps acquired simultaneously from the same region of MCF10A, MCF10AT, and MCF1°CA1a breast cancer cells. F) Binding probability distributions calculated for MCF10A, MCF10AT, and MCF1°CA1a cells by the respective force‐distance curves. D–F) printed with permission from,^[^ ^75^ ^]^ Copyright 2020, Wiley.

Before AFM‐FS experiment can start to sense and recognize the biomolecular process of interest, it is necessary to design smart bioconjugation strategies to covalently attach biomolecules or cells to the AFM tip and on a flat substrate surface for analysis^[^ ^151^ ^]^ (**Figure** **6**A).

Many subsequent chemical steps must be devoted to the site‐directed attack of the target moieties required to test cancer cells^[^ ^231^ ^]^ or amyloid proteins.^[^ ^232^ ^]^ The most common process is the amination in the vapor phase to activate AFM tips made of silicon nitride (Si_3_N_4_), rendering free amine (‐NH_2_) groups at the most external surface.^[^ ^233^ ^]^ A broad variety of short‐length heterobifunctional linker molecules are also commercially available, and their choice is directly linked to the selected protocols related to bioconjugation of the biological entities of interest, surface modification, and AFM tip functionalization.^[^ ^60^ ^]^ Typically, the reactivity of hydroxyl (‐OH), carboxylic acid (‐COOH), and aldehyde (‐CHO) groups is exploited using *p*‐maleimidophenyl isocyanate (PMPI),^[^ ^234^ ^]^ 1‐ethyl‐3‐(3‐dimethylaminopropyl)carbodiimide (EDC),^[^ ^179^ ^]^ and 3‐(2‐pyridyldithio)propionyl hydrazide (PDPH)^[^ ^235^ ^]^ linker molecules to form stable urethane, amide, and hydrazone bonds, respectively. Alternatively, amine (‐NH_2_) and sulfhydryl (‐SH) chemical moieties can share the same linker molecule sulfosuccinimidyl 6‐[3´‐(2‐pyridildithio)propionamido] hexanoate (Sulfo‐LC‐SPDP)^[^ ^233^ ^]^ to create covalent amide and disulfide bonds, respectively. The coupling agents described above can include flexible polyethylene glycol (PEG) tethers with specific contour stretching lengths (*l*_*c*_),^[^ ^236^ ^]^ which will favor encountering biomolecular receptors and recognition events. Thus, it is necessary to devise optimized functionalization procedures to avoid detrimental cluster aggregations of cellular membrane proteins^[^ ^237^ ^]^ and to properly orient the recognition interfaces of the modified substrate towards the biological entities attached to the AFM tip to enhance the binding probability compared to classical random functionalization strategies.^[^ ^233^ ^]^

After successful tip functionalization, cantilever calibration is required for an accurate quantification of tip‐sample force of interaction. The deflection sensitivity and the spring constant of the AFM cantilever are determined via the slope of the approach force‐distance curve profile acquired on a stiff substrate surface (e.g., freshly cleaved mica or sapphire) and by the thermal or Sader methods, respectively. This enables to transform the photodiode voltage signal to the unit of force (N). AFM‐FS experiments could then be conducted via continuous approach and withdrawal cycles of the cantilever probe at local regions of the surface. These cycles are used to record force‐distance curves (Figure 6B). This is particularly relevant for identifying individual cellular protein receptors.^[^ ^238^ ^]^ During each cycle, AFM tip moves closer to the sample surface until it senses attractive van der Waals forces and jumps‐to‐contact (Figure 6B, points 1–2). Then, the electrostatically repulsive forces from the sample surface start to bend the flexible AFM lever until the tip‐sample contact takes place (Figure 6B, point 3). If a biological complex is formed, the crosslinker molecule will be stretched following a worm‐like chain (WLC) model behavior (Figure 6B, point 4).^[^ ^239^ ^]^ When the AFM lever moves away from the sample surface, the complex dissociates and the rupture force is recorded (Figure 6B, points 4–5). Thus, the WLC model acts as a fingerprint to differentiate between specific and nonspecific tip‐sample interactions once the force‐distance baseline is corrected.^[^ ^240^ ^]^ The intermolecular force value is directly proportional to the number and strength of the noncovalent bonds involved in the formed complex which are classified as London dispersion forces, hydrogen bonding, ion‐dipole, and dipole–dipole interactions.

A large volume of force‐distance curves must be recorded because the formation of molecular complexes is stochastic in nature. Only biological matters with a proper orientation will successfully recognize their partner. Multiple complexes could be obtained on the decorated substrate surface during a single approach retraction cycle of the functionalized tip. The most probable unbinding force of a single complex (F*) is then revealed after statistical analysis of all specific force events (Figure 6C), and this value should agree with blocking experiments adding an excess of free‐receptor entities to the solution. Blocking experiments not only favor the formation of a single complex but also minimize the ligand‐binding recognition probability.^[^ ^241^ ^]^ Thus, molecular recognition plays a pivotal role in biology driving the specific interactions among biomolecules that are involved in cellular communication processes.

To map distribution of molecular interactions, F‐V measurements enable the direct interrogation of these specific unbinding forces,^[^ ^242^ ^]^ by dividing the region of interest in an array of pixels where a single force‐distance curve is acquired in each one of them. The main shortcoming of F‐V experiments are the long data acquisition times and the low lateral resolution. To surpass this bottleneck many approaches were developed in this field, such as multiparametric imaging and simultaneous topography and recognition imaging (TREC).^[^ ^143^, ^243^, ^244^ ^]^ TREC is a dynamic mode where the cantilever is excited near its resonance frequency,^[^ ^243^ ^]^ at frequencies slightly below the resonance frequency of the cantilever, and its downward deflections are only affected by the tip‐sample surface contact, whereas the upward cantilever deflections are affected by the specific binding tip‐sample interactions. These interactions between the biomolecules tethered on the AFM tip and the receptors immobilized onto the solid surface take place during the lateral scan, leading to an oscillation amplitude reduction of the cantilever and a subsequent recording of the recognition event. Decoupling this information is possible to obtain simultaneously the topography and recognition maps. This approach has been broadly used to discern the molecular interactions at molecular^[^ ^244^ ^]^ and cellular^[^ ^245^ ^]^ level.

Working under the repulsive electrical double layer (REDL) regime when low applied forces are applied also yields molecular recognition imaging (MRI) studies.^[^ ^246^ ^]^ REDL hinders the contribution of unspecific tip‐sample interactions and fosters a good correlation between the simultaneously gathered topography and force adhesion maps.^[^ ^247^ ^]^ MRI allows the quantitative visualization of force events in local regions of the scanned features.^[^ ^246^ ^]^ Finally, the unbinding forces (*F*) of complex dissociation are dependent on the loading rate (*r*) of the AFM lever. The kinetic parameters of the studied biological complexes, such as the dissociation rate at zero force (*k*_off_) and the distance between the bound and transition states (*x*_*β*_), can be obtained using the Ritchie–Evans (Equation 4).^[^ ^248^ ^]^

(4) $$F=\frac{k_{B}T}{x_{\beta }}ln\left(\frac{x_{\beta }}{k_{\text{off}}k_{B}T}r\right)$$

where *k*_*B*_ (J/K) and *T* (K) are the Boltzmann constant and the absolute temperature. The complex lifetime *τ* is inversely correlated with dissociation rate parameter by Equation (5).

(5) $$\tau \;=\frac{1}{k_{\text{off}}}\;$$

Advanced phenomenological frameworks are required for the correct interpretation of the force‐extension data distributions obtained at different loading rates.^[^ ^249^ ^]^ Thus, the unbinding forces rely on the semilogarithmic dependency of the loading rate, and the fitting trend indicates the number of intermediate states and associated energy barrier landscapes according to the mechanical dissociation of biology complexes.^[^ ^250^ ^]^ To extract the equilibrium free energies Δ*G* (J) between the two defined thermodynamic states from the distributions of the irreversible work along both connecting trajectories,^[^ ^251^ ^]^ it should be considered in Equation (6).

(6) $$e^{-\Delta G/k_{B}T}=\langle e^{-W_{i}/k_{B}T}\rangle i$$

where *W*_*i*_ (J) is the non‐equilibrium work performed on each experimental trajectory connecting both complex states and *i* refers to the number of different trajectories.

### 5.2 Recognition Imaging and FS in Cancer Research

Single‐molecule force spectroscopy using AFM (AFM‐FS) has transformed our understanding of the molecular connections that drive cancer growth. The information contained in the force curves can indeed be used to investigate the specific interactions between cancer biomarkers and their microenvironment.

AFM‐FS has thus been proposed as successful strategy for cancer detection and surveillance to investigate the mechanical characteristics of circulating tumor cells. As example, an earlier study identified several key nanomechanical biomarkers of single circulating tumor cells for the detection of castration resistant prostate cancer.^[^ ^121^ ^]^ Understanding the dynamic changes in cancer biomarkers required the capacity to investigate these interactions at the nanoscale, thus opening the door for more precise diagnoses. AFM‐FS has been further combined with microfluidics to sort circulating cancer cells and elucidate the forces driving their interaction with the extracellular matrix, by clarifying the delicate mechanics of cancer cell adhesion and providing important insights into prospective targets for interrupting metastatic pathways.^[^ ^252^ ^]^

Cancer metastasis has proved related to binding of integrin proteins with the extracellular matrix, triggering the regulation of cytoskeleton formation and alterations in the interactions between integrin‐ECM.^[^ ^253^ ^]^ Moreover, cancer cells exert traction forces on their neighboring environment which has been identified as a promising hallmark of this disease.^[^ ^254^ ^]^ These traction forces can be affected by mechanical stimuli caused by their surrounding contractile fibroblasts impacting cancer progression.^[^ ^255^ ^]^ AFM‐FS has been employed to demonstrate that endothelium also exhibits different intermolecular force with different types of cancer bladder cells,^[^ ^256^ ^]^ showing that the unbinding force between ICAM‐1 adhesion receptor and J82 bladder cancer cells decreased from *F* = 20–30 pN to *F* = 18 pN, when the receptor ICAM‐1 was blocked; whereas the force values for RT112 remained unaltered in presence of the anti‐ICAM‐1 antibody. Thus, endothelial ICAM‐1 does not affect adhesion interactions with the less invasive RT112 bladder cell line, and the degree of cellular differentiation in cancer could modulated their adhesion properties by modifying protein receptor expression at their external membrane (dysregulation of cellular membrane homeostasis as a crucial modulator of cancer risk). Another aspect to consider is that the strength of adhesion is also influenced by the contact time between both biological systems in contact. The interaction force between the endothelium and MB231 breast cancer cells experienced an increase of nearly 25‐fold times when the contact time shifted from 0.5 s to 300 s.^[^ ^257^ ^]^ This outcome was in line with the fact that after the initial attachment between tumor‐endothelial cells via weak interactions, a time‐dependent process of maturation and stabilization takes place involving the translocation of specific receptors, such as endothelial galectin‐3 or Thomsen–Friedenreich glycoantigen (TF‐Ag), expressed in cancer cells to increase the number of adhesion events among both cellular bodies.^[^ ^109^ ^]^ Similar findings have been achieved for the adhesion of low 4T1‐LM and high FP10SC2 metastatic murine breast cancer cells.^[^ ^118^ ^]^

Moreover, molecular recognition AFM‐FS imaging studies have been conducted to study variations in adhesion properties according to the malignancy level of breast cancer cells. The TREC method was used to study the interactions between AFM tips functionalized with anti‐epidermal growth factor receptor (EGFR) and EGFR localized at the membrane of 435/breast cancer metastasis suppressor‐1 (BRMS1) cells.^[^ ^258^ ^]^ Functionalized AFM tips with the C‐terminal domain of *Perfringolosyn* O toxin (θ‐toxin) displayed stronger interaction forces with cholesterol, and they were thus used as a tool to monitor cholesterol distribution in cellular membranes of cancer cells (Figure 6D,E).^[^ ^259^ ^]^ Similar unbinding forces, in the order of *F* = 120–140 pN, were found for all MCF10A, MCF10AT, and MCF1°CA1a cell lines. Nevertheless, the adhesion maps of the most invasive MCF1°CA1a breast cancer cells gave rise to more events compared to the less malignant MCF10AT cells and the benign epithelial MCF10A cells.^[^ ^75^ ^]^ Thus, these results showed an increase in the tip‐sample binding probability, where cholesterol was densely distributed. Specifically, the cholesterol‐enriched membranes of malignant MCF1°CA1a yielded circa 2 and 5‐fold times larger binding probability compared with MCF10AT and MCF10A cells, respectively (Figure 6F). Thus, the cholesterol content of cancer cell membranes, which is directly linked to their grade of malignancy, proved to modulate the interaction with *θ*‐toxin probes which could serve as a prognostic methodology in cancer research.

Finally, the actions of drugs and therapeutic agents were evaluated using AFM‐FS experiments. This is the case of specific intermolecular interactions between T cells and lung cancer cells expressing programmed death ligand 1 (PDL1).^[^ ^260^ ^]^ The PDL1 biomarker appears in early stage resected lung cancer cells.^[^ ^261^ ^]^ The presence of nivolumab in the media increased the adhesion force of this biological system of tens to hundreds of pN, as evaluated at different timescales. This observation agreed with clinical evidence of nivolumab action in the regression of lung cancer and the recovery of cellular properties.^[^ ^262^ ^]^

### 5.3 FS and Recognition Imaging in Neurodegeneration Research

AFM‐FS has been successfully applied to study protein–protein interactions in the context of amyloid formation and neurodegeneration, to unravel molecular adhesion forces, as well as interaction of protein self‐assemblies with external disease related factors.

**Figure 7:**
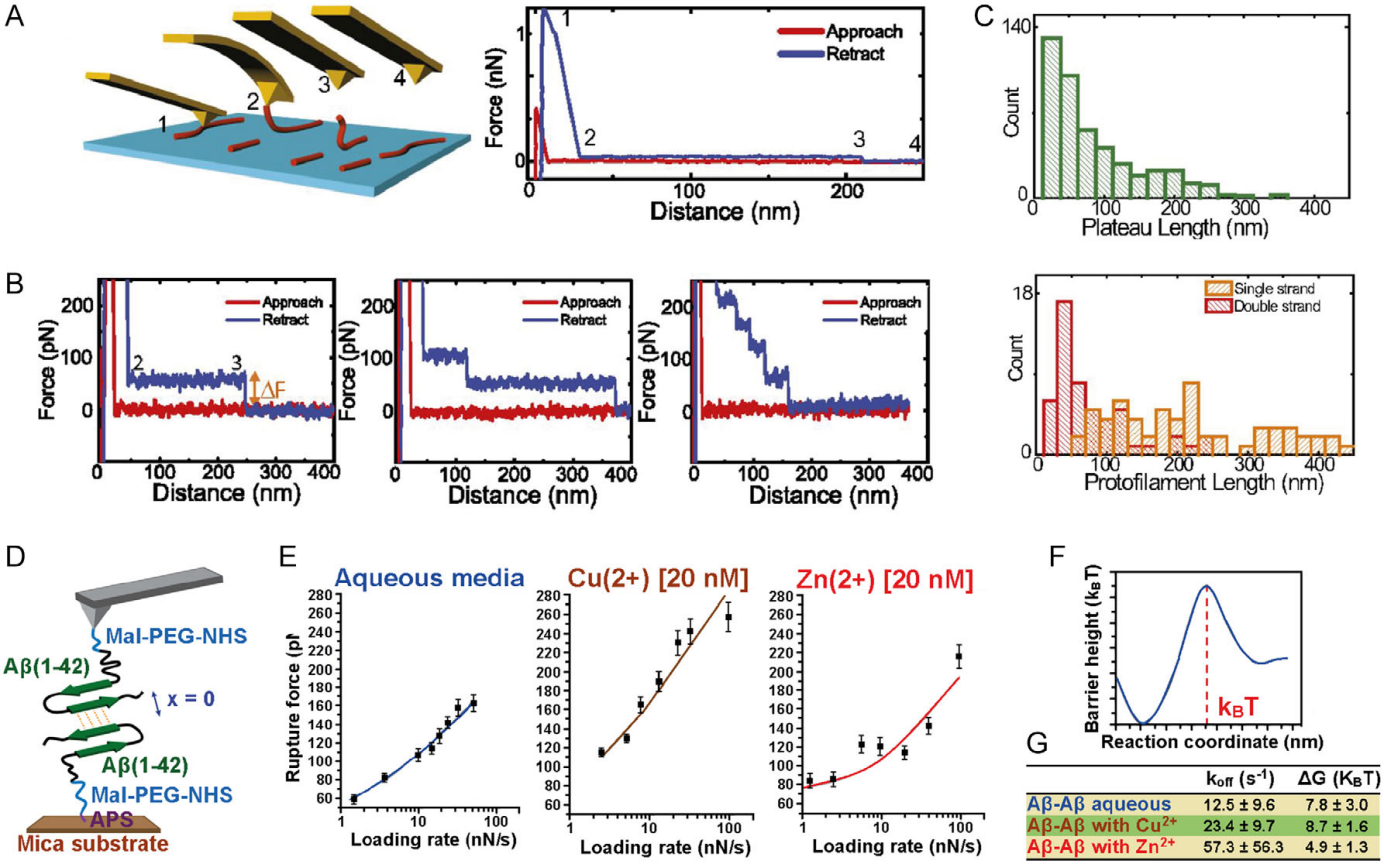
Force spectroscopy of amyloidogenic protein and oligomeric assemblies. A) Model of the pulling of single protofilaments and the relative force–distance curve, adapted and printed with permission from,^[^ ^89^ ^]^ Copyright 2018, Proceedings of the National Academy of Sciences. 1) AFM tip is pressed against a protofilament. 2) The pressure causes polymer breaking and a constant force plateau due to its unzipping from the protofilament surface. 3) Tip–filament contact is lost during the pulling. 4) Tip return to the initial position. B) Single force plateau due to pulling of different numbers of protofilaments. C) Distribution of constant‐force pulling events (plateaus) and protofilament lengths. D) Illustration of the chemical strategy devoted to covalently attach Aβ_42_ fibrils on the AFM tip and mica surface, respectively. E) Unbinding force versus loading rate plots for the dissociation of Aβ_42_ dimers in aqueous solution and under the presence of Cu^2+^ and Zn^2+^ ions. F) Representation of energy profile landscape diagram indicating the energy barrier height corresponding to the associated and dissociated complex states. G) Kinetic and thermodynamic parameters found for Aβ_42_ dimers in aqueous solution and under the presence of Cu^2+^ and Zn^2+^ ions, respectively. k_off_ and ΔG are the dissociation rate at zero force and the free‐energy barrier between the bound and unbound states. D‐G printed with permission from,^[^ ^263^ ^]^ Copyright 2016, PLOS One.

As discussed in Section 3.2, single‐strand protofilaments could be identified as the elementary unit of amyloid fibrils formed by the α‐synuclein protein involved in the onset of PD.^[^ ^89^ ^]^ However, because of their Ångström‐size height, it could not be possible to investigate their inner structural properties by conventional nano‐indentation methods. Thus, AFM‐FS could be used to manipulate, stretch, and characterize the properties of amyloids, to investigate their inner structural, thermodynamic, and mechanical properties (**Figure** **7**A).^[^ ^89^ ^]^ The analysis of the force curves showed that one or multiple protofilaments could be unzipped from the surface (Figure 7B), with typical lengths in the order of the protofilaments length (Figure 7C), allowing to determine the energy of interaction with the surface. This approach, combined with WLC analysis of stretching events occurring during the pulling of the protofilaments, allowed to measure an average internal persistence length of 0.3 nm, which was consistent with the elastic properties of a polymer chain composed of unstructured monomers of α‐synuclein. Thus, AFM‐FS could prove that protofilaments were composed by a chain of unordered monomers, and were less far structured than mature amyloid fibrils.

In misfolded protein‐related diseases, the presence of certain divalent cations could promote higher adhesion forces between the Aβ peptides. These peptides could be attached to the AFM tip and substrate surface (Figure 7D); exploiting the reactivity of the hydroxysuccinimide moiety of the linker molecules with the primary amines coming from the amyloid peptide. The interactions between Aβ_42_ peptides increased from 110 pN to 120 pN‐170 pN in presence of Zn^2+^ and Cu^2+^ ions^[^ ^263^ ^]^ (Figure 7E). It was thus hypothesized that the presence of Zn^2+^ in the media ameliorates the intermolecular forces between Aβ_42_ peptides, because its association is almost purely diffusion‐controlled. The dissociation kinetics were also favored in presence of these ions with an increase of *k*_off_ of circa 2–5‐fold in present of Zn^2+^ and Cu^2+^ ions, compared with Aβ_42_ alone. Analyzing the energy landscapes (Figure 7F), the equilibrium free energy in presence of Cu^2+^ was similar to the values obtained for Aβ_42_ alone, and in the order of 8 k_B_T, but, it was almost twofold reduced with zinc ions to circa 5 k_B_T. These results suggested that Cu^2+^ and Zn^2+^ concentrations could alter the nucleation and elongation phases of amyloid fibrils formation during protein aggregation.

AFM‐FS studies were further applied to study how substitutions in peptide sequence could affect the stability of amyloid peptides. VPV peptide substitutions in the C‐terminal region showed to stabilize the Val36‐Gly37 turn in dimeric Aβ_42_ species, reducing their *k*_off_ from 1.6 ± 0.3 s^−1^ to 5.7 ± 0.3 s^−1^ for native Aβ_42_. Even if their unbinding force interactions remained similar at the tested conditions, and in the order of circa 70 pN,^[^ ^264^ ^]^ this stabilization effect was more evident for Aβ_40_ where the VPV mutation lead nearly 4.5‐fold greater stabilization than the wild‐type amyloid dimeric entities. The two extra amino acids in Aβ_42_ peptides were responsible thus for higher propensity of aggregation than Aβ_40_, based on the C‐terminal length‐dependence of oligomerization mechanisms.^[^ ^265^ ^]^ Similar studies considered pH as an additional factor that could affect intermolecular interactions of amyloid proteins.^[^ ^266^ ^]^ Weak forces, including hydrophobic interactions and aromatic stacking, have been proposed to be the driving factors in the anti‐amyloidogenic response of polyphenols, with potential inhibitory activity in the amyloid fibrillation process of Aβ peptides.^[^ ^267^ ^]^

## 6 AFM Nanoindentation Mechanical Analysis

In this section, we discuss the role of the mechanical properties in the context of cancer and neurodegenerative diseases. Nanomechanical signature of cancer cells and amyloid fibrils pave the way for monitoring disease progression and evaluating the efficiency of bespoke drug delivery treatments and for the understanding of the molecular processes involved in disease onset and progression. Nanoindentation offers the possibility to interrogate mechanical properties from the single‐molecule level to the subcellular and cellular levels.^[^ ^59^, ^72^ ^]^ Notwithstanding the fundamental advances achieved, we also describe here how nanoscale dimensions of biomolecules, cellular structural hierarchy, degree of entanglement of cytoskeleton components, and chemical heterogeneity, along with the inherent uncertainties of theoretical models and nano‐indenter selection, still require following strict protocols and propose new strategies to accurately determine the mechanical cues of the tested biological systems at multiple timescales.

### 6.1 Principles

**Figure 8:**
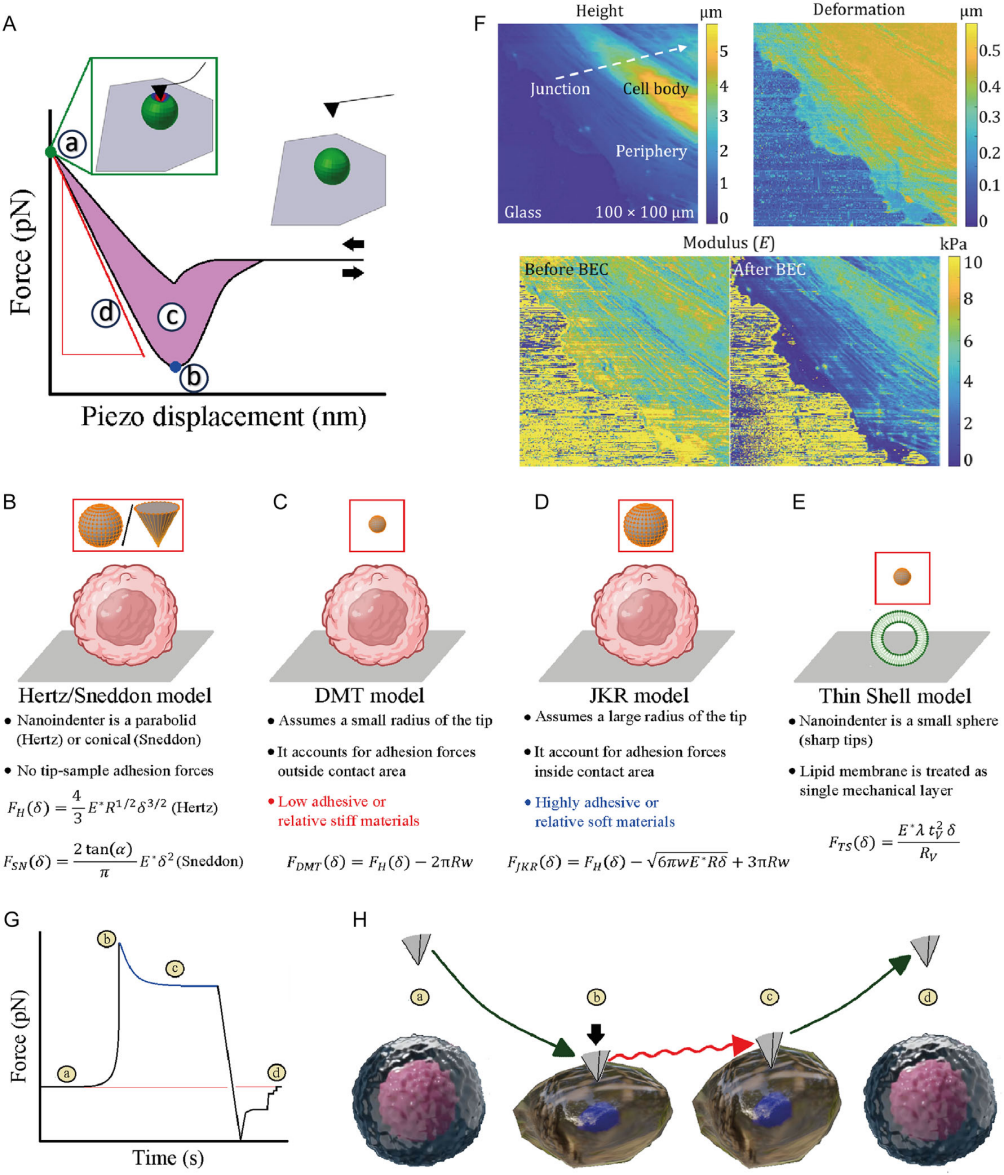
Nanomechanical imaging models for measuring the stiffness of cancer cells. A) Force‐distance curve profile (left black arrow: approach, right black arrow: retraction) where the a) tip‐sample contact point, b,c) the maximum unbinding force and deformation energy, and d) the slope of the contact region required to extract the Young's modulus are detailed. Printed with permission from,^[^ ^379^ ^]^ Copyright 2023, Multidisciplinary Digital Publishing Institute. B–E) Relevant mechanical indentation models used for determining Young's modulus in solid bodies (e.g., tissues, cells, amyloidogenic proteins, or rigid drug carriers) and hollow lipid vesicles. B) The Hertz/Sneddon model. C) DMT model. D) JKR model. E) Thin‐shell model. Images were created using BioRender.com. F) Topography, deformation, and Young's modulus map (DMT model) before and after bottom effect correction (BEC) of endothelial cells. Reprinted with permission from,^[^ ^380^ ^]^ Copyright 2020, Cell Press. G) Representative force‐time curve profile where the AFM tip is approaching a) the sample surface, b) the tip reaches the sample contact point, c) the sample reacts to the external stress leading to a force relaxation, and d) the AFM tip moves away the sample surface. The viscosity can be extracted from this force‐time curve through the Equation (14).^[^ ^293^ ^]^ H) Schematic representation of how a force‐time measurement is carried out in a single cell (a,b: tip approach to the cell surface, b,c: stress‐relaxation measurements, c,d: tip retraction from the cell surface). Green and red arrows indicate the AFM tip vertical movement and the cell response under the external load force, respectively.

In nanoindentation, the AFM tip apex works similarly as a nanoindenter by applying a controlled force and causing local deformation at the most external surface of a sample (**Figure** **8**A).^[^ ^59^ ^]^ Continuous force‐distance curves could be so recorded by indenting the sample (points a–e). Relevant information can be obtained from these curves, such as the: peak force (Figure 8A, point a), maximum tip‐sample unbinding force (Figure 8A, point b), energy dissipation (Figure 8A, point c), apparent Young's modulus of the sample extracted from the slope of the contact region (Figure 8A, point d), and sample deformation (Figure 8A, point e).

Accurate AFM tip characterization is needed before performing nanoindentation measurements to prevent the acquisition of unreliable data. As introduced at paragraph 5, the AFM cantilever is calibrated via thermal noise and Sader's calibration methods, which typically render an inherent error of nearly 25%–30% based on the assumptions made concerning the lever geometry and negligence of the damping effects, especially in the measurements carried out in liquid media to measure biological samples.^[^ ^268^ ^]^ Significant efforts have been devoted to overcoming these limitations. It was recently proposed that a standardized nanomechanical atomic force microscopy procedure (SNAP), characterizing the spring constant of the AFM lever using vibrometry, minimizes the associated error to 1%.^[^ ^269^ ^]^ Next steps are the calibration of the AFM tip radius and selection of the mechanical model (Figure 8B–E).^[^ ^59^, ^145^ ^]^ Currently, there are commercially available AFM probes with many different tip geometries, which evidences the need to develop a subsequent number of theoretical model frameworks. The final model selected to ascertain the elasticity of the sample directly relies on the aforementioned tip geometry, but also on the nature of the tested sample (i.e., solid bodies vs. hollow structures). For instance, for tips with four‐sided regular pyramidal geometries, it is challenging to accurately determine the parameters of the indenter‐sample surface contact area, tip radius estimation, and the displayed non‐linear elastic responses during the indentation measurements.^[^ ^270^ ^]^ Therefore, it is advisable to employ nanoindenters with other geometries, such as paraboloid‐spherical or conical.

The most commonly used mechanical models in nanobiomedicine are here discussed (Table 2, Supporting Information). The Hertz model was initially derived to describe a purely elastic deformation of two perfectly smoothed spheres in contact via a linear strain‐stress relationship, but it was later extended to consider a sphere or a paraboloid indenting a sample that is assumed as an elastic half space, i.e., a sphere of R→ ∞.^[^ ^271^ ^]^ In turn, an elastic half space can be considered a homogeneous and isotropic material. While biological materials can be approximated only in some cases and under specific constraints to an elastic half space, Hertz model and the ones derived from it are the most widely applied to study the mechanical properties of biological systems. The apparent Young's modulus E* (Pa) of the sample can be determined knowing the applied force *F* (N) by the nanoindenter AFM tip as follows (Equation 7).^[^ ^271^ ^]^

(7) $$\;F_{H}\left(\delta \right)=\frac{4}{3}E^{*}R^{1/2}\delta^{3/2}$$

where *δ* (nm) refers to the indentation depth of the AFM tip apex and *R* (nm) is the radius of curvature of the AFM tip. Equation (7) holds for a spherical indenter only if the indentation depth is small compared to the tip radius (δ≪R). The Hertzian model was further extended to account for different tip geometries, such as in the case of the Sneddon model that considers a conical shape for the AFM tip shape^[^ ^271^, ^272^ ^]^ (Equation 8).

(8) $$F_{\text{Sn}}\left(\delta \right)=\frac{2\tan\alpha }{\pi }E^{*}\delta^{2}$$

where *α* (°) denotes the half‐angle of the conical tip. However, these models do not consider the adhesion and frictional forces between the contacting surfaces of both rigid bodies and can only apply a maximum strain of 10% to avoid significant deviations arising from comparisons with bulk experimental measurements. Furthermore, mapping AFM experiments, where the topography and mechanical maps are simultaneously recorded to compare both signals in certain pixel images, require the use of sharp tips to prevent convolution artifact broadening effects,^[^ ^273^ ^]^ causing loss of lateral resolution. In this case, long‐range van der Waals interactions are sufficiently large to render the Hertzian models unsuitable. Derjaguin–Müller–Toporov (DMT)^[^ ^274^ ^]^ and Johnson–Kendall–Roberts (JKR)^[^ ^275^ ^]^ models were developed to extend the Hertz model accounting for adhesive interactions, which are relevant when studying soft biological samples, and presented in Equation (9) and (10), respectively.

(9) $$F_{\text{DMT}}\left(\delta \right)=F_{H}\left(\delta \right)-2\pi Rw$$

(10) $$F_{\text{JKR}}\left(\delta \right)=F_{H}\left(\delta \right)-\sqrt{6\pi wE^{*}R\delta }+3\pi Rw$$

where *w* is the work of adhesion (J m^−2^), R is the radius of the probe, and a (nm) is the contact radius, respectively. The DMT model is mostly applicable in the case of low adhesive or stiff materials, since it considers adhesion only outside the contact area and may have difficulties in determining the mechanical properties of some soft materials because of the incomplete treatment of the attractive interaction forces exerted by the contact region between the AFM tip and sample surface.^[^ ^276^ ^]^ Instead, the JKR model considers the adhesion inside the contact area and is generally the most suitable approach to study mechanical properties of highly adhesive or very soft materials.^[^ ^277^ ^]^ Yet, all the aforementioned models assume that the indented material is homogeneous and has isotropic mechanical properties. Finally, the thin‐shell model^[^ ^278^ ^]^ is valid only for hollow particles with no interior cargo. This model is suitable when the shell radius is significantly larger than the shell thickness, as in the case of lipid membranes. The bilayer membrane was also considered as a single layer and the Young's modulus could be measured via Equation (11).

(11) $$F_{\text{shell}}\left(\delta \right)=\frac{\lambda \;t_{V}^{2}}{R_{V}}\;E^{*}\delta$$

where *R*_V_ and *t*_V_ are the radius (nm) and thickness (nm) of the nanoindented samples, respectively, and *λ* is a geometry‐dependent proportionality factor. The thin‐shell model was previously employed to determine the elastic modulus of other biological systems, including viruses such as SARS‐CoV‐2^[^ ^279^ ^]^ or pollen grains.^[^ ^280^ ^]^

Once the most suitable mechanical model has been chosen, the apparent Young's modulus E* could be derived from the local nanoindentation measurements.^[^ ^59^ ^]^ However, the measured apparent Young's modulus E* can differ from the intrinsic Young's modulus *E* (Pa) since it reflects the combined effects of material properties, geometry, and experimental conditions and accounts for both the combined elastic properties of the tip and the sample. Therefore, the apparent Young's modulus must be corrected using the Poisson's ratio (*υ*) of the material under investigation via Equation (12).^[^ ^59^ ^]^

(12) $$\frac{1}{E^{*}}=\frac{1-\upsilon^{2}}{E}+\frac{1-\upsilon_{t}^{2}}{E_{t}}\approx \frac{1-\upsilon^{2}}{E}$$

*E*_t_ and *υ*_t_ refer to the elastic modulus (Pa) and Poisson's ratio of the AFM tip, respectively. The AFM tips are typically made by stiff materials, such silicon nitride (Si_3_N_4_) with a Young's modulus in the order of hundreds of GPa, thus making the AFM tip contribution almost negligible when measuring soft biological samples. Currently, there are no homogeneous criteria for the optimal *υ* value to be selected in soft biology samples, where *υ* typically tends to 0.5. Nevertheless, *υ* ranges from 0.25 to 0.50 in biological samples (Table 3, Supporting Information). For example, samples with the same nature originating from human lungs strongly differ in *υ* values (0.25, 0.34, 0.40, and 0.50 for lung cancer cells, parenchyma tissue, and lung tissue, respectively). Similar findings were observed in cancer cells from other tissues. Differences of 0.2 in *υ* can lead to errors of up to 100% in 3D systems for the distribution and magnitude of all constructed traction components.^[^ ^281^ ^]^ Recently, efforts have been devoted to precisely determine *υ*, such as the digital image correction (DIC) evaluating the strain between two marked edges by computing the average strain.^[^ ^282^ ^]^ This approach is capable of reducing the difference in *υ* for soft samples with the same nature but requires conscious calibration of the inner lens setup. The development of computer simulation frameworks and numerical analysis toolboxes has been shown to be a promising strategy for obtaining reliable cellular *υ* values.^[^ ^281^ ^]^

During the indentation of soft matter samples like small biological molecules and living cells (Figure 8F), a further bottleneck to determine reliably mechanical properties has been due to the contribution to the measurements of the properties of stiff substrates; known as “bottom effect”, which causes an overestimation of the tested Young's modulus.^[^ ^283^ ^]^ Usually, living cells and amyloidogenic proteins are attached to mica, HOPG, ZnS, ZnSe, or glass surfaces with Young's modulus typically in the range between 10 and 100 GPa.^[^ ^284^, ^285^ ^]^ Bottom effect correction (BEC) was formulated to subtract the mechanical background of rigid surface materials by considering the geometry of the tip.^[^ ^286^ ^]^ BEC can be applied pixel‐by‐pixel to the mapping images, rendering neat boundaries between the stiff surface and biological features. The BEC mathematical expressions for the paraboloid semi‐spherical Equation (13) and conical (14) shapes are as follows.

(13) $$F_{\text{BEC}}=F[\frac{1}{h^{0}}+\frac{1.133\sqrt{\delta R}}{h}+\frac{1.497\delta R}{h^{2}}+\frac{1.469\delta R\sqrt{\delta R}}{h^{3}}+\frac{0.755\left(\delta^{2}R^{2}\right)}{h^{4}}]$$

(14) $$F_{\text{BEC}}=F[\frac{1}{h^{0}}+\frac{0.721\delta \tan\alpha }{h}+\frac{0.650\delta^{2}{\text{tan}}^{2}\alpha }{h^{2}}+\frac{0.491\delta^{3}{\text{tan}}^{3}\alpha }{h^{3}}+\frac{0.225\delta^{4}{\text{tan}}^{4}\alpha }{h^{4}}]$$

where *F*_BEC_ (N) is the bottom effect correction force and *h* (nm) is the thickness of the indented material. BEC terms are a function of the relation between *h* and the contact radius ($\sqrt{\delta \;R}$). It has been shown that substrate rigidity can alter the cell mechanosensing response by affecting the organization of the actomyosin cytoskeleton.^[^ ^287^ ^]^ Therefore, it is mandatory to acquire knowledge regarding the substrate contribution and reducing it using BEC calculations. Alternatively, the bottom effect is almost negligible when cells or amyloidogenic proteins are immobilized on soft substrates, such as polyacrylamide gels, or with rigidity properties similar to those of the tested biological samples.^[^ ^288^ ^]^

Overall, as summarized in Table 4, Supporting Information, nanoindentation via AFM has been extensively applied to unravel the mechanical properties of amyloidogenic proteins, tissues, and cells associated with neurodegeneration and cancer malignancies. Measurements of Young's modulus values obtained from nanoindentation depend fundamentally also on the chosen environmental conditions. Air measurements indeed showed sometimes larger Young's modulus than those obtained in liquid media. This effect is caused by the softening effect of water uptake into the sample, which refers to the phenomenon in which biological features undergo a decrease in mechanical stiffness based on the loss of their inner hierarchical architectural junctions.^[^ ^234^ ^]^ Scanning conditions affect amyloid proteins of different natures. The *E* value of α‐synuclein fibrils remained similar under air and liquid conditions,^[^ ^165^ ^]^ whereas a decrease of almost two orders of magnitude was observed for Aβ_1‐40_, which likely depend on the maturity of the amyloid fibrils studied.^[^ ^289^ ^]^ Furthermore, the formation of a water film on the scanned biological features dramatically affects AFM measurements in air, leading to an increase in mechanical data dispersion.^[^ ^290^ ^]^ Thus, the best strategy is to determine the *E* value of carcinogenic cells or soft biomolecules in liquid environments mimicking the relevant physiological conditions. Other pivotal factors to optimize nanomechanical measurements are the employed load force and indentation depth parameters, respectively. A larger Young's modulus was observed when the load force and indentation depth increased. Sample elasticity monitoring requires homogenization of experimental conditions to better compare different reported research studies.

Finally, other mechanical properties, such as viscosity coefficients, can be obtained by bimodal AFM (Section 3 *, AFM imaging*). This operational mode was used to study how peptide sequence and interaction with the substrate can affect amyloid fibril viscoelastic properties.^[^ ^291^ ^]^ This technology was also employed to map the viscoelastic properties of cells with high lateral resolution.^[^ ^161^ ^]^ Many models have been built to describe the viscoelastic properties of tested soft matter samples using force‐distance curves.^[^ ^292^, ^293^ ^]^ A frequently used approach is the Maxwell model, which considers the material of interest consists of an elastic spring plugged into series of viscous dashpots.^[^ ^294^ ^]^ The Maxwell model is valid to ascertain the viscoelastic properties of soft materials, such as cells (Figure 8H).^[^ ^295^ ^]^ This model assumes that a force response *F* to a constant deformation event decays exponentially over time while being recovered to zero (Equation 15).

(15) $$F=A\;exp\left(\frac{-\left(t-t_{0}\right)}{\tau }\right)$$

where *A* is the relaxation force amplitude (N) and τ the relaxation time (s), respectively. The viscosity *μ* (Pa·s) and *E* (Pa) are in turn related by *τ* (Equation 16).^[^ ^293^ ^]^

(16) $$\tau =\frac{\mu }{E}$$

Thus, the viscosity μ can be extracted from force‐time curves (Figure 8G). It is possible to record maps of the scanned area of interest by stress relaxation microscopy, which generates force relaxation curves at each scanned pixel region. Viscoelastic bodies also treasure the ability to store energy allowing the elastic and viscous components to be decoupled depending on their response under dynamic vibrational sources with frequency *ω* (rad/s). The complex Young's modulus (G*, Pa) of the examined sample is composed of the storage modulus (*G′*, Pa) as real term and of the loss modulus (*G″*, Pa) as the imaginary part, as defined in Equation (17).^[^ ^296^ ^]^

(17) $$G^{*}\left(\omega \right)=G\prime \left(\omega \right)+iG\text{″}\left(\omega \right)$$

where *ω* is the measurement frequency (rad/s). *G′(ω)* accounts for the elastic deformation, whereas *G″(ω)* accounts for the viscous counterpart undergone by the oscillatory cantilever. Working with successive cantilever harmonics, it can be demonstrated that Equation (11) can be converted into Equation (18).^[^ ^297^ ^]^

(18) $$E^{*}=\frac{E}{\left(1-\upsilon^{2}\right)}=\frac{G\prime \left(\omega \right)}{\left(1-\upsilon^{2}\right)}+i\frac{G\prime \prime \left(\omega \right)}{\left(1-\upsilon^{2}\right)}$$

This assumption is valid since elastic modulus of the AFM tip is nearly one magnitude order larger compared to soft living cell membranes and other soft matter systems. Thus, G′ and G″ represent the linear sample viscoelasticity assessed at an average indentation depth (*δ*_*0*_, nm) as represented in Equation (19) and (20), respectively.
(19)

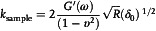



(20)

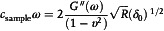

where $k_{\text{sample}}$ is the effective stiffness and *c*_sample_ is the damping (Ns m^−1^) of the sample at a fixed $\delta_{0}$ indentation value. Finally, the loss tangent can be calculated as the ratio of the loss and storage moduli (Equation 21).

(21) $$\text{tan}\left(\delta \right)=\frac{G\prime \prime }{G\prime }$$

tan *(δ)* measures the extension of energy lost caused from the viscous nature of the examined sample. The importance to address tan *(δ)* in cellular systems relies on the fact that the damping factor of extracellular viscosity promotes cell migration and cancer proliferation.^[^ ^104^, ^298^ ^]^ Thus, loss tangent conveys the intrinsic fluidity properties of the studied samples. For example, cancer cells exhibit more fluid‐like compared to the benign counterparts.^[^ ^299^ ^]^

### 6.2 Nanomechanical Properties of Cancer Cells

Cancer cells typically show nanomechanical characteristics different from those of healthy cells. AFM studies have examined the stiffness of several kind of cancer cells revealing not only a variation of their stiffness but also spatial changes within the same tumor, underscoring the heterogeneity that is frequently a characteristic of cancer, including the cases of ovarian, breast, bladder, prostate.^[^ ^75^, ^77^, ^300^ ^]^ This nanomechanical variability inside cancer cells could achieve far‐reaching consequences for understanding metastatic potential and treatment responses. Nanomechanical measurements, delivering high‐resolution images combined with mechanical information, have allowed researchers to detect differences that may influence cancer growth and response to treatment.^[^ ^75^, ^77^, ^300^ ^]^

**Figure 9:**
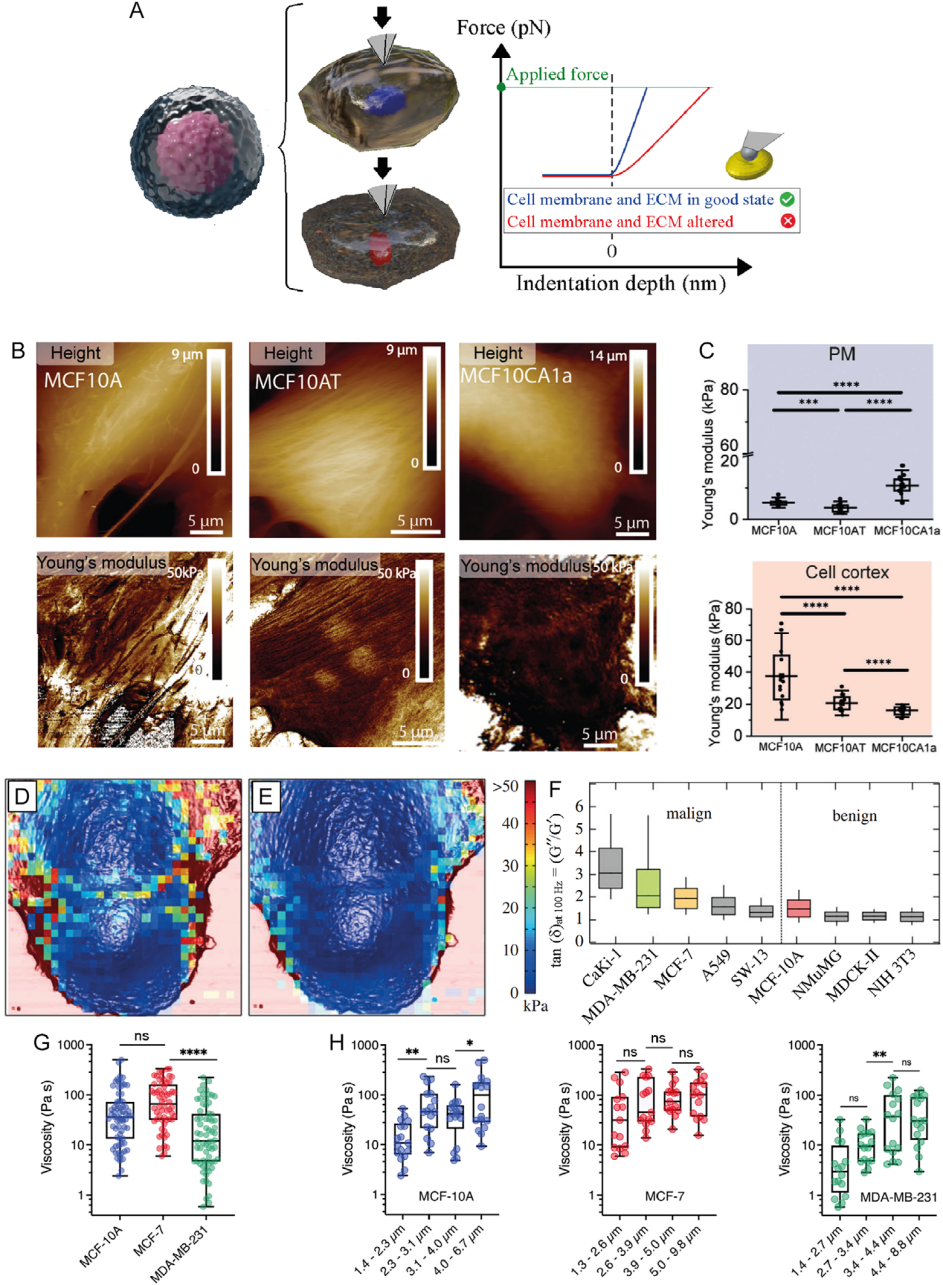
Nanomechanical differentiation of healthy versus cancer cells. A) Schematic representation of a living cell. Healthy cells (blue line) with unaltered membranes and structured ECM usually render greater mechanical properties compared to cancer cells (red line). B) From the left to the right: topography and Young's modulus map (Hertz model) of healthy (MCF10A), premalignant (MCF10AT) and cancer (MCF1°CA1a) breast cells. The vertical scale bars of the Young's modulus maps are settled at 50 kPa to better compare the differences among the tested conditions. C) Box plots with the gathered Young's modulus values of the plasma membrane and the cell cortex for the three examined cell lines. Printed with permission from,^[^ ^75^ ^]^ Copyright 2020, Wiley. D) Overlay of the topography map of a benign NMuMG breast epithelial cell with the force data map to estimate the storage modulus (G′). E) Merge of the topography and force data maps to ascertain the loss modulus (G″) of NMuMG cells. F) Loss tangent (G″/G′) of breast cancer cells with different metastatic potential (oscillation frequency of 100 Hz). Reprinted with permission from,^[^ ^299^ ^]^ Copyright 2014, The Royal Society. G) Boxplot of the viscosity parameter found in MCF‐10 A, MCF‐7 and MDA‐MB‐231 breast cancer cells (in blue, red and green colors, respectively) obtained by MRS. H) Viscosity distributions according to the length of the examined breast cancer cell lines. Printed with permission from,^[^ ^306^ ^]^ Copyright 2024, The Royal Society of Chemistry.

Nanomechanical studies should be first individually devoted to each type of cancer due to their inherent high level of heterogeneity. As example, it was reported that the Young's modulus *E* of different cancer types differs up to fivefold within the same tissue for meningothelial meningioma, fibrous meningioma, glioblastoma, and metastatic adenocarcinoma.^[^ ^301^ ^]^ Different cell lines also significantly varied values of stiffness, even if they would come from the same tissue origin, such as Calu6 and A549 cancerous lung cells exhibiting *E* = 33 Pa and *E* = 1225 Pa, respectively.^[^ ^300^ ^]^ Significant differences have been shown also for the same cellular line at specific cancer stages, such as in the case of ovarian tumor and highly invasive ovarian cancer cells with Young's modules varying from kPa to MPa.^[^ ^122^ ^]^ Besides these differences, the general observed trend observed is that healthy cells from a certain tissue have higher Young's modulus *E* compared with their carcinogenic counterparts (**Figure** **9**A).^[^ ^117^ ^]^

Cellular stiffness *E* is further directly influenced by the status of the extracellular matrix and cellular membrane state, which can synergistically regulate cytoskeleton organization.^[^ ^302^ ^]^ Indeed, softening of cancer cells starts with disruption of the cytoskeleton network filaments and actin‐binding proteins,^[^ ^303^ ^]^ which are linked through the action of plectin proteins.^[^ ^304^ ^]^ By conducting nanoindentation experiments at different values of deformation *δ* (nm), it was possible to unravel this complexity in cellular response, differentiating the mechanical performance of the cell membrane and the cell cortex. The contributions of the cellular membranes and cell cortex could be interrogated at *δ* < 50 nm and *δ* > 50 nm, respectively.^[^ ^75^ ^]^ The elastic moduli of three breast cells, benign‐MCF10A, premalignant noninvasive‐MCF10AT, malignant, and invasive‐MCF1°CA1a were determined by fitting the force‐distance curves at the two aforementioned indentation depth regions (Figure 9B,C). Malign breast cancer cells showed nearly threefold larger *E* than the benign and premalignant breast cells with *E* = 4–10 kPa for the cellular plasma membrane region and *E* = 15–20 kPa when the cell cortex was interrogated. To unravel the complexity of the mechanical response of different structural components of a cell, it is also critical the choice of the surface geometry of the nanoindenter that may affect the measured mechanical properties.^[^ ^305^ ^]^ AFM sharp tips have been proven ideal for measuring the Young's modulus *E* of cortical regions that are insensitive to cell thickness, whereas rounded tips (diameters of 1.5−10 μm) were adequate to determine both *E* of cortical/intracellular and intracellular regions with relative insensitivity and sensitivity to cell thickness, respectively.

Nanoindentation has accordingly been leveraged to study the viscoelastic behavior of breast cancer cells. Malignant MCF7 cells have shown a time dependence of the force decay with a double‐exponential behavior independently of the tested load (from 0.5 to 4.0 nN).^[^ ^293^ ^]^ This result suggested that MCF7 cells relax via two separate processes involving cytoskeletal rearrangements and the cellular membrane. In other studies, viscoelasticity measurements by AFM were used to assess breast cancer cells with different metastatic potential.^[^ ^299^ ^]^ The force maps data were used to estimate the storage modulus (G′, Figure 9D) and loss modulus (G″, Figure 9E). Then, the calculated loss tangent (G″/G′) was generally higher in the case of breast cell lines with highly metastatic potential compared to benign breast epithelial cells (Figure 9F). For example, CaKi‐I and MDA‐MB‐23‐I malignant cells had fourfold and twofold times, respectively, larger loss tangent compared to benign NMuMG cystadenoma cells. Complementary magnetic rotational spectroscopy (MRS) confirmed that breast cancer cells with different metastatic potential states differ in their viscosity.^[^ ^306^ ^]^ MCF‐7 and MCF‐10 breast cancer cells had >4 times‐fold greater than MDA‐MB‐231 cells (Figure 9G). The cellular dimensions also impacted their viscosity revealing a positive trend as a function of the cell size for all examined breast cancer cell lines (Figure 9H). Thus, the force‐time curve profile could in principle act as diagnostic tool to discriminate among benign and malign breast tumor cells.

Furthermore, nanomechanical studies have been leveraged to study the partial recovery of the stiffness *E* of cancer cells when they are exposed to drugs, which could indicate reconstruction of the inner cellular machinery to reconstitute the architecture of the plasma membrane and cytoskeleton. Ovarian cells are an illustrative example of this phenomenon. The stiffness of health ovarian cells decayed from *E* = 2.5 ± 2.0 kPa to lower values when converted into carcinogenic (*E* = 1.1 ± 0.9 kPa) and highly invasive cancer cells (*E* = 0.5 ± 0.2 kPa).^[^ ^122^ ^]^ Instead, the supply of docetaxel restored the mechanical properties of the ovarian cells (*E* *=* 4.7 ± 0.2 kPa),^[^ ^77^ ^]^ via stabilization of microtubules and promoting tubulin polymerization.^[^ ^307^ ^]^ Similarly, a combination of nanoindentation measurements with artificial neuronal networks showed longer relaxation times for MCF‐7 cells treated with anticarcinogenic drug agents. These results highlighted how estrogen receptor may modulate the viscoelastic properties of breast cancer cells.^[^ ^308^ ^]^ The treatment with resveratrol led to viscosities almost threefold times larger than the negative control (187 ± 14 Pa·s vs. 56 ± 3 Pa·s, respectively); whereas the action of estrogen had the opposite effect (27 ± 1 Pa·s) inducing membrane fluidization. This methodological approach could be also exploited for other drugs, such as 5‐fluorouracil, doxorubicin, and paclitaxel to evaluate the alteration of the viscoelastic properties in cancers.

Finally, the mechanical properties of customized drug delivery therapy agents against cancer cells were ascertained by nanoindentation measurements. Cellular internalization processes rely directly on membrane deformation when interacting with external agents. For example, engineered liposomes favor fusion with HeLa lipid membranes and the subsequent release of their inner cargo.^[^ ^309^ ^]^ The recognition process between the cellular filipodia and the external agent is dependent not only on chemical recognition but also on the mechanical signature of both bodies.^[^ ^309^ ^]^ Thus, rational design of drug carriers based on both chemical and mechanical properties is essential. For example, exosome size strongly affects elasticity by nearly 3.5‐fold times, when the diameter moves from less than 30 nm to a range of 50–170 nm.^[^ ^310^ ^]^ The mechanical properties of lipid layers were also altered by temperature^[^ ^311^ ^]^ or liposome composition, which is directly linked to the liquid‐solid phase states and subsequently to their bending rigidity.^[^ ^312^ ^]^ The superficial stiffness gold nanoparticles (NPs) have been showed to decrease when coated with bovine serum albumin,^[^ ^313^ ^]^ polyethylene glycol,^[^ ^314^ ^]^ and streptavidin.^[^ ^313^ ^]^

### 6.3 Nanomechanical Properties of Protein Self‐Assemblies in Neurodegeneration

**Figure 10:**
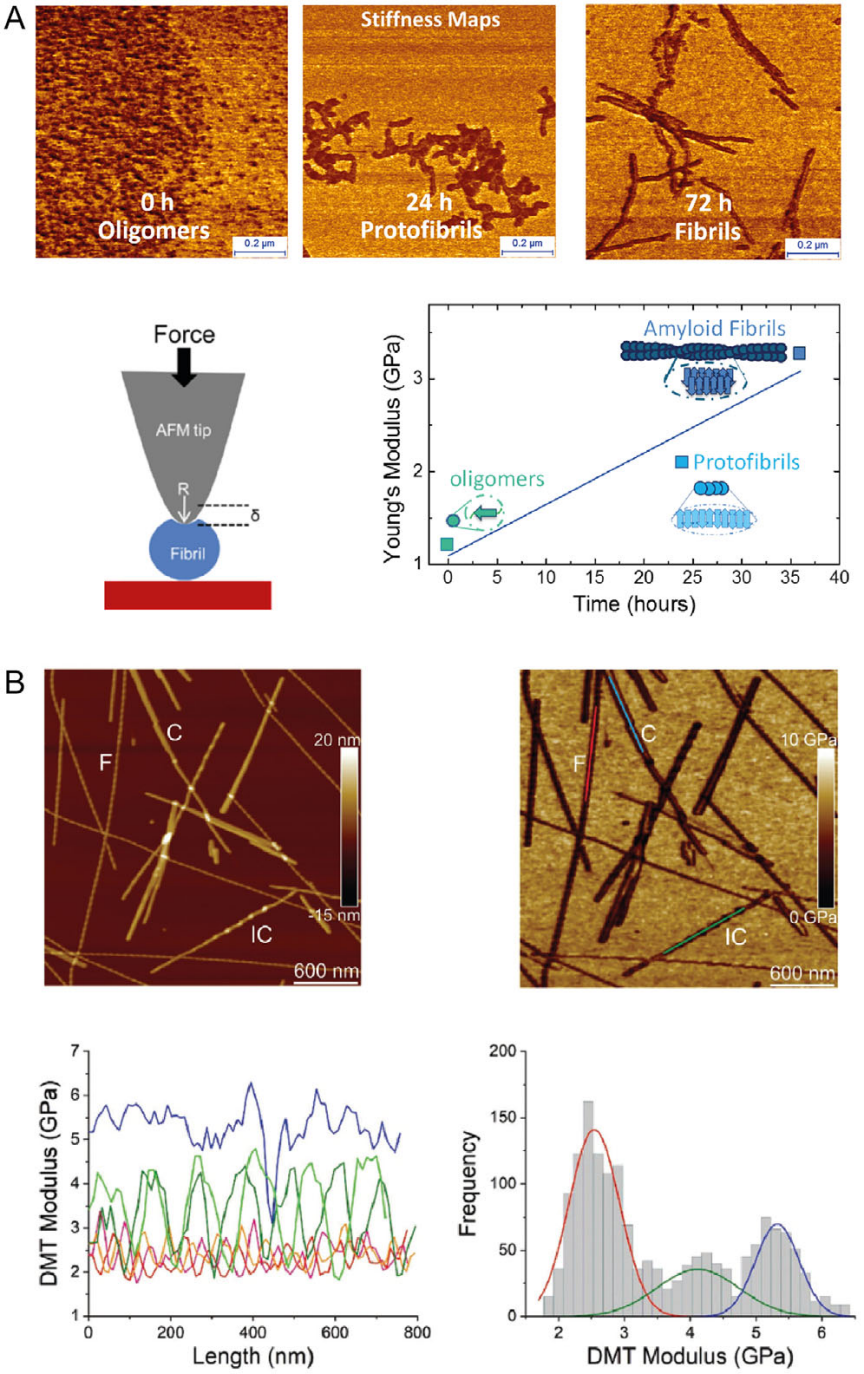
Determination of the heterogeneous mechanical properties of amyloid species. A) Young's modulus of Aβ_42_ protein self‐assemblies increases during the fibrillization process, from ≈1 GPa for oligomers, to ≈2 GPa for protofibrils and >3 GPa for mature amyloid fibrils, adapted and printed with permission from,^[^ ^69^ ^]^ Copyright 2015, Wiley. B) Model IFQINS peptides were used to investigate the heterogeneity of the nanomechanical properties of single amyloid periodic fibrils F, intermediate crystals IC, and crystals C. In the maps and cross‐sections, red color corresponds to the DMT moduli of fibrils, green shows intermediate crystals, and blue corresponds to the DMT moduli of crystals. Thus, demonstrating that different morphologies and crystal states of fibrils induce a significant variation in the mechanical properties; with the crystal state showing highest Young's modulus; printed with permission from,^[^ ^100^ ^]^ Copyright 2021, Wiley.

Nanoimaging is able to visualize the morphological changes protein undergo during their self‐assembly; however, it is not able to retrieve information on the structural changes and the mechanical properties of the formed species. Nanoindentation has thus been widely used to probe the heterogeneous mechanical properties of protein condensates and aggregates (adhesion, dissipation, elastic modulus) at the single‐molecule and condensate level, as well as within whole cells (**Figure** **10**). These studies highlighted the importance of nanomechanical imaging to determine the structural properties of heterogeneous protein self‐assemblies and cellular systems.

Early results, in the last decade, showed that peak‐force‐quantitative nanomechanics (PF‐QNM) allowed for the simultaneous collection of morphology maps and force‐distance curves with high spatial resolution.^[^ ^166^ ^]^ PF‐QNM was applied successfully to perform nanomechanical imaging and demonstrate that the intrinsic elastic moduli of different classes of mature amyloid fibrils, including α‐synuclein, Aβ_42_, and Tau proteins, lied in the GPa range.^[^ ^63^, ^165^ ^]^ This nanoindentation characterization also allowed the measurement of the intrinsic Young's modulus of protein aggregates independently of the polymorphic state and their cross‐sectional properties.^[^ ^166^ ^]^ In particular, it was able to capture the evolution of Young's modulus during fibrillation of α‐synuclein and Aβ_42_ (Figure 10A). The elastic modulus of the amyloidogenic species evolved monotonically during aggregation as a function of the fibrillation process. Oligomers, protofibrils, and mature fibrils showed an increasing value of intrinsic stiffness in the order of GPa.^[^ ^69^ ^]^ These results demonstrated that the amount of hydrogen bonding (H‐bonding) between β‐sheets in the assembled structures during oligomerization and fibrillization process is an important parameter affecting the mechanical properties of these structures. Other studies confirmed that the stiffness of amyloid fibrils increase because of H‐bonds formation. It is the case of peptides F8 and EF8E where E increases ≈0.07 GPa during self‐assembly upon addition of the first amino acid and ≈0.12 GPa each further added amino acid until 8 or 9 residues.^[^ ^291^ ^]^ No additional amino acids led to a change in Young's modulus *E*, indicating that no additional H‐bonds were formed. Similarly, it was confirmed that amyloid fibril maturity can affect mechanical properties. The high spatial resolution of nanomechanical imaging via AFM was further applied to study the heterogeneity of model amyloid fibrils formed by short peptides (Figure 10B),^[^ ^100^ ^]^ showing that amyloid fibrils exhibit sub‐molecular heterogeneity in mechanical properties; with periodic amyloid fibrils having the lowest Young's modulus in the order of ≈2.5 GPa, while a transition of the structure of these periodic fibrils to a crystalline rod‐like structure cause an increase of Young's modulus up to ≈5.5 GPa. The dependency of Young's modulus *E* on the structure and size of amyloid fibrils may be closely correlated with pathology molecular mechanisms since stiffer amyloid species might be more difficult to be handled by degradation and clearances processes.^[^ ^16^ ^]^

Finally, nanoindentation measurements were also used to prove how the presence of Aβ_1‐42_ peptide altered the mechanical properties of human neuroblastoma and rat hippocampal cells.^[^ ^315^, ^316^ ^]^ Theses result may suggest that Aβ_1‐42_ triggered microtubule disassembly and damage cellular neuronal membranes causing a softening effect related to the toxicity of oligomeric amyloids.

## 7 AFM‐IR: Nanochemical Imaging and Spectroscopy

We highlighted above AFM‐based methods providing unprecedented 3D information beyond imaging of morphology, largely by unraveling dynamics, molecular forces, and mechanical properties, for biomolecular processes in cancer and neurodegeneration. However, the chemical‐structural properties at the nanoscale of the biomolecules involved in these processes have profound impact on the onset and progress of these diseases.

Tip‐sample interaction forces are at the heart of an AFM detection system. These forces not only vary with tip‐sample distance, but also with the physical properties of the systems under investigation. This feature, together with the possibility to functionalize the AFM tip with arbitrary chemical groups, has further allowed discriminating different materials within the same sample on the basis of their thermal, electrical, and magnetic properties down to Ångström resolution.^[^ ^277^ ^]^ Yet, these AFM‐based physical methods do not allow to directly enquire with molecular recognition power the chemical properties of heterogeneous biological systems.

**Figure 11:**
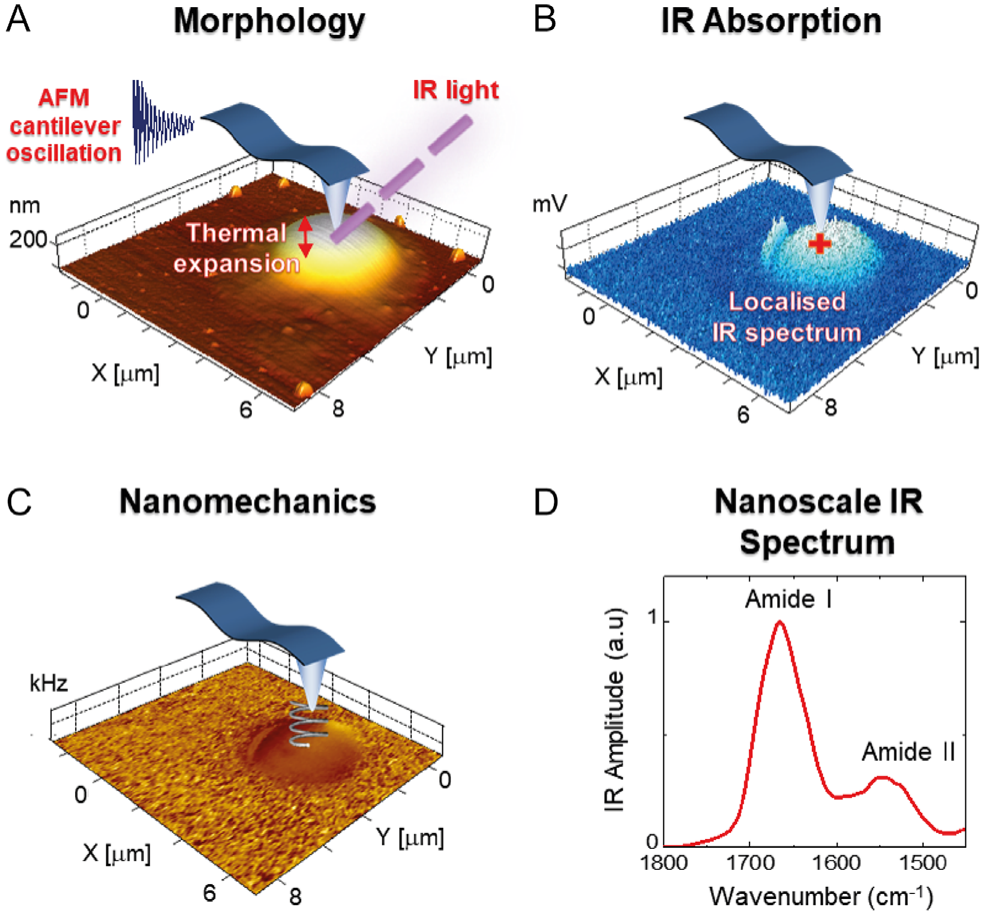
Principle of AFM‐IR function in protein condensates. A–C) Absorbed IR light causes thermal expansion of the sample exciting the AFM cantilever in contact with the sample, whose response is proportional to the A) absorbed light. Scanning the cantilever on the sample while fixing the laser wavelength enabled B) IR absorption and C) nanomechanical maps. D) Nanoscale localized IR spectra were obtained by sweeping the laser wavelength while fixing the position of the cantilever for a FUS protein condensate. Thus, AFM‐IR provides measurement of morphological, mechanical, and chemical properties. Figure adapted and printed with permission from,^[^ ^352^ ^]^ Copyright 2021, Bio‐Protocol.

A breakthrough has occurred in the last decade with the development and application of nanochemical analysis via AFM‐IR in biology. AFM‐IR combines the high spatial resolution of AFM (≈1 nm) with the chemical analysis power of IR spectroscopy, to allow multimodal imaging of morphological, mechanical, and chemical properties at the nanoscale (**Figure** **11**). The unique multimodal nano‐analytical capabilities of AFM‐IR have been leveraged to study heterogeneous biological systems over multiple physical scales, such as protein aggregates and condensates,^[^ ^35^, ^64^, ^79^, ^100^ ^]^ chromosomes,^[^ ^317^ ^]^ bacteria,^[^ ^318^ ^]^ viruses,^[^ ^319^ ^]^ vesicles,^[^ ^320^ ^]^ cells, and tissue,^[^ ^76^, ^318^, ^321^, ^322^ ^]^ among others. Because of its capabilities, the historical development of AFM‐IR has been actually driven by the need to unravel the heterogeneous properties of biomolecular and cellular processes with large focus on neurodegeneration and cancer research. Indeed, there is a compelling need to understand the chemical characteristics differentiating healthy from cancer cells and to correlate the chemical‐structural properties of protein condensates, assemblies, and amyloids to the origin of cytotoxicity in neurodegeneration.

### 7.1 Principles

To overcome the limitations of conventional nanoimaging methods, several AFM‐based nanospectroscopy techniques have been developed. These methods can be divided into: scattering methods, such as scanning near field optical microscopy (s‐SNOM)^[^ ^323^ ^]^ and tip‐enhanced Raman spectroscopy (TERS),^[^ ^65^ ^]^ and photothermal methods, such as AFM‐IR.^[^ ^65^, ^67^ ^]^ These approaches offer a trade‐off between high‐sensitivity and the ability to relate directly the acquired chemical information into quantitative chemical characterization. Scattering based methods, such as TERS and s‐SNOM empowered the capability to acquire chemical information at the nanoscale, even at the single‐molecule and single‐bond scale for TERS.^[^ ^324^ ^]^ However, the interpretation of such spectra in terms of direct structural information constraints the study of larger biomolecules that remains highly challenging. Indeed, scattering spectra are over‐rich in information and variability and subjected to geometric and plasmonic effects causing: suppression of bulk Raman bands in TERS, such as Amide I,^[^ ^325^ ^]^ and thickness dependency of chemical shifts in s‐SNOM.^[^ ^323^ ^]^ These factors render quantitative nanoscale structural analysis less direct than bulk methods, especially when studying heterogeneous biological systems. The introduction of AFM‐IR has allowed to overcome limitations of scattering methods in the quantitative analysis of biological systems. AFM‐IR achieve nanochemical analysis by leveraging the photothermal‐induced resonance effect (PTIR). The PTIR effect, in contrast to scattering methods, measures directly the IR absorption of a sample (Equation 21). Thus, the spectra are in full agreement with IR bulk approaches. The acquired chemical information is not affected by scattering and plasmonics effects, allowing reliable structural‐chemical analysis of biological samples from the micron to the single‐molecule scale.^[^ ^65^, ^66^ ^]^

Figure 11 illustrates the principle of function of AFM‐IR based on the PTIR effect, in the case of liquid–liquid phase separated protein condensates of FUS.^[^ ^35^ ^]^ A tunable IR laser is operated in the mid‐IR range of 3600–800 cm^−1^. The IR laser is focused on the AFM probe. If the wavenumber $\tilde{\nu }$ (cm^−1^) of the exciting laser pulse matches one of the molecular vibrational energy levels of the sample, the IR light is absorbed (Figure 11A). This absorption causes thermal heating and expansion of the sample, which is detected by the AFM tip in contact (CM) or tapping mode (TM).^[^ ^65^, ^67^ ^]^ The typical temperature increase induced by the IR laser is of only a few degrees Celsius, thus not inducing significant modifications even to soft biological material. The oscillation response *S*_*AFM‐IR*_ of the cantilever in contact with the sample is purely proportional to the IR absorbance $A(\tilde{\nu })$ at each wavenumber (Equation 22). In CM, the $A(\tilde{\nu })$ is proportional to the peak‐to‐peak amplitude of the raw deflection signal or the peak amplitude of the Fourier transform of the oscillation of the cantilever, termed the IR amplitude; while in TM, the $A(\tilde{\nu })$ is proportional to the cantilever response via a heterodyne detection, where one Eigenmode of cantilever oscillation is used to measure the topography of the sample and another Eigenmode is used to measure the thermal expansion.^[^ ^67^ ^]^ TM AFM‐IR reduces the tip‐sample interaction forces and allows less invasive measurement of soft materials, although the reduced time of interaction between the tip and the samples may cause a reduction of the IR signal. The IR laser source illuminates an area of ≈30 μm in diameter, but the spatial resolution advantage of AFM‐IR derives from the fact that the near‐field aperture of the IR detector is nominally the AFM tip diameter, which is as sharp as 1–10 nm. Scanning the probe over the sample at fixed IR laser wavelength enables the simultaneous acquisition of nanoscale maps of morphology (Figure 11A), IR absorption (Figure 11B), and mechanics (Figure 11C). The acquisition of mechanical information is achieved qualitatively by monitoring the cantilever resonant frequency, which varies monotonically as a function of the Young's modulus of the sample, or quantitatively via peak‐force based detection. Once the mapping is completed, nanolocalized spectra (Figure 11D) are obtained by sweeping the IR laser wavenumber while maintaining a fixed position of the AFM cantilever, with a lateral resolution down to ≈5 nm.^[^ ^326^ ^]^ AFM‐IR can also acquire 4D chemical hyperspectral maps, where the morphology is correlated to a chemical map where each individual pixel is a full IR spectrum. Thus, AFM‐IR acquires multimodal maps of 3D morphology, single wavenumber chemistry and stiffness, or 4D hyper‐spectral IR absorption maps.

We first provide a brief overview of the theoretical framework at the base of AFM‐IR (Figure 11A). A formal derivation of the AFM‐IR signal transduction *S*_AFM‐IR_ was first proposed in 2010^[^ ^327^ ^]^ and further rearranged^[^ ^328^ ^]^ and can be summarized in Equation (22).

(22) $$S_{\text{AFM}-\text{IR}}(\tilde{\nu })=H_{\text{AFM}}\cdot H_{\text{exp}}\cdot H_{\text{ther}}\cdot H_{\text{opt }}\cdot \tilde{\nu }\;\cdot \;k(\tilde{\nu })\;\propto \;A(\tilde{\nu })$$

where *H*_AFM_ is a constant depending on the cantilever's geometry, modal stiffness *k* (N m^−1^) and frequency of oscillation *ω* (s^−1^), and deflection sensitivity (nm V^−1^). *H*_exp_ is the mechanical contribution, which depends on the thermal expansion coefficient α_T_ (K^−1^) of the sample. *H*_ther_ is the thermal contribution depending on the dynamics of thermalization of the sample, as determined by its thermal properties and the choice of the IR laser pulse length *t*_p_ (ns). *H*_opt_ is the optical contribution, which in its simplest form is proportional to the intensity *I*_inc_($\tilde{\nu }$) of the incident IR light, which is known by acquiring a background spectrum of the IR laser profile. The term $\tilde{\nu }\cdot k(\tilde{\nu })$ is the product of the wavenumber $\tilde{\nu }=1/\lambda$ (cm^−1^) of the light and the imaginary part (extinction coefficient) of the complex refractive index $\tilde{n}=n(\tilde{\nu })+i\;k(\tilde{\nu })$ of the sample; thus, it is nothing else that the absorption coefficient of the sample $\alpha (\tilde{v})$ (cm^−1^). Since the terms *H*_AFM_, *H*_exp_, *H*_ther_, and *H*_opt_ only depend on constants and geometrical factors, they only scale the overall signal and do not affect relative peak intensities or shapes. Thus, *S*_AFM‐IR_ only varies as a function of the absorption coefficient $\alpha (\tilde{v})\propto \tilde{v}\;k(\tilde{v})$. Equation (22) thus demonstrated that the absorption‐induced thermomechanical expansion is directly proportional to the IR absorption $A(\tilde{\nu })$ of the sample.^[^ ^327^, ^329^ ^]^

Two AFM‐IR illumination geometries are available: bottom and top illumination. This illumination geometry fundamentally impacts the capabilities of the technique. The first and second generations of AFM‐IR relied on a bottom illumination scheme using transparent prism elements, and a standard AFM cantilever operating in contact mode as a detection system.^[^ ^329^, ^330^ ^]^ Pulsed IR excitations were initially provided by either a free electron laser with tunable wavelength or a carbon dioxide (CO_2_) laser operating at fixed wavelengths, with pulse widths *t*_p_ of 1 μs and 10 ns, respectively. The pulsed character of the excitation is required because the cantilever oscillation amplitude is sensitive to the derivative (speed) of the surface displacement rather than the displacement itself.^[^ ^327^, ^330^ ^]^ The limited performance of the first AFM‐IR experimental designs, owing to the large *t*_p_ of free‐electron lasers and the lack of wavenumber tunability of CO_2_ lasers, stimulated the development of novel solutions. Therefore, it was introduced a second‐generation AFM‐IR, leveraging optical parametric oscillator lasers (OPOs) with tunability between 4000 and 1000 cm^−1^ and *P*_w_ < 10 ns. The short pulse (10 ns) working regime, for *t*_p_ faster than the thermalization time of the sample, allowed that the spatial resolution to be mainly limited by the AFM tip size, while for pulse width longer than *t*_p_, the spatial resolution is only slightly affected by heat thermal diffusion.^[^ ^327^, ^330^ ^]^ However, OPOs performance was still limited by delivering a local IR spectrum in ≈5 min and a chemical map in ≈30 min, with 50–100 nm spatial resolution at 4–10 cm^−1^ spectral resolution. Furthermore, in the bottom illumination geometry, the pulsed IR beam undergoes attenuated total reflection (ATR) at the interface between the IR transparent prism and the sample surface; thus, the signal increase linearly with sample thickness only up to ≈1–2 μm.^[^ ^328^ ^]^ Another constraint is represented by the low IR absorption condition of the prism, which restricts the choice of substrate to transparent media, such as zinc sulfide (ZnS) and to zinc selenide (ZnSe).^[^ ^79^, ^331^ ^]^

To overcome limitations of OPO lasers and prisms elements, a third current generation of AFM‐IR devices was developed with top‐illumination of the laser.^[^ ^64^, ^65^, ^67^ ^]^ These devices leveraged as IR source quantum cascade lasers (QCL) lasers with: fast pulse (20–500 ns), large range repetition rate (*R*_r_) (1 kHz to 2–3 MHz), fast spectra acquisition (≈500 ms), and improved spectral resolution (≈0.5 cm^−1^). Since QCL single chips have limited tunability of ≈400 cm^−1^, multiple chips are however required for the mid‐IR range. The introduction of QCLs enabled the introduction of the resonance‐enhanced (RE) mode, where the laser *R*_r_ could be matched with one of the cantilever contact resonance eigenvalues.^[^ ^332^ ^]^ In RE mode, sample expansion cycles occurred at the same frequency as cantilever oscillations, which allowed for a sensitivity improvement of the order of the cantilever quality factor Q (up to 10^5^ in vacuum). Further improvements in sensitivity were achieved by adapting a top‐illumination scheme in which the IR field intensity was enhanced at the nanogap between a gold‐coated AFM tip and gold‐coated substrate.^[^ ^333^ ^]^ In the first application of this method, a molecular sensitivity of ≈30 molecules was estimated, along with a spatial resolution of 25 nm, which was smaller than the tip radius (≈30 nm).^[^ ^333^ ^]^

Latest developments in AFM‐IR technology have further open a new window of observation with nanochemical resolution on biomolecular processes at the single molecule and cell level, which were not possible and not even imagined more than a decade ago.

To allow nanochemical analysis of both bulk and surface properties, AFM‐IR surface sensitive mode (SSM) has been recently developed.^[^ ^67^ ^]^ In top illumination, AFM‐IR leverages the high penetration depth of IR light in the order of several micrometers: to measure the bulk properties of the samples beyond the limits of the ATR configuration (≈2 μm). Instead, scattering methods are mostly surface sensitive with depth resolution of ≈5–10 nm for TERS and 10–50 nm for s‐SNOM.^[^ ^65^ ^]^ SSM AFM‐IR is operating in contact mode uses heterodyne detection, to achieve the characterization of the most superficial layer of a material with depth sensitivity of ≈25 nm.^[^ ^67^ ^]^

Performing AFM‐IR in bottom illumination has been reconsidered in recent years to achieve measurements in physiological‐like environments in liquid.^[^ ^68^ ^]^ Indeed, its ATR configuration is preferable for liquid measurements since it reduces impact of water signal on AFM‐IR, as in bulk ATR‐FTIR.^[^ ^66^, ^331^ ^]^ Bottom illumination also has the advantage to not require metallic probes to reduce the IR absorption of the tip, but its sensitivity in liquid is yet limited to sample thicknesses >100 nm.^[^ ^68^, ^334^ ^]^

On the other hand, top illumination has proven to achieve the detection and chemical‐structural analysis of single molecules and their interaction, such as the secondary structure determination of protein and the chirality of single polymer chains.^[^ ^66^, ^70^, ^326^ ^]^ Top illumination allows employing the plasmonic enhancement at a metallic tip‐substrate nanogap and performing polarization‐dependent studies, to push AFM‐IR sensitivity to the single‐molecule level, which might be useful to improve sensitivity in liquid as well.^[^ ^66^ ^]^ Achieving single molecule sensitivity was possible thanks to the introduction of off resonance, low power, and short pulse (ORS)^[^ ^66^, ^70^ ^]^ and via the recent development of acoustically mechanically suppressed (AMS) AFM‐IR.^[^ ^326^ ^]^ When the sample size is smaller than the tip radius, a non‐negligible portion of the substrate is also excited and contributing to the signal, which is further frustrated by the external environmental noise. ORS and AMS AFM‐IR demonstrated that detuning off‐resonance the laser repetition *R*_*r*_ allows maximizing sample signal, reducing tip excitation from the substrate, and minimizing sample damage to study structure of biomolecules down to single amyloidogenic oligomers with a diameter of 3–4 nm, protein with ≈400 kDa,^[^ ^66^, ^70^ ^]^ and chiral chains of ≈200 kDa.^[^ ^326^ ^]^

### 7.2 Unravel Nano Chemical‐Structural Properties in Cancer Research

Cancer cells exhibit unique chemical and structural characteristics influencing their behavior and interactions with their environment.^[^ ^25^, ^27^, ^71^ ^]^ Unraveling the chemical–structural properties of cells and tissues is thus critical for understanding the complexities of tumor biology and developing effective treatments. AFM‐IR offers an invaluable tool to probe and differentiate the physical–chemical and structural properties of health,^[^ ^322^ ^]^ subjected to stress,^[^ ^321^ ^]^ and cancer cell at the nanoscale,^[^ ^76^ ^]^ as well as to study the properties of anticancer drug formulations^[^ ^335^ ^]^ and their effect and localization within the cellular and subcellular components.^[^ ^76^, ^317^ ^]^

Several studies have employed AFM‐IR to study the chemical properties of cancer cells. A common biomarker of breast cancer is represented by breast microcalcifications (BMCs), calcium‐rich deposits that can reach the millimeter size.^[^ ^336^ ^]^ While mammography X‐ray screening, accounts for presence, distribution, and shape of the BMCs, it does not provide any histological or chemical information. AFM‐IR has been used to overcome these limitations and perform a nanochemical analysis of BMCs from a carcinoma biopsy,^[^ ^337^ ^]^ showing that the physicochemical properties of BMCs correlated with the severity of the pathology, to help medical oncologists to design a tailored therapy. Similarly, AFM‐IR has been used to probe secondary structure changes within fibril‐rich and fibril‐poor areas of pleomorphic adenoma tissue sections.^[^ ^338^ ^]^ The importance of quantifying the biochemical heterogeneity of cancer cells has been further pointed out in a study aimed at characterizing morphological–chemical features of epithelial cancer cells in a spatial–temporal dependent manner.^[^ ^339^ ^]^ The nanochemical analysis of cancer cells via AFM‐IR has been further compared with bulk FTIR and Raman for the identification of cholesterol derivatives bands of PC‐3 prostate cancer.^[^ ^340^, ^341^ ^]^ AFM‐IR empower to detect chemical changes undetectable with FTIR and Raman. Moreover, polarization dependent studies allowed the enhanced study lipid bands in the cellular membrane,^[^ ^341^ ^]^ and null deflection mode has been proposed for the analysis of thin sections of model cell lines.^[^ ^322^ ^]^

**Figure 12:**
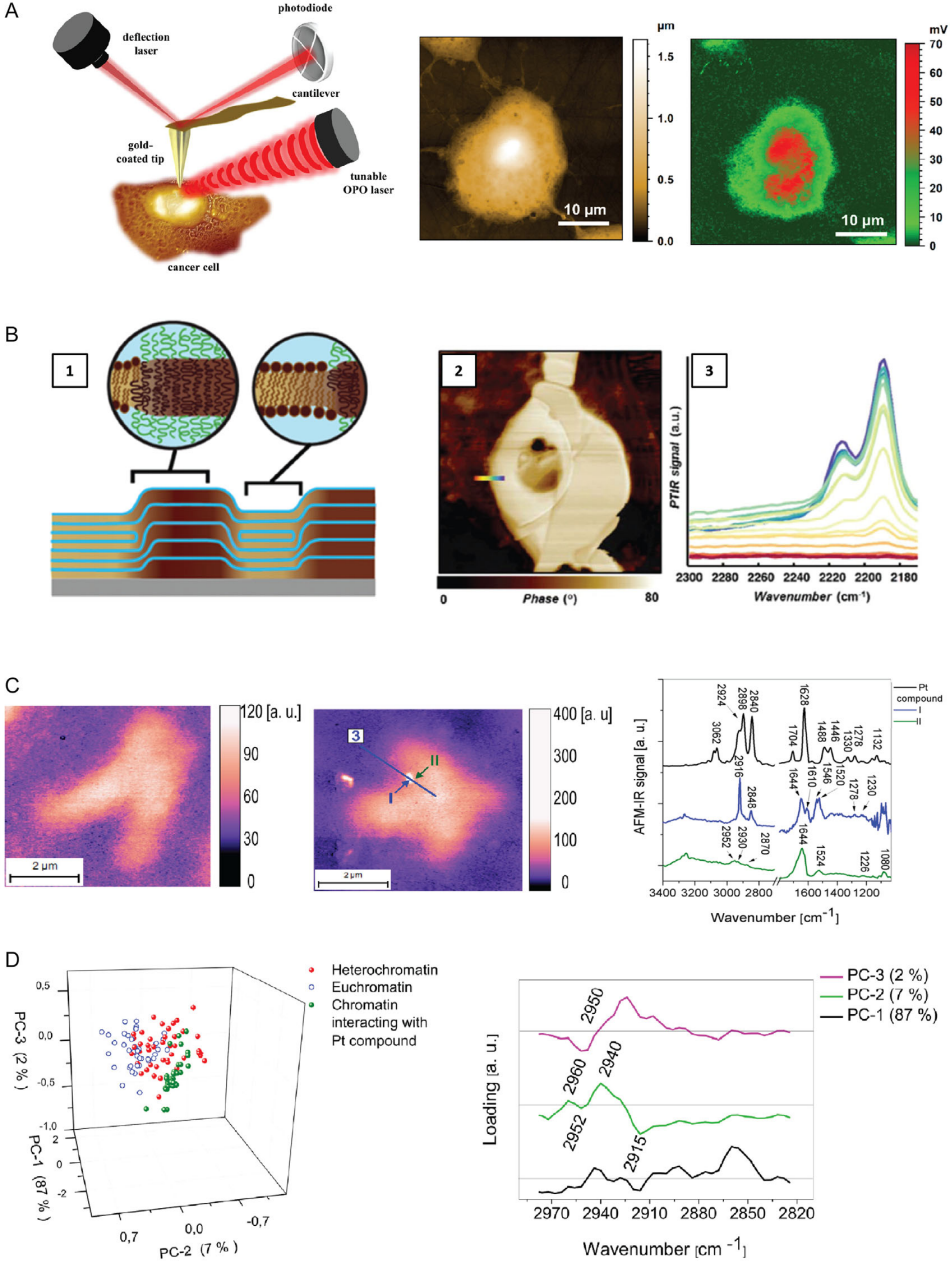
AFM‐IR applications in cancer research. A) AFM‐IR is used to localize a luminescence and IR active molecular probe within breast cancer cells, printed with permission from,^[^ ^76^ ^]^ Copyright 2023, Elsevier. The AFM‐IR chemical map has been collected at 1925 cm^−1^, where a strong absorption of the metal‐carbonyl occurs. B) Nanoscale spatially resolved IR spectra have been acquired at the interface of a hybrid lipid‐polymer membrane used to encapsulate the cancer drug paclitaxel (number 1 and 2), printed with permission from,^[^ ^346^ ^]^ Copyright 2018, The Royal Society of Chemistry. The decay of the intensity of lipid‐associated peaks (number 3) has been used to assess the size of the interface and to estimate the amount of lipid content within the polymer‐rich phase. AFM‐IR of chromosomes anti‐cancer drug interaction showing C) nanoscale IR maps (2916 cm^−1^) of metaphase chromosomes in presence and in absence of platinum‐based anticancer drugs. D) Chemometrics techniques have been used to discriminate heterochromatin and euchromatin regions based on the methylation content. Subsequently, it was possible to establish a preferential binding of the cancer pharmaceutical to heterochromatin. C–D) Adapted and printed with permission from,^[^ ^317^ ^]^ Copyright 2019, Oxford Academy.

The nanoscale resolution of AFM‐IR has further posed the way to study cancer at the sub‐cellular level. However, the overlapping of vibrational bands arising from the complex cellular environment can challenge the detection of cellular ultrastructure and molecular composition despite the advantage of label‐free detection. Thus, organometallic conjugate probes have been developed, injected, and localized in breast cancer cells to allow the detection of an exogenous agent inside cells, with spatial resolution <100 nm.^[^ ^342^ ^]^ The advantage of these molecular probes is that their vibrational peaks, related to metal‐carbonyl groups, appear in an IR transparent region for biological samples between 1900 and 2300 cm^−1^. In a following study, the IR properties of metal‐carbonyl units were combined with ligands known to exhibit luminescence, to allow correlative analysis.^[^ ^343^ ^]^ This multimodal probe was integrated in mestranol, a prodrug for ethynylestradiol and estrogen component of some oral contraceptives, to investigate distribution in breast cancer cells (**Figure** **12**A). The study showed the localization of the modified estrogen in the Golgi apparatus, showing how AFM‐IR could be successfully used in combination with luminescence techniques to characterize biological samples at the subcellular level.

Nanochemical imaging was further leveraged as a tool to assess physicochemical properties of anticancer drug formulations and improve their performances. Nanochemical analysis was used to study the partitioning of anti‐cancer drugs paclitaxel within hybrid lipid‐polymer membranes. These hybrid membranes, compared to single component formulations, showed enhanced releasing of the chemotherapeutic agent while simultaneously hindering its crystallization.^[^ ^344^, ^345^ ^]^ The study identified paclitaxel preferential localization, quantified the width of the phase boundaries of the two membrane components, and estimated the percentage of lipids in the polymer‐rich phase (Figure 12B).^[^ ^346^ ^]^ This information is promising to optimize the efficacy of hybrid membrane‐based delivery systems and produce anticancer drugs with improved performances. Another crucial problem in cancer therapies is represented by the cytotoxic activity exerted indistinctively on cancerous and healthy cells, which inhibits physiological functions leading to side effects. Noble metal‐based nanoparticles made of silver (AgNPs) and gold (AuNPs) have been proposed as potential candidates to selective release the drug load against cancer cells. Since the drug efficiency can be altered by its conjugation with the NPs, nanospectroscopy was used to perform a single‐particle chemical characterization of how the drugs interact with the NPs.^[^ ^335^, ^347^ ^]^ Similarly, AFM‐IR assessed the interaction of the anticarcinogenic drug erlotinib with Ag‐ and Au–NPs,^[^ ^348^ ^]^ showing that erlotinib interacted with the AgNP monolayer via its phenyl ring and methoxy moiety, while with the AuNP monolayer through its phenyl ring and the quinazoline moiety.

AFM‐IR has also proven able to assess the localization, distribution, and molecular interactions of cancer drugs at the sub‐cellular and organelle level. AFM‐IR demonstrated that a ferrocifen‐labeled chemotherapeutic agent localizes in the nucleus, providing direct answer to a question that was long debated.^[^ ^349^ ^]^ Similarly, the antitumoral properties of withaferin A^[^ ^350^ ^]^ were investigated for better understanding its mechanism of action on cervical cancer cells. AFM‐IR spatially resolved spectra, collected from cells exposed to increasing doses of drug showed an antagonistic correlation between the drug concentration and the intensity of DNA‐associated bands, the secondary structure of protein, and the apoptosis‐related vibrational peaks, thus allowing to propose the molecular mechanisms on the antitumoral activity of the drug.^[^ ^76^ ^]^ AFM‐IR was further employed for the label‐free detection for mapping the distribution of a platinum(Pt)‐based chemotherapeutics in metaphase chromosomes (Figure 12C).^[^ ^317^ ^]^ Heterochromatin and euchromatic regions were first distinguished through quantification of their methylation degree. Then, presence of the spectral markers of the Pt‐containing molecule were investigated and a preferential binding with heterochromatin was proven.

### 7.3 Nano Chemical–Structural Properties of Protein‐Self Assemblies in Neurodegeneration

Protein self‐assemblies with varying shape, post‐translational modifications, secondary structure, and content of cross‐β structure may be associated to different neurotoxic mechanisms. Substantial progress has been achieved by charactering the morphological and mechanical properties of protein condensates and self‐assembly at the single‐molecule level. Yet, the chemical nature of the self‐assemblies most prone to cytotoxicity, and the mechanisms by which they contribute to disease are unclear. Classical AFM‐based methods are in large part chemically blind and do not allow to unravel the chemical‐structural heterogeneity of pathological protein self‐assemblies, precluding to draw a correlation between their chemical properties in vitro*/*ex vivo and their molecular mechanism of toxicity in vivo.

To overcome this knowledge gap, the single‐molecule and multimodal capabilities of AFM‐IR have been leveraged to perform an accurate nanochemical characterization of the heterogeneity of the chemical‐structural diversity of the protein condensates and self‐assembled species formed in vitro and ex vivo, in relationship to their role in the onset of neurodegenerative disorders, such as AD, PD, HD, ataxia, and ALS.

**Figure 13:**
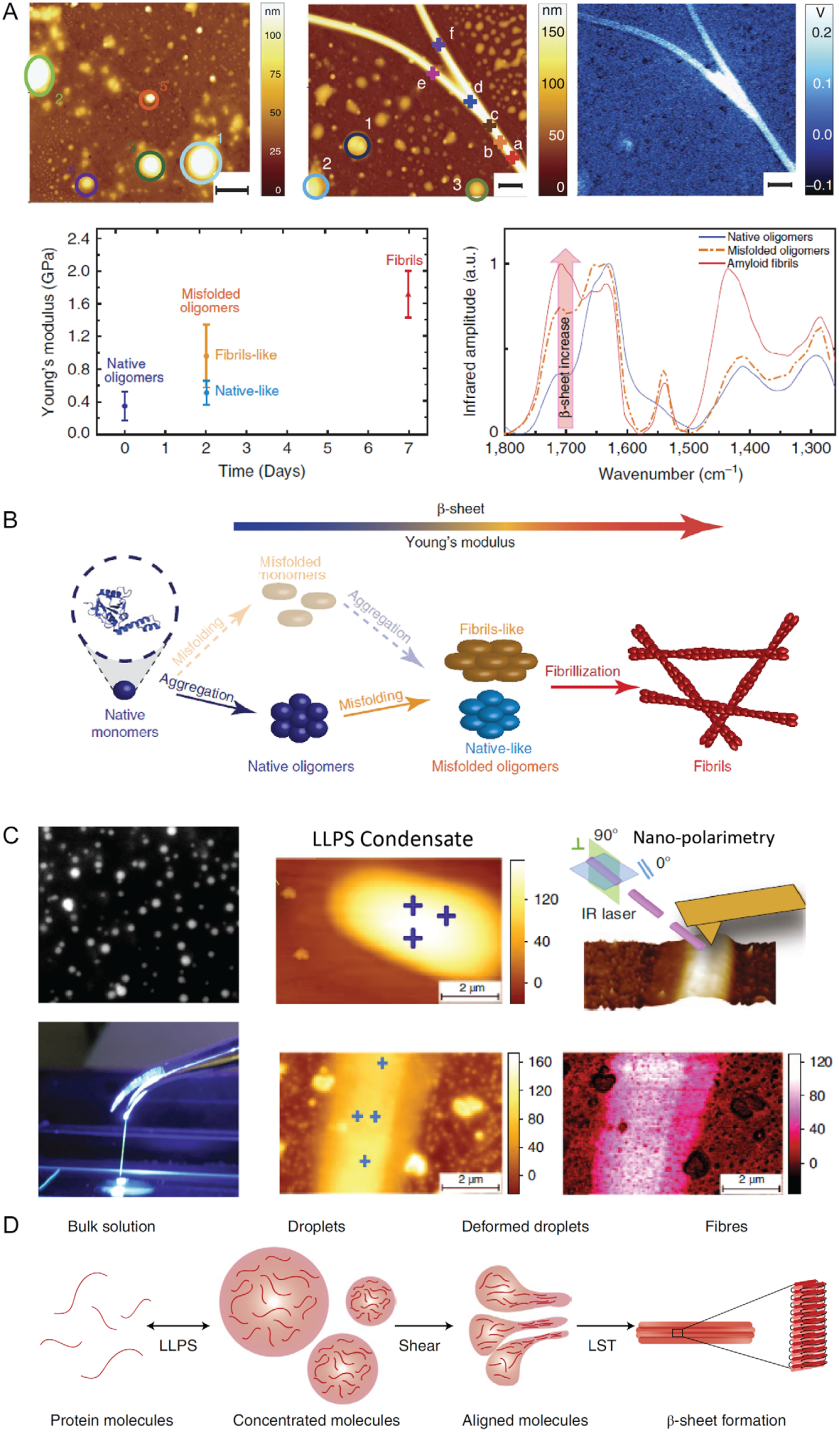
AFM‐IR application to study protein liquid‐liquid phase separation and aggregation. A) AFM‐IR analysis of the aggregation process of the Josephin domain of ataxin‐3 protein. Nanoscale resolved mechanical and chemical maps and spatially resolved IR spectra, discriminated secondary structure differences during early condensation and aggregation. B) Schematic representation of the hypothesized first “aggregation and then misfolding” mechanism for the Josephin protein. A–B) Adapted and printed with permission from,^[^ ^79^ ^]^ Copyright 2015, Springer Nature. C) Example of AFM‐IR application to the study of the liquid to solid transition of FUS protein. In this study, AFM‐IR in combination with optical microscopy and nano‐polarimetry demonstrated the shear‐induced ageing of the FUS protein. D) The solid and ordered fibrillar state was driven by the droplet deformation induced by shear forces. C–D) Adapted and printed with permission from,^[^ ^353^ ^]^ Copyright 2020, Springer Nature.

In the very first study of this kind, nanochemical imaging and spectroscopy was applied to study the link between protein self‐assembly of the Josephin domain of the ataxin‐3 protein and the neurodegenerative disorder of Ataxia‐3 (**Figure** **13**).^[^ ^79^ ^]^ Yet with limited sensitivity to samples with ≈50 nm thickness, AFM‐IR was successfully applied to characterize oligomeric and fibrillar species of the Josephin domain (Figure 13A), correlating the intrinsic stiffness of amyloids with their secondary structure content at the nanoscale (Figure 13A).^[^ ^79^ ^]^ The study suggested that the aggregation of Josephin proceeds from the monomer state through the formation of spheroidal intermediates with a native structure, which may later be identified as condensates (Figure 13B). These intermediates evolved into misfolded aggregated condensates, leading to amyloid fibrils formation. This study demonstrated that proteins were still in their native conformation at the earliest stage of aggregation, providing the first direct evidence of the “first‐aggregation‐and‐then‐misfolding” pathway of amyloid formation. Similarly, this approach was leveraged to study how the heterogeneity of amyloid species Aβ_42_ had increased cytotoxicity when subjected to pyroglutamylation. The study found that a 5% content of pyroglutamylation induced the formation of oligomeric species and with increased disruption of intracellular calcium homeostasis and the highest neuronal toxicity.^[^ ^351^ ^]^

AFM‐IR was further leveraged to understand protein behavior in the emergent field of biomolecular condensation and its link to the onset of neurodegenerative diseases, such as ALS (Figure 13C,D). Infrared nanospectroscopy was employed to first prove a phase transition from a liquid‐like to a disease‐associated solid‐like state.^[^ ^139^ ^]^ The technique first proved to be a powerful tool to locally probe the composition and assess the presence of intermolecular hydrogen bonding within single condensates (Figure 13C).^[^ ^352^ ^]^ Then, it was used to assess the effect of post‐translational modifications on the physiological state of the FUS protein condensates.^[^ ^35^ ^]^ The study proved that cooperative cation‐π interactions drive FUS condensation and that the ALS‐associated arginine hypomethylation strongly destabilize the physiological state of FUS condensates leading to intermolecular β‐sheet‐rich hydrogels disrupting ribonucleoprotein granule function and impairing new protein synthesis in neuron terminals. To further understand how physiological state of FUS could be altered, a subsequent study proved that shear could favor the liquid‐to‐solid transition of FUS into macroscopic fibrillar aggregates, similar to classical amyloids with high content of intermolecular hydrogen bonding (Figure 13C).^[^ ^353^ ^]^ AFM‐IR nanopolarimetry unraveled the structural order of single fibrils and reconstructed the mechanisms of FUS fibrillization induced by low mechanical shear favoring generic backbone–backbone hydrogen bonding and intermolecular β‐sheet formation (Figure 13D).

While instrumental, the initial sensitivity of AFM‐IR prevented to characterize single oligomeric and fibrillar species of smaller protein and peptides. A pivotal study leveraged the third generation of AFM‐IR systems, combined with the resonance‐enhanced mode and the rod‐like antenna effect, to achieve nanochemical analysis of single amyloid species.^[^ ^64^ ^]^ This study provided novel information on the role of polyQ expansion in altering the structure of amyloids in association to the genetic onset of HD. AFM‐IR demonstrated that polyQ content determines a the ordering of the cross‐β sheet structure of huntingtin exon 1 aggregates.^[^ ^64^ ^]^ Oligomeric species only presented antiparallel cross‐β sheet structure, while amyloid fibrils had an increasing content of parallel cross‐β sheet structure as a function of increasing polyQ expansion. Overall, the correlation of AFM‐IR results with morphological and nanomechanical analysis suggested that crossing the polyQ pathogenic threshold causes a chemical structural reorganization of amyloid species, which could be potentially linked with their toxicity mechanism in HD.

**Figure 14:**
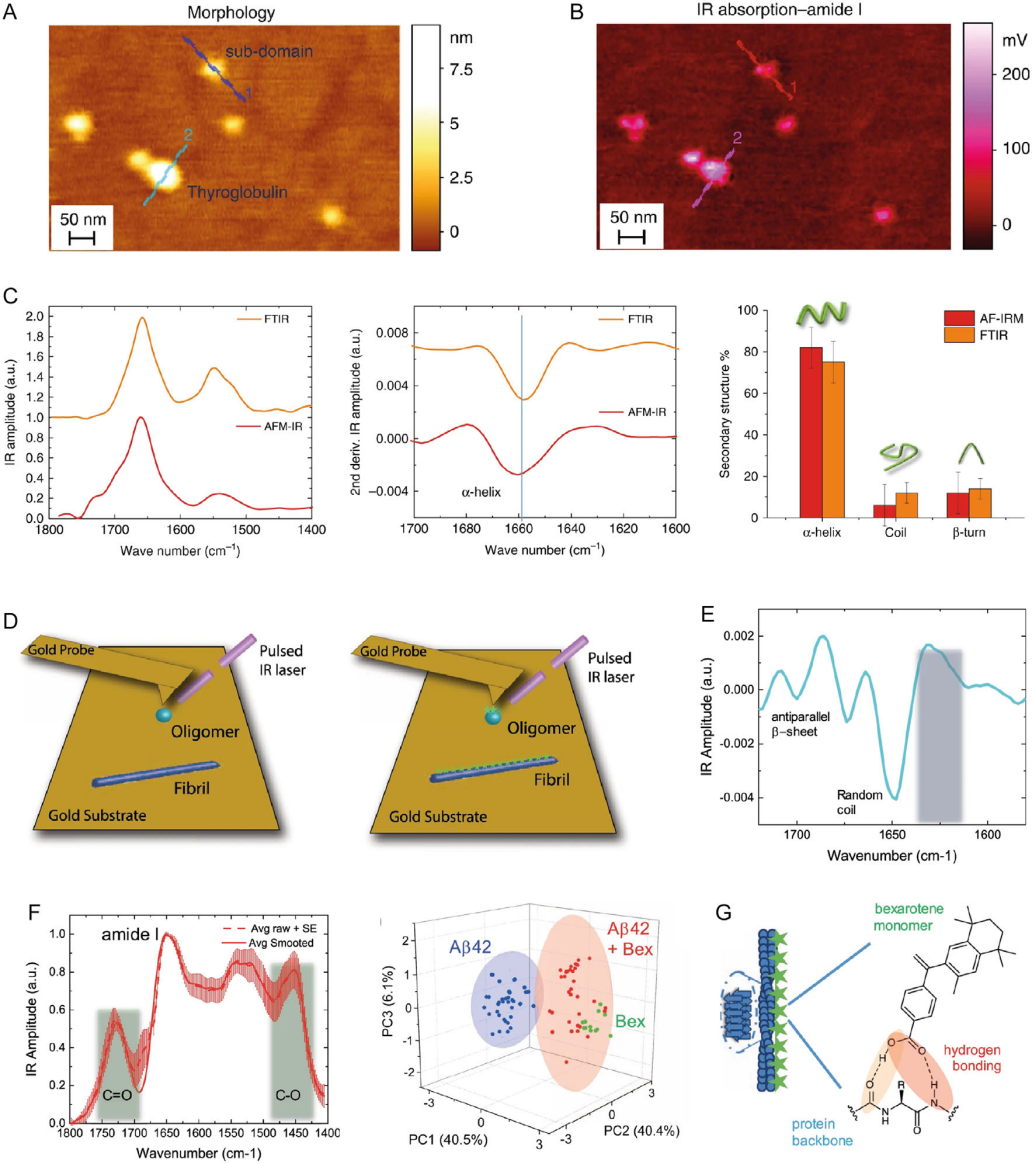
Single‐molecule AFM‐IR studies of single protein and their assemblies. A–C) Adapted and printed with permission from^[^ ^66^ ^]^ (Copyright 2020, Springer Nature), single‐molecule AFM‐IR to study single thyroglobulin protein A) 3D morphological map and B) AFM‐IR chemical map collected at 1655 cm^−1^ (amide I). C) Single molecule AFM‐IR spectra acquired versus conventional bulk IR spectra, with second derivative analysis, demonstrated that AFM‐IR can determine the secondary structure of single‐protein with similar accuracy as FTIR. D) Schematic of the AFM‐IR experimental approach to study the interaction of Aβ_42_ oligomers (light blue sphere) and fibrils (dark blue rod) with the anti‐aggregation drug bexarotene (green star). To reach high sensitivity ORS nanoIR at the nanogap between a gold‐coated probe and a gold substrate was used. E) Second derivative of the spectrum of a single‐aggregate in absence of the bexarotene drug to determine its secondary structure. F) Single‐aggregate IR spectrum collected in presence of the bexarotene molecule, with PCA analysis showing the possibility to discriminate spectral differences between Aβ_42_ in absence and in presence of the drug molecule. G) Schematic representation highlighting hydrogen bonding mediated interaction between bexarotene and Aβ_42_. D–G) Adapted and printed with permission from,^[^ ^70^ ^]^ Copyright 2021, Springer Nature.

Leveraging latest AFM‐IR capabilities and taking inspiration from the abovementioned studies, nowadays, extensive studies on protein condensation and aggregation have been carried out at the single condensate, oligomeric, and fibrillar levels. The recent development of ORS‐nanoIR has allowed to study protein structure with high‐signal‐to‐noise ratio down to a molecular weight of ≈400 kDa with ≈10–15 nm spatial resolution (**Figure** **14**A,B).^[^ ^66^, ^70^ ^]^ This development allowed in turn to determine the secondary structure of protein, with similar accuracy as conventional FTIR (Figure 14C). The ≈400 kDa limit well correspond to the typical size of the earliest forms of oligomeric and fibrillar species of amyloids, thus opening the routine analysis of their single‐molecule chemical–structural properties.^[^ ^66^, ^354^ ^]^

Within the many diseases‐related protein and peptides, significant efforts have been devoted to the study of α‐synuclein and Aβ amyloid species (Figure 14D), involved in the onset of PD and AD.^[^ ^66^, ^70^, ^351^, ^355^, ^356^, ^357^, ^358^, ^359^, ^360^ ^]^ These studies demonstrated at the single molecule level that oligomers of α‐synuclein and Aβ_42_ were characterized by antiparallel β‐sheet secondary structure signature, suggesting a potential correlation between this structure and toxicity (Figure 14E).^[^ ^361^, ^362^ ^]^ The structure of individual α‐synuclein and Aβ_42_ oligomers was further investigated in the presence of external factors.^[^ ^70^, ^357^ ^]^ In the case of α‐synuclein, the properties of the formed amyloid species were studied in presence of chemically different phospholipid vesicles and cholesterol,^[^ ^355^, ^356^ ^]^ which is relevant for the onset of PD since α‐synuclein function is related to synaptic vesicles function. These studies revealed structural differences between amyloid oligomers and fibrils grown in the presence and absence of phospholipids and the different incorporation of lipids and secondary structure of amyloids as a function of the lipid/protein ratio.^[^ ^359^ ^]^ The effects of the same phospholipid vesicles on the time‐dependent evolution of oligomer secondary structures were further investigated to prove an increase in α‐helix and unordered protein secondary structures at the expense of the parallel β‐sheet content.^[^ ^358^ ^]^ These studies showed that AFM‐IR paved the way to study protein‐lipid interactions with unprecedented detail. In the case of oligomeric and fibrillar species of Aβ_42_, AFM‐IR was combined with chemometrics to prove the molecular interaction of oligomers and fibrils with an aggregation inhibitor drug, bexarotene, which is able to prevent Aβ_42_ aggregation in vitro and reverse its neurotoxicity in cell and animal models of AD. This approach demonstrated that bexarotene interacts with Aβ_42_ oligomers via single hydrogen bond, proving to unravel protein‐drug interactions at the single molecules level (Figure 14G).

These results thus paved the way to establish AFM‐IR as a new single‐molecule tool to unravel the chemical–structural origin of amyloid species potential toxicity, as well as in structure‐based drug discovery programs against protein misfolding neurodegenerative diseases.

## 8 Conclusions and Future Perspectives

All the advances reported in this review demonstrate the continuous necessity to develop nanobiomedicine platforms that take advantage of information at the nanoscale beyond imaging. This review has discussed in detail how AFM‐based methods are a powerful tool to unravel in a multiscale and multimodal manner, the nanoscale properties of the common molecular processes that drive cancer and neurodegenerative disorders. The valuable information provided by AFM‐based methods can help to better understand the molecular origins underlying the onset and progression of these human pathologies. Furthermore, the identification of these common molecular mechanisms could be used to define biomarkers of disease, which in turn could be leveraged as diagnostic tool to test therapeutic strategies. To undertake this societal challenge, we have discussed the huge progresses achieved in the last two decades, with the development of sensitive AFM single‐molecule and video‐rate imaging, molecular recognition and mechanical investigations, as well as the latest developments in nanochemical analysis.

Despite the milestone advances of AFM‐based methods in bioscience, there is the compelling need to overcome several hurdles to further converting these significant gains into therapeutic applications in cancer and neurodegeneration. These challenges include: standardizing samples preparations and procedures of measurement; advancing imaging under physiological conditions; integration of AFM with correlative imaging methods; and improving raw data acquisition and processing, in combination with chemometrics and artificial intelligence (AI).

Sample preparation via deposition on a surface is still a critical step for the AFM‐based analysis of single molecules, cells, and tissues for their analysis in air and liquid environment. Despite the major advances in the development of new AFM modes and their applications, the science of sample preparation for AFM measurements is significantly based on the manual skills of the operator, and limitations remain in the accuracy and reproducibility of this key step in the analysis. Especially when measuring in air, the deposition of the sample on a surface by unexperienced users and newcomers can induce alteration of its properties compared with its natural state, which in turn can lead to misinterpretation of results and wrong conclusions. To deal with this limitation, in the case of biomolecules and small cells, automated and reproducible microfluidics spray deposition has shown preserving the molecular conformation and heterogeneity of samples prior to nano‐analysis via AFM‐based methods.^[^ ^152^, ^153^ ^]^

Cells and tissue samples must often be frozen, fixed, or immobilized for AFM analysis, which can interfere with studying dynamic cellular processes in a physiologically relevant state and may alter cell morphology and behavior. Thus, advancing the capability to analyze sample chemical and structural properties in a liquid environment, which resembles as much as possible the physiological conditions in human body, will be fundamental to advance our knowledge on neurodegeneration and cancer. Live‐cell AFM allows maintaining a fluid environment that mimics in vivo conditions, for the study of live, dynamic cellular behaviors while reducing the need for immobilization. This approach is particularly useful for cancer cells, which often exhibit distinct behaviors in different conditions.^[^ ^363^ ^]^ AFM‐based nanospectroscopy methods have shown proof‐of‐concept of operation in liquid,^[^ ^68^ ^]^ but they still have limited sensitivity and lack the possibility to control the liquid environment conditions. Further development to acquire nanochemical information at the single‐molecule level, or in biofluids and tissue, in liquid will be key for the routine operation of these methods in interdisciplinary laboratories aiming to tackle fundamental questions in neurodegeneration and cancer research. To partially overcome this limitation, the microfluidics spray deposition described above allow studying biomolecular processes in air, while preserving in a similar state as in liquid the molecular conformation and heterogeneity of biomolecules.^[^ ^152^, ^153^ ^]^ Yet, we believe that the reliable extension of AFM‐IR application in liquid will be an exciting challenge in the next decade. Indeed, if AFM‐IR operation in liquid proves reliable, it would allow in principle to perform nanospectroscopy in a dynamic physiological environment, paving the way to monitor molecular processes and interactions in real time at the nanoscale.

To monitor events in real‐time, the development of high‐speed AFM has allowed for faster data acquisition and enabling real‐time imaging of biomolecular and cellular processes, with time scales closer to other techniques such as fluorescence or electron microscopy. Yet, HS‐AFM needs to overcome key technical limitations. Innovations in AFM probe materials, such as carbon nanotube tips or diamond‐coated probes, could improve durability and ability to maintain high resolution. Anti‐adhesive coatings on AFM tips could also minimize contamination and improve measurement accuracy. Incorporating these techniques into bioscience research could provide more dynamic data without compromising sample integrity. Utilizing lower force set‐points or force‐modulation techniques could minimize the damage of fragile biomolecules and cells during imaging. Thus, advances in noncontact and tapping modes are required to reduce the force applied to biomolecules, cells, and tissue, to allow preserving the integrity of the sample while improving image quality. Furthermore, biomolecular interactions, such as those between oncoproteins and their target genes during cancer development or the oligomerization of protein leading to insoluble fibrils in neurodegenerative diseases can occur in an extremely short time scales. To capture transient intermediates at much higher temporal resolutions, HS‐AFM scanning speed must be increased. Towards achieving these goals, the speed of HS‐AFM was recently doubled using an ultrafast piezoelectric Z‐scanner.^[^ ^364^ ^]^ Furthermore, HS‐AFM has been recently modified to achieve much faster scanning speed and gentler tapping force, to capture the morphology of amyloid fibrils in a less invasive manner.^[^ ^58^, ^364^ ^]^

AFM has been further been combined with other techniques to improve its capabilities and make it a more versatile technique. Integration of AFM with advanced optical systems leveraging microlenses has enabled to bridge the resolution gap between traditional optical imaging and AFM, allowing to improve cross‐scale rapid imaging with micrometer to nanometer resolution, with higher throughput by a factor of ≈8.^[^ ^365^ ^]^ Live cell topography imaging data can be also achieved combining holotomography microscopy with AFM,^[^ ^231^ ^]^ which allows to gather the morphology of cellular and organoid samples as conventional AFM measurements, but also the mapping of key factors involved in disease as the mass density or the protein content due to the detection of small shifts in their refractive index.^[^ ^366^ ^]^

AFM also offers *trans*‐compatibility with electron microscopy (EM).^[^ ^367^ ^]^ The combination with scanning electron microscopy (SEM) was exploited to characterize tumor‐derived extracellular vesicles and visualize the location of their position markers,^[^ ^368^ ^]^ which are significant in the design of smart customized targeted therapies. Alternative approaches have been optimized to decipher the mechanisms underpinning the onset of cancer and amyloidogenic proteins. Cryo‐EM is one of the most promising techniques for the structural preservation of biological samples by vitrification based on ultrafast water freezing.^[^ ^209^ ^]^ Proper sample vitrification sustains its native hydrated environment beyond atomic resolution. Thus, cryo‐EM has revealed the folding mechanisms of macromolecular assemblies, such as Aβ_42_.^[^ ^369^ ^]^ Nevertheless, cryo‐EM suffers from some drawbacks, including: the necessity of image restoration by motion corrections made with dynamic interactions^[^ ^370^ ^]^ that requires class‐averaging and does not work purely at the single‐molecule level; it has difficulties in vitrifying large volume bodies, such as organelles, cells, organoids, multicellular organisms, or tissues, which could enormously limit its use in cancer research. To overcome some of these limitations, a focused ion beam (FIB) was used to slice sample sections and produced lamellae of nearly 100‐200 μm thickness for further cryo‐EM analysis;^[^ ^371^ ^]^ however, it may cause compression effects in the cutting direction leading to artifacts. Therefore, cryo‐EM can complement AFM measurements but does not work as a substitutive technique.

Correlative AFM imaging with fluorescence is further able to selectively identify biomolecules and cell types in vitro and within a solid biopsy,^[^ ^372^ ^]^ which is not possible with conventional AFM‐alone measurements. Correlative AFM‐based techniques and fluorescence are able to unequivocally distinguish between several cellular populations and could open the gate to assess the mechanical and chemical phenotyping of individual cells in heterogeneous samples, which is of particular interest in cancer.^[^ ^373^ ^]^ For example, a fluorescent probe called Flipper‐TR has been shown able to monitor membrane tension changes by inserting into lipid membranes.^[^ ^374^, ^375^ ^]^ This fluorescent probe combined with AFM nanomechanical studies could be also applicable to study the mechanical changes that the cellular membrane undergoes in cancerous cells. In an analogous manner, correlative imaging using amyloid sensitive dyes (thioflavin, fluorescent antibodies) could be successfully used to identify protein condensates and self‐assemblies in vitro and within more complex human biofluids and biopsies. Moreover, conventional AFM imaging is a surface‐sensitive technique, which restricts its analysis to the surfaces of samples. By contrast, AFM‐IR allows to study the chemical properties of biomolecules, tissues, and cells thanks to the high penetration depth, in the order of micrometers, of infrared light. Thus, the combination of AFM‐IR with fluorescence poses the basis to assess the inner properties of large biomolecular complexes, and of subcellular structures and molecules, narrowing the gap between surface imaging and intracellular information.

AFM‐based methods further offer the possibility to feed the morphological, mechanical and chemical information provided to machine learning and AI methods for the unbiased discrimination of healthy versus disease states at the nanoscale. The integration with automation and AI will help in analyzing large datasets, identifying patterns, and improving experimental reproducibility. Machine learning could enhance processing of AFM morphology maps, distinguishing artifacts from true molecular and cellular features, while automation reduces user‐related inconsistencies in measurements, which could be particularly valuable in biosciences research, where sample sizes and data complexity are high. AFM high‐speed and high‐resolution imaging could be further complemented with other tools in structural biology, such as AlphaFold.^[^ ^376^ ^]^ AFM‐IR combined with unsupervised clustering and chemometrics offers the opportunity for developing robust methods allowing the identification of nanochemical biomarkers, which could be used to monitor disease as a function of potential therapeutic strategies.^[^ ^373^ ^]^ Moreover, the creation of open‐access dataset libraries with AFM experimental data and settings will benefit the reproducibility and training of AI models.

For all the aforementioned reasons, the future prospects of AFM are excellent, not only to answer questions about key factors involved in cancer and neurodegenerative diseases, but also since AFM has the potential to interrogate other human diseases by ameliorating the current nanobiomedicine approaches. Indeed, it is important to note that single‐molecule studies using AFM could be successfully further devoted not only to cancer cells or amyloidogenic proteins, but also to all protein machinery assemblies and drug carrier agents involved in human pathologies.

## Supporting Information

Supporting Information is available from the Wiley Online Library or from the author.

## Conflict of Interest

J.C. is a co‐founder and shareholder of TargTex S.A ‐Targeted Therapeutics for Glioblastoma Multi‐forme. J.C. is a member of the Global Burden Disease (GBD) consortium of the Institute for Health Metrics and Evaluation (IHME), University of Washington (US), and the Scientific Advisory Board of Vector Bioscience, Cambridge. The other authors declare no conflicts of interest.

## Author Contributions

**Carlos Marcuello**: conceptualization (lead); visualization (equal); writing—original draft (equal); and writing—review and editing (equal). **KeeSiang Lim**: visualization (equal); writing—original draft (equal); and writing—review and editing (equal). **Giacomo Nisini**: visualization (equal); writing—original draft (equal); and writing—review and editing (equal). **Vadim S. Pokrovsky**: visualization (equal); writing—original draft (equal); and writing—review and editing (equal). **João Conde**: visualization (equal); writing—original draft (equal); and writing—review and editing (equal). **Francesco Simone Ruggeri**: conceptualization (lead); supervision (lead); visualization (lead); writing—original draft (lead); and writing—review and editing (lead).

## Supporting information

Supplementary Material

## References

[1] C. Global Burden of Disease Cancer , J. M. Kocarnik , K. Compton , F. E. Dean , W. Fu , B. L. Gaw , J. D. Harvey , H. J. Henrikson , D. Lu , A. Pennini , R. Xu , E. Ababneh , M. Abbasi-Kangevari , H. Abbastabar , S. M. Abd-Elsalam , A. Abdoli , A. Abedi , H. Abidi , H. Abolhassani , I. A. Adedeji , Q. E. S. Adnani , S. M. Advani , M. S. Afzal , M. Aghaali , B. O. Ahinkorah , S. Ahmad , T. Ahmad , A. Ahmadi , S. Ahmadi , et al., JAMA Oncol. 2022, 8, 420. 34967848 10.1001/jamaoncol.2021.6987 PMC8719276

[2] G. B. D. N. S. D. Collaborators , Lancet Neurol. 2024, 23, 344.38493795

[3] H. M. Bizuayehu , K. Y. Ahmed , G. D. Kibret , A. F. Dadi , S. A. Belachew , T. Bagade , T. K. Tegegne , R. L. Venchiarutti , K. T. Kibret , A. H. Hailegebireal , Y. Assefa , M. N. Khan , A. Abajobir , K. A. Alene , Z. Mengesha , D. Erku , D. A. Enquobahrie , T. Z. Minas , E. Misgan , A. G. Ross , JAMA Network Open 2024, 7, e2443198. 39499513 10.1001/jamanetworkopen.2024.43198 PMC11539015

[4] M. T. Manzari , Y. Shamay , H. Kiguchi , N. Rosen , M. Scaltriti , D. A. Heller , Nat. Rev. Mater. 2021, 6, 351. 34950512 10.1038/s41578-020-00269-6 PMC8691416

[5] O. Hassin , M. Oren , Nat. Rev. Drug Discov. 2023, 22, 127. 36216888 10.1038/s41573-022-00571-8 PMC9549847

[6] K. Kulenkampff , A.‐M. Wolf Perez , P. Sormanni , J. Habchi , M. Vendruscolo , Nat. Rev. Chem. 2021, 5, 277. 37117282 10.1038/s41570-021-00254-9

[7] L. Farhoudi , S.-F. Fobian , A. L. Oei , M. Amin , M. R. Jaafari , T. L. M. ten Hagen , Nano Today 2023, 53, 102032.

[8] M. J. Hajipour , H. Mohammad-Beigi , I. Nabipour , N. Mahmoudi , M. Azhdarzadeh , H. Derakhshankhah , D. E. Dawud , R. Mohammadinejad , D. E. Otzen , Nano Today 2020, 35, 100983.

[9] S. Chen , Z. Cao , K. Prettner , M. Kuhn , J. Yang , L. Jiao , Z. Wang , W. Li , P. Geldsetzer , T. Bärnighausen , D. E. Bloom , C. Wang , JAMA Oncol. 2023, 9, 465. 36821107 10.1001/jamaoncol.2022.7826 PMC9951101

[10] A. Nandi , N. Counts , S. Chen , B. Seligman , D. Tortorice , D. Vigo , D. E. Bloom , EClinicalMedicine 2022, 51, 101580. 35898316 10.1016/j.eclinm.2022.101580 PMC9310134

[11] S. J. Virolainen , A. VonHandorf , K. C. M. F. Viel , M. T. Weirauch , L. C. Kottyan , Genes Immun. 2023, 24, 1. 36585519 10.1038/s41435-022-00192-6 PMC9801363

[12] S. Wu , W. Zhu , P. Thompson , Y. A. Hannun , Nat. Commun. 2018, 9, 3490. 30154431 10.1038/s41467-018-05467-z PMC6113228

[13] F. Cavaliere , S. Gülöksüz , npj Mental Health Res. 2022, 1, 20. 10.1038/s44184-022-00018-3 PMC10956007 38609523

[14] M. Kampmann , Nat. Rev. Neurosci. 2024, 25, 351. 38575768 10.1038/s41583-024-00806-0

[15] S. V. Liu , M. Nagasaka , J. Atz , F. Solca , L. Müllauer , Signal Transduction Targeted Ther. 2025, 10, 111. 10.1038/s41392-025-02161-7 PMC11994825 40223139

[16] F. Chiti , C. M. Dobson , Annu. Rev. Biochem. 2017, 86, 27. 28498720 10.1146/annurev-biochem-061516-045115

[17] T. P. J. Knowles , M. Vendruscolo , C. M. Dobson , Nat. Rev. Mol. Cell Biol. 2014, 15, 384. 24854788 10.1038/nrm3810

[18] P. G. Needham , C. J. Guerriero , J. L. Brodsky , Cold Spring Harb Perspect. Biol. 2019, 11, a033928. 30670468 10.1101/cshperspect.a033928 PMC6671943

[19] N. Novo , S. Romero-Tamayo , C. Marcuello , S. Boneta , I. Blasco-Machin , A. Velázquez-Campoy , R. Villanueva , R. Moreno-Loshuertos , A. Lostao , M. Medina , P. Ferreira , PNAS Nexus 2023, 2, pgac312. 36845352 10.1093/pnasnexus/pgac312 PMC9944232

[20] Z. Su , J. G. Burchfield , P. Yang , S. J. Humphrey , G. Yang , D. Francis , S. Yasmin , S.-Y. Shin , D. M. Norris , A. L. Kearney , M. A. Astore , J. Scavuzzo , K. H. Fisher-Wellman , Q.-P. Wang , B. L. Parker , G. G. Neely , F. Vafaee , J. Chiu , R. Yeo , P. J. Hogg , D. J. Fazakerley , L. K. Nguyen , S. Kuyucak , D. E. James , Nat. Commun. 2019, 10, 5486. 31792197 10.1038/s41467-019-13114-4 PMC6889415

[21] R. Halder , D. A. Nissley , I. Sitarik , Y. Jiang , Y. Rao , Q. V. Vu , M. S. Li , J. Pritchard , E. P. O’Brien , Nat. Commun. 2023, 14, 3689. 37344452 10.1038/s41467-023-38962-z PMC10284856

[22] L. Wang , Y.-L. Yin , X.-Z. Liu , P. Shen , Y.-G. Zheng , X.-R. Lan , C.-B. Lu , J.-Z. Wang , Transl. Neurodegener. 2020, 9, 10. 32266063 10.1186/s40035-020-00189-z PMC7119290

[23] Y. Zong , H. Li , P. Liao , L. Chen , Y. Pan , Y. Zheng , C. Zhang , D. Liu , M. Zheng , J. Gao , Signal Transduction Targeted Ther. 2024, 9, 124. 10.1038/s41392-024-01839-8 PMC11094169 38744846

[24] D. M. Wilson , M. R. Cookson , L. Van Den Bosch , H. Zetterberg , D. M. Holtzman , I. Dewachter , Cell 2023, 186, 693. 36803602 10.1016/j.cell.2022.12.032

[25] J. D. Hayes , A. T. Dinkova‐Kostova , K. D. Tew , Cancer Cell 2020, 38, 167. 32649885 10.1016/j.ccell.2020.06.001 PMC7439808

[26] J. L. Silva , D. Foguel , V. F. Ferreira , T. C. R. G. Vieira , M. A. Marques , G. D. S. Ferretti , T. F. Outeiro , Y. Cordeiro , G. A. P. de Oliveira , Chem. Rev. 2023, 123, 9094. 37379327 10.1021/acs.chemrev.3c00131

[27] M. Kim , M. Mahmood , E. Reznik , P. A. Gammage , Trends in Cancer 2022, 8, 1046. 36041967 10.1016/j.trecan.2022.08.001 PMC9671861

[28] Y. M. Lee , W. He , Y.‐C. Liou , Cell Death Dis. 2021, 12, 58. 33431811 10.1038/s41419-020-03355-3 PMC7801447

[29] T. Gianferrara , E. Cescon , I. Grieco , G. Spalluto , S. Federico , Curr. Med. Chem. 2022, 29, 4631. 35170406 10.2174/0929867329666220216113517

[30] W. C. W. Chen , L. Gaidukov , Y. Lai , M.-R. Wu , J. Cao , M. J. Gutbrod , G. C. G. Choi , R. P. Utomo , Y.-C. Chen , L. Wroblewska , M. Kellis , L. Zhang , R. Weiss , T. K. Lu , Nat. Commun. 2022, 13, 6167. 36257931 10.1038/s41467-022-33287-9 PMC9579178

[31] J. Wen , L. Hong , G. Krainer , Q.-Q. Yao , T. P. J. Knowles , S. Wu , S. Perrett , J. Am. Chem. Soc. 2021, 143, 13056. 34374536 10.1021/jacs.1c03078

[32] D. I. Sideris , J. S. H. Danial , D. Emin , F. S. Ruggeri , Z. J. Xia , Y. P. Zhang , E. Lobanova , H. Dakin , S. De , A. Miller , J. C. Sang , T. P. J. Knowles , M. Vendruscolo , G. Fraser , D. Crowther , D. Klenerman , Brain Commun. 2021, 3. 10.1093/braincomms/fcab147 PMC8361392 34396107

[33] D. Emin , Y. P. Zhang , E. Lobanova , A. Miller , X. C. Li , Z. J. Xia , H. Dakin , D. I. Sideris , J. Y. L. Lam , R. T. Ranasinghe , A. Kouli , Y. Y. Zhao , S. M. De , T. P. J. Knowles , M. Vendruscolo , F. S. Ruggeri , F. I. Aigbirhio , C. H. Williams-Gray , D. Klenerman , Nat. Commun. 2022, 13. 10.1038/s41467-022-33252-6 PMC9489799 36127374

[34] M. Ziaunys , T. Sneideris , V. Smirnovas , Sci. Rep.‐Uk 2020, 10. 10.1038/s41598-020-61663-2 PMC7067779 32165692

[35] S. Qamar , G. Z. Wang , S. J. Randle , F. S. Ruggeri , J. A. Varela , J. Q. Lin , E. C. Phillips , A. Miyashita , D. Williams , F. Strohl , W. Meadows , R. Ferry , V. J. Dardov , G. G. Tartaglia , L. A. Farrer , G. S. K. Schierle , C. F. Kaminski , C. E. Holt , P. E. Fraser , G. Schmitt-Ulms , D. Klenerman , T. Knowles , M. Vendruscolo , P. St George-Hyslop , Cell 2018, 173, 720. 29677515 10.1016/j.cell.2018.03.056 PMC5927716

[36] N. Hamad , H. Watanabe , T. Uchihashi , R. Kurokawa , T. Nagata , M. Katahira , Chem. Commun. 2020, 56, 9134. 10.1039/d0cc03776a 32643734

[37] S. Vieweg , A. L. Mahul-Mellier , F. S. Ruggeri , N. Riguet , S. M. DeGuire , A. Chiki , U. Cendrowska , G. Dietler , H. A. Lashuel , J. Mol. Biol. 2021, 433. 10.1016/j.jmb.2021.167222 34492254

[38] S. De , D. C. Wirthensohn , P. Flagmeier , C. Hughes , F. A. Aprile , F. S. Ruggeri , D. R. Whiten , D. Emin , Z. J. Xia , J. A. Varela , P. Sormanni , F. Kundel , T. P. J. Knowles , C. M. Dobson , C. Bryant , M. Vendruscolo , D. Klenerman , Nat. Commun. 2019, 10. 10.1038/s41467-019-09477-3 PMC6449370 30948723

[39] C. He , T. Ahmed , A. Z. Abbasi , L. Y. Li , W. D. Foltz , P. Cai , E. Knock , P. E. Fraser , A. M. Rauth , J. T. Henderson , X. Y. Wu , Nano Today 2020, 35, 100965.

[40] E. Lobanova , D. Whiten , F. S. Ruggeri , C. G. Taylor , A. Kouli , Z. J. Xia , D. Emin , Y. P. Zhang , J. Y. L. Lam , C. H. Williams-Gray , D. Klenerman , Brain 2022, 145, 632. 34410317 10.1093/brain/awab306 PMC9014748

[41] M. Rodrigues , P. Bhattacharjee , A. Brinkmalm , D. Do , T.C. M. Pearson , S. De , A. Ponjavic , J. A. Varela , K. Kulenkampff , Baudrexel , D. Emin , F. S. Ruggeri , J. E. Lee , A. R. Carr , T. P. J. Knowles , H. Zetterberg , T. N. Snaddon , S. Gandhi , S. F. Lee , D. Klenerman , Nat. Chem. 2022, 14, 1045. 35798951 10.1038/s41557-022-00976-3

[42] L. Jönsson , A. Wimo , R. Handels , G. Johansson , M. Boada , S. Engelborghs , L. Frölich , F. Jessen , P. G. Kehoe , M. Kramberger , A. de Mendonςa , P. Ousset , J. N. Scarmeas , P. J. Visser , G. Waldemar , B. Winblad , Lancet Reg. Health Eur. 2023, 29. 10.1016/j.lanepe.2023.100657 PMC10220264 37251789

[43] K. Y. Liu , S. Walsh , C. Brayne , R. Merrick , E. Richard , R. Howard , Lancet Healthy Longevity 2023, 4, e645. 37924845 10.1016/S2666-7568(23)00193-9

[44] F. S. Ruggeri , J. Habchi , A. Cerreta , G. Dietler , Curr. Pharm. Des. 2016, 22, 3950. 27189600 10.2174/1381612822666160518141911 PMC5080865

[45] F. S. Ruggeri , T. Šneideris , M. Vendruscolo , T. P. J. Knowles , Arch. Biochem. Biophys. 2019, 664, 134. 30742801 10.1016/j.abb.2019.02.001 PMC6420408

[46] V. Yesudasu , H. S. Pradhan , R. J. Pandya , Heliyon 2021, 7. 10.1016/j.heliyon.2021.e06321 PMC8035490 33869818

[47] W. Zhang , X. Lu , J. Ren , Analyst 2025. 10.1039/d5an00021a 40195613

[48] H. Kobayashi , L.‐P. Picard , A.‐M. Schönegge , M. Bouvier , Nat. Protoc. 2019, 14, 1084. 30911173 10.1038/s41596-019-0129-7

[49] N. Kheirkhah , S. Dempsey , A. Sadeghi‐Naini , A. Samani , Med. Phys. 2023, 50, 2176. 36398744 10.1002/mp.16110

[50] G. Liu , D. Ma , H. Wang , J. Zhou , Z. Shen , Y. Yang , Y. Chen , I. Sack , J. Guo , R. Li , F. Yan , Insight Imaging 2023, 14, 89. 10.1186/s13244-023-01427-4 PMC10192481 37198348

[51] A. Diez‐Perez , M. L. Bouxsein , E. F. Eriksen , S. Khosla , J. S. Nyman , S. Papapoulos , S. Y. Tang , Bone Rep. 2016, 5, 181. 27975078 10.1016/j.bonr.2016.07.004 PMC5152622

[52] M. Dokukin , I. Dokukina , Procedia Comput. Sci. 2020, 169, 763.

[53] G. Volpe , O. M. Maragò , H. Rubinsztein-Dunlop , G. Pesce , A. B. Stilgoe , G. Volpe , G. Tkachenko , V. G. Truong , S. N. Chormaic , F. Kalantarifard , P. Elahi , M. Käll , A. Callegari , M. I. Marqués , A. A. R. Neves , W. L. Moreira , A. Fontes , C. L. Cesar , R. Saija , A. Saidi , P. Beck , J. S. Eismann , P. Banzer , T. F. D. Fernandes , F. Pedaci , W. P. Bowen , R. Vaippully , M. Lokesh , B. Roy , G. Thalhammer-Thurner , et al., J. Phys. Photonics 2023, 5, 022501.

[54] P. P. Mondal , N. Baro , A. Singh , P. Joshi , J. Basumatary , Sci. Rep.‐Uk 2022, 12, 10229. 10.1038/s41598-022-13095-3 PMC9205896 35715431

[55] S. Chakraborty , S. Haldar , Trends Biochem. Sci. 2023, 48, 740. 37246021 10.1016/j.tibs.2023.05.002

[56] C. Aermes , A. Hayn , T. Fischer , C. T. Mierke , Sci. Rep.‐Uk 2020, 10, 13453. 10.1038/s41598-020-70428-w PMC7417586 32778758

[57] D. J. Müller , A. C. Dumitru , C. Lo Giudice , H. E. Gaub , P. Hinterdorfer , G. Hummer , J. J. De Yoreo , Y. F. Dufrêne , D. Alsteens , Chem. Rev. 2021, 121, 11701. 33166471 10.1021/acs.chemrev.0c00617

[58] S. Fukuda , T. Ando , Rev. Sci. Instrum. 2021, 92, 033705. 33820001 10.1063/5.0032948

[59] R. Garcia , Chem. Soc. Rev. 2020, 49, 5850.

[60] A. Lostao , K. Lim , M. C. Pallarés , A. Ptak , C. Marcuello , Int. J. Biol. Macromol. 2023, 238, 124089. 36948336 10.1016/j.ijbiomac.2023.124089

[61] C. Marcuello , R. de Miguel , A. Lostao , Biomolecules 2022, 12, 594. 35454182 10.3390/biom12040594 PMC9024611

[62] H. Holuigue , L. Nacci , P. Di Chiaro , M. Chighizola , I. Locatelli , C. Schulte , M. Alfano , G. R. Diaferia , A. Podestà , Nanoscale 2023, 15, 15382. 37700706 10.1039/d3nr01568h

[63] J. Adamcik , C. Lara , I. Usov , J. S. Jeong , F. S. Ruggeri , G. Dietler , H. A. Lashuel , I. W. Hamley , R. Mezzenga , Nanoscale 2012, 4, 4426. 22688679 10.1039/c2nr30768e

[64] F. S. Ruggeri , S. Vieweg , U. Cendrowska , G. Longo , A. Chiki , H. A. Lashuel , G. Dietler , Sci. Rep.‐Uk 2016, 6. 10.1038/srep31155 PMC4976327 27499269

[65] D. Kurouski , A. Dazzi , R. Zenobi , A. Centrone , Chem. Soc. Rev. 2020, 49, 3315. 32424384 10.1039/c8cs00916c PMC7675782

[66] F. S. Ruggeri , B. Mannini , R. Schmid , M. Vendruscolo , T. P. J. Knowles , Nat. Commun. 2020, 11. 10.1038/s41467-020-16728-1 PMC7287102 32522983

[67] J. J. Schwartz , D. S. Jakob , A. Centrone , Chem. Soc. Rev. 2022, 51, 5248. 35616225 10.1039/d2cs00095d

[68] G. Ramer , F. S. Ruggeri , A. Levin , T. P. J. Knowles , A. Centrone , Acs Nano 2018, 12, 6612. 29932670 10.1021/acsnano.8b01425 PMC11404133

[69] F. S. Ruggeri , J. Adamcik , J. S. Jeong , H. A. Lashuel , R. Mezzenga , G. Dietler , Angew. Chem. Int. Edit. 2015, 54, 2462. 10.1002/anie.201409050 25588987

[70] F. S. Ruggeri , J. Habchi , S. Chia , R. I. Horne , M. Vendruscolo , T. P. J. Knowles , Nat. Commun. 2021, 12. 10.1038/s41467-020-20782-0 PMC7846799 33514697

[71] M. Lekka , Phil. Trans. R. Soc. A 2022, 380, 20210346. 35909354 10.1098/rsta.2021.0346

[72] A. Viljoen , M. Mathelié-Guinlet , A. Ray , N. Strohmeyer , Y. J. Oh , P. Hinterdorfer , D. J. Müller , D. Alsteens , Y. F. Dufrêne , Nat. Rev. Methods Primers 2021, 1, 63.

[73] J. Wu , M. Xiao , J. A. Quezada‐Renteria , Z. Hou , E. M. V. Hoek , J. Memb. Sci. Lett. 2024, 4, 100073.

[74] K. Lim , G. Nishide , E. S. Sajidah , T. Yamano , Y. Qiu , T. Yoshida , A. Kobayashi , M. Hazawa , T. Ando , R. Hanayama , R. W. Wong , Nano Lett. 2023, 23, 619. 36641798 10.1021/acs.nanolett.2c04270 PMC9881159

[75] A. C. Dumitru , D. Mohammed , M. Maja , J. Yang , S. Verstraeten , A. del Campo , M.-P. Mingeot-Leclercq , D. Tyteca , D. Alsteens , Adv. Sci. 2020, 7, 2002643. 10.1002/advs.202002643 PMC7675049 33240781

[76] E. Pieta , Micron 2023, 170. 10.1016/j.micron.2023.103462 37087964

[77] Y. Hou , C. Zhao , B. Xu , Y. Huang , C. Liu , Exp. Cell Res. 2021, 408, 112853. 34597679 10.1016/j.yexcr.2021.112853

[78] Y. Shen , A. Chen , W. Wang , Y. Shen , F. S. Ruggeri , S. Aime , Z. Wang , S. Qamar , J. R. Espinosa , A. Garaizar , P. St George-Hyslop , R. Collepardo-Guevara , D. A. Weitz , D. Vigolo , T. P. J. Knowles , Proc. Natl. Acad. Sci. 2023, 120. 10.1073/pnas.2301366120 PMC10438845 37549257

[79] F. S. Ruggeri , G. Longo , S. Faggiano , E. Lipiec , A. Pastore , G. Dietler , Nat. Commun. 2015, 6. 10.1038/ncomms8831 PMC4525161 26215704

[80] F. L. Lampart , R. Vetter , K. A. Yamauchi , Y. Wang , S. Runser , N. Strohmeyer , F. Meer , M.-D. Hussherr , G. Camenisch , H.-H. Seifert , C. A. Rentsch , C. Le Magnen , D. J. Müller , L. Bubendorf , D. Iber , Nat. Phys. 2025, 21, 279.

[81] A. Massey , J. Stewart , C. Smith , C. Parvini , M. McCormick , K. Do , A. X. Cartagena-Rivera , Nat. Rev. Phys. 2024, 6, 269. 38706694 10.1038/s42254-024-00707-2 PMC11066734

[82] M. Tian , Y. Li , W. Liu , L. Jin , X. Jiang , X. Wang , Z. Ding , Y. Peng , J. Zhou , J. Fan , Y. Cao , W. Wang , Y. Shi , Nanoscale 2015, 7, 12998. 26168746 10.1039/c5nr02192h

[83] S. Janel , M. Popoff , N. Barois , E. Werkmeister , S. Divoux , F. Perez , F. Lafont , Nanoscale 2019, 11, 10320. 31106790 10.1039/c8nr08955h

[84] A. Wang , K. Vijayraghavan , O. Solgaard , M. J. Butte , ACS Nano 2016, 10, 257. 26554581 10.1021/acsnano.5b03959 PMC4969083

[85] J. Zemła , J. Danilkiewicz , B. Orzechowska , J. Pabijan , S. Seweryn , M. Lekka , Semin. Cell Dev. Biol. 2018, 73, 115. 28694112 10.1016/j.semcdb.2017.06.029

[86] Y. Shen , X. Wang , J. Lu , M. Salfenmoser , N. M. Wirsik , N. Schleussner , A. Imle , A. Freire Valls , P. Radhakrishnan , J. Liang , G. Wang , T. Muley , M. Schneider , C. Ruiz de Almodovar , A. Diz-Muñoz , T. Schmidt , Cancer Cell 2020, 37, 800. 32516590 10.1016/j.ccell.2020.05.005

[87] M. J. E. Visser , E. Pretorius , Curr. Top Med. Chem. 2019, 19, 2958. 31755391 10.2174/1568026619666191121143240

[88] F. S. Ruggeri , P. Flagmeier , J. R. Kumita , G. Meisl , D. Y. Chirgadze , M. N. Bongiovanni , T. P. J. Knowles , C. M. Dobson , ACS Nano 2020, 14, 5213. 32159944 10.1021/acsnano.9b09676

[89] F. S. Ruggeri , F. Benedetti , T. P. J. Knowles , H. A. Lashuel , S. Sekatskii , G. Dietler , Proc. Natl. Acad. Sci. U.S.A. 2018, 115, 7230. 29941606 10.1073/pnas.1721220115 PMC6048494

[90] O. Khalaf , B. Fauvet , A. Oueslati , I. Dikiy , A. L. Mahul-Mellier , F. S. Ruggeri , M. K. Mbefo , F. Vercruysse , G. Dietler , S. J. Lee , D. Eliezer , H. A. Lashuel , J. Biol. Chem. 2014, 289, 21856. 24936070 10.1074/jbc.M114.553297 PMC4139205

[91] H. Konno , T. Watanabe-Nakayama , T. Uchihashi , M. Okuda , L. Zhu , N. Kodera , Y. Kikuchi , T. Ando , H. Taguchi , Proc. Natl. Acad. Sci. 2020, 117, 7831. 32213585 10.1073/pnas.1916452117 PMC7149427

[92] J. Rubin , H. Khosravi , K. L. Bruce , M. E. Lydon , S. H. Behrens , Y. O. Chernoff , A. S. Bommarius , J. Biol. Chem. 2013, 288, 30300. 23990463 10.1074/jbc.M113.467829 PMC3798495

[93] S. M. DeGuire , F. S. Ruggeri , M. B. Fares , A. Chiki , U. Cendrowska , G. Dietler , H. A. Lashuel , J. Biol. Chem. 2018, 293, 18540. 30185623 10.1074/jbc.RA118.004621 PMC6290154

[94] A. Ansaloni , Z. M. Wang , J. S. Jeong , F. S. Ruggeri , G. Dietler , H. A. Lashuel , Angew. Chem. Int. Edit. 2014, 53, 1928. 10.1002/anie.201307510 24446188

[95] T. Watanabe‐Nakayama , M. Tsuji , K. Umeda , T. Oguchi , H. Konno , M. Noguchi-Shinohara , Y. Kiuchi , N. Kodera , D. B. Teplow , K. Ono , Nano Lett. 2023, 23, 6259. 37141711 10.1021/acs.nanolett.3c00187

[96] A. Miller , S. Chia , E. Klimont , T. P. J. Knowles , M. Vendruscolo , F. S. Ruggeri , Commun. Biol. 2024, 7. 10.1038/s42003-024-05858-7 PMC10847148 38321144

[97] S. De , D. R. Whiten , F. S. Ruggeri , C. Hughes , M. Rodrigues , D. I. Sideris , C. G. Taylor , F. A. Aprile , S. Muyldermans , T. P. J. Knowles , M. Vendruscolo , C. Bryant , K. Blennow , I. Skoog , S. Kern , H. Zetterberg , D. Klenerman , Acta Neuropathol. Commun. 2019, 7, 120. 31349874 10.1186/s40478-019-0777-4 PMC6659275

[98] A. Miller , J. Wei , S. Meehan , C. M. Dobson , M. E. Welland , D. Klenerman , M. Vendruscolo , F. S. Ruggeri , T. P. J. Knowles , Proc. Natl. Acad. Sci. 2023, 120, e2216234120. 37186840 10.1073/pnas.2216234120 PMC10214208

[99] J. Zhou , F. S. Ruggeri , M. R. Zimmermann , G. Meisl , G. Longo , S. K. Sekatskii , T. P. J. Knowles , G. Dietler , Chem. Sci. 2020, 11, 3687. 34094057 10.1039/d0sc00281j PMC8152616

[100] J. Adamcik , F. S. Ruggeri , J. T. Berryman , A. Zhang , T. P. J. Knowles , R. Mezzenga , Adv. Sci. 2021, 8. 10.1002/advs.202002182 PMC7816722 33511004

[101] A. Boire , K. Burke , T. R. Cox , T. Guise , M. Jamal-Hanjani , T. Janowitz , R. Kaplan , R. Lee , C. Swanton , M. G. Vander Heiden , E. Sahai , Nat. Rev. Cancer 2024, 24, 578. 38898221 10.1038/s41568-024-00708-4 PMC7616303

[102] M. Zhivagui , A. Hoda , N. Valenzuela , Y.-Y. Yeh , J. Dai , Y. He , S. P. Nandi , B. Otlu , B. Van Houten , L. B. Alexandrov , Nat. Commun. 2023, 14, 276. 36650165 10.1038/s41467-023-35876-8 PMC9845303

[103] G. L. Brien , K. Stegmaier , S. A. Armstrong , Nat. Rev. Cancer 2019, 19, 255. 30962549 10.1038/s41568-019-0132-x

[104] K. Bera , A. Kiepas , I. Godet , Y. Li , P. Mehta , B. Ifemembi , C. D. Paul , A. Sen , S. A. Serra , K. Stoletov , J. Tao , G. Shatkin , S. J. Lee , Y. Zhang , A. Boen , P. Mistriotis , D. M. Gilkes , J. D. Lewis , C.-M. Fan , A. P. Feinberg , M. A. Valverde , S. X. Sun , K. Konstantopoulos , Nature 2022, 611, 365. 36323783 10.1038/s41586-022-05394-6 PMC9646524

[105] A. G. Casanova , G. S. Roth , S. Hausmann , X. Lu , L. J. M. Bischoff , E. M. Froeliger , L. Belmudes , E. Bourova-Flin , N. M. Flores , A. M. Benitez , T. Chasan , M. Caporicci , J. Vayr , S. Blanchet , F. Ielasi , S. Rousseaux , P. Hainaut , O. Gozani , M. Le Romancer , Y. Couté , A. Palencia , P. K. Mazur , N. Reynoird , Cell Discovery 2024, 10, 12. 38296970 10.1038/s41421-023-00644-x PMC10830559

[106] B. A. Radman , A. M. M. Alhameed , G. Shu , G. Yin , M. Wang , J. Mater. Chem. B 2024, 12, 5299. 38742281 10.1039/d4tb00328d

[107] R. Huang , P.‐K. Zhou , Signal Transduction Targeted Ther. 2021, 6, 254. 10.1038/s41392-021-00648-7 PMC8266832 34238917

[108] L. B. Alexandrov , S. Nik-Zainal , D. C. Wedge , S. A. J. R. Aparicio , S. Behjati , A. V. Biankin , G. R. Bignell , N. Bolli , A. Borg , A.-L. Børresen-Dale , S. Boyault , B. Burkhardt , A. P. Butler , C. Caldas , H. R. Davies , C. Desmedt , R. Eils , J. E. Eyfjörd , J. A. Foekens , M. Greaves , F. Hosoda , B. Hutter , T. Ilicic , S. Imbeaud , M. Imielinski , N. Jäger , D. T. W. Jones , D. Jones , S. Knappskog , M. Kool , et al., Nature 2013, 500, 415.23945592

[109] P. P. Ruvolo , Biochim. Biophys. Acta, Mol. Cell Res. 2016, 1863, 427. 10.1016/j.bbamcr.2015.08.008 26264495

[110] C.‐H. Heldin , K. Rubin , K. Pietras , A. Östman , Nat. Rev. Cancer 2004, 4, 806. 15510161 10.1038/nrc1456

[111] M. Peralta , N. Osmani , J. G. Goetz , IScience 2022, 25, 103969. 35281737 10.1016/j.isci.2022.103969 PMC8914312

[112] T. Itoh , K. Tsujita , Curr. Opin. Cell Biol. 2023, 81, 102173. 37224683 10.1016/j.ceb.2023.102173

[113] C. Alibert , B. Goud , J.‐B. Manneville , Biol. Cell 2017, 109, 167. 28244605 10.1111/boc.201600078

[114] N. Kaji , Anal. Sci. 2025, 41, 185. 39960617 10.1007/s44211-025-00714-y

[115] R. Gurrala , C. E. Byrne , L. M. Brown , R. F. P. Tiongco , M. D. Matossian , J. J. Savoie , B. M. Collins-Burow , M. E. Burow , E. C. Martin , F. H. Lau , Front. Bioeng. Biotchnol. 2021, 9. 10.3389/fbioe.2021.618448 PMC8006399 33791282

[116] R. Kaul‐Ghanekar , S. Singh , H. Mamgain , A. Jalota-Badhwar , K. M. Paknikar , S. Chattopadhyay , BMC Cancer 2009, 9, 350. 19799771 10.1186/1471-2407-9-350 PMC2765988

[117] K. Ren , J. Gao , D. Han , Front. Cell Dev. Biol. 2021, 9, 663021. 34055793 10.3389/fcell.2021.663021 PMC8152666

[118] H. Kim , K. Ishibashi , K. Matsuo , A. Kira , T. Okada , K. Watanabe , M. Inada , C. Nakamura , Anal. Chem. 2019, 91, 10557. 31322341 10.1021/acs.analchem.9b01569

[119] I. Acerbi , L. Cassereau , I. Dean , Q. Shi , A. Au , C. Park , Y. Y. Chen , J. Liphardt , E. S. Hwang , V. M. Weaver , Integr. Biol. (Camb) 2015, 7, 1120. 25959051 10.1039/c5ib00040h PMC4593730

[120] A. Levillain , C. B. Confavreux , M. Decaussin-Petrucci , E. Durieux , P. Paparel , K. Le-Bail Carval , L. Maillard , F. Bermond , D. Mitton , H. Follet , Materialia 2022, 25, 101555.

[121] P. Osmulski , D. Mahalingam , M. E. Gaczynska , J. Liu , S. Huang , A. M. Horning , C.-M. Wang , I. M. Thompson , T. H. M. Huang , C.-L. Chen , Prostate 2014, 74, 1297. 25065737 10.1002/pros.22846 PMC4142568

[122] W. Xu , R. Mezencev , B. Kim , L. Wang , J. McDonald , T. Sulchek , PLoS One 2012, 7, e46609. 23056368 10.1371/journal.pone.0046609 PMC3464294

[123] P. C. Ke , R. Zhou , L. C. Serpell , R. Riek , T. P. J. Knowles , H. A. Lashuel , E. Gazit , I. W. Hamley , T. P. Davis , M. Fandrich , D. E. Otzen , M. R. Chapman , C. M. Dobson , D. S. Eisenberg , R. Mezzenga , Chem. Soc. Rev. 2020, 49, 5473. 32632432 10.1039/c9cs00199a PMC7445747

[124] S. J. Tabrizi , M. D. Flower , C. A. Ross , E. J. Wild , Nat. Rev. Neurol. 2020, 16, 529. 32796930 10.1038/s41582-020-0389-4

[125] S. Makin , Nature 2018, 559, S4. 30046080 10.1038/d41586-018-05719-4

[126] M. Arrasate , S. Mitra , E. S. Schweitzer , M. R. Segal , S. Finkbeiner , Nature 2004, 431, 805. 15483602 10.1038/nature02998

[127] E. J. Slow , R. K. Graham , A. P. Osmand , R. S. Devon , G. Lu , Y. Deng , J. Pearson , K. Vaid , N. Bissada , R. Wetzel , B. R. Leavitt , M. R. Hayden , Proc. Natl. Acad. Sci. U.S.A. 2005, 102, 11402. 16076956 10.1073/pnas.0503634102 PMC1183566

[128] N. D. Younan , J. H. Viles , Biochemistry 2015, 54, 4297. 26087242 10.1021/acs.biochem.5b00309

[129] T. C. T. Michaels , D. Y. Qian , A. Saric , M. Vendruscolo , S. Linse , T. P. J. Knowles , Nat. Rev. Phys. 2023, 5, 379.

[130] W. P. Lipinski , B. S. Visser , I. Robu , M. A. A. Fakhree , S. Lindhoud , M. M. A. E. Claessens , E. Spruijt , Sci. Adv. 2022, 8. 10.1126/sciadv.abq6495 PMC10942789 36459561

[131] S. T. Dada , M. C. Hardenberg , Z. Toprakcioglu , L. K. Mrugalla , M. P. Cali , M. O. McKeon , E. Klimont , T. C. T. Michaels , T. P. J. Knowles , M. Vendruscolo , Proc. Natl. Acad. Sci. U.S.A. 2023, 120. 10.1073/pnas.2208792120 PMC9992821 36802433

[132] S. Wegmann , B. Eftekharzadeh , K. Tepper , K. M. Zoltowska , R. E. Bennett , S. Dujardin , P. R. Laskowski , D. MacKenzie , T. Kamath , C. Commins , C. Vanderburg , A. D. Roe , Z. Y. Fan , A. M. Molliex , A. Hernandez-Vega , D. Muller , A. A. Hyman , E. Mandelkow , J. P. Taylor , B. T. Hyman , Embo. J. 2018, 37. 10.15252/embj.201798049 PMC5881631 29472250

[133] A. Patel , H. O. Lee , L. Jawerth , S. Maharana , M. Jahnel , M. Y. Hein , S. Stoynov , J. Mahamid , S. Saha , T. M. Franzmann , A. Pozniakovski , I. Poser , N. Maghelli , L. A. Royer , M. Weigert , E. W. Myers , S. Grill , D. Drechsel , A. A. Hyman , S. Alberti , Cell 2015, 162, 1066. 26317470 10.1016/j.cell.2015.07.047

[134] A. E. Conicella , G. H. Zerze , J. Mittal , N. L. Fawzi , Structure 2016, 24, 1537. 27545621 10.1016/j.str.2016.07.007 PMC5014597

[135] M. Feric , N. Vaidya , T. S. Harmon , D. M. Mitrea , L. Zhu , T. M. Richardson , R. W. Kriwacki , R. V. Pappu , C. P. Brangwynne , Cell 2016, 165, 1686. 27212236 10.1016/j.cell.2016.04.047 PMC5127388

[136] P. G. Yang , C. Mathieu , R. M. Kolaitis , P. P. Zhang , J. Messing , U. Yurtsever , Z. M. Yang , J. J. Wu , Y. X. Li , Q. F. Pan , J. Y. Yu , E. W. Martin , T. Mittag , H. J. Kim , J. P. Taylor , Cell 2020, 181, 325. 32302571 10.1016/j.cell.2020.03.046 PMC7448383

[137] J. Söding , D. Zwicker , S. Sohrabi‐Jahromi , M. Boehning , J. Kirschbaum , Trends Cell Biol. 2020, 30, 4. 31753533 10.1016/j.tcb.2019.10.006

[138] S. Alberti , A. A. Hyman , Nat. Rev. Mol. Cell Bio. 2021, 22, 196. 33510441 10.1038/s41580-020-00326-6

[139] C. Weber , T. Michaels , L. Mahadevan , eLife 2019, 8. 10.7554/eLife.42315 PMC6516824 31084715

[140] S. Ray , N. Singh , R. Kumar , K. Patel , S. Pandey , D. Datta , J. Mahato , R. Panigrahi , A. Navalkar , S. Mehra , L. Gadhe , D. Chatterjee , A. S. Sawner , S. Maiti , S. Bhatia , J. A. Gerez , A. Chowdhury , A. Kumar , R. Padinhateeri , R. Riek , G. Krishnamoorthy , S. K. Maji , Nat. Chem. 2020, 12. 10.1038/s41557-020-0465-9 32514159

[141] Z. Toprakcioglu , A. Kamada , T. C. T. Michaels , M. Q. Xie , J. Krausser , J. P. Wei , A. Saric , M. Vendruscolo , T. P. J. Knowles , Proc. Natl. Acad. Sci. U.S.A. 2022, 119. 10.1073/pnas.2109718119 PMC9351353 35901206

[142] Y. F. Dufrêne , T. Ando , R. Garcia , D. Alsteens , D. Martinez-Martin , A. Engel , C. Gerber , D. J. Müller , Nat. Nanotechnol. 2017, 12, 295. 28383040 10.1038/nnano.2017.45

[143] D. Alsteens , D. J. Müller , Y. F. Dufrêne , Acc. Chem. Res. 2017, 50, 924. 28350161 10.1021/acs.accounts.6b00638

[144] D. P. Allison , N. P. Mortensen , C. J. Sullivan , M. J. Doktycz , WIREs Nanomed. Nanobiotechnol. 2010, 2, 618. 10.1002/wnan.104 20672388

[145] H.‐J. Butt , B. Cappella , M. Kappl , Surf. Sci. Rep. 2005, 59, 1.

[146] M. Chighizola , L. Puricelli , L. Bellon , A. Podestà , J. Mol. Recognit. 2021, 34, e2879. 33098182 10.1002/jmr.2879

[147] L. Puricelli , M. Galluzzi , C. Schulte , A. Podestà , P. Milani , Rev. Sci. Instrum. 2015, 86, 033705. 25832236 10.1063/1.4915896

[148] A. del Moral , J. C. González‐Rosillo , A. Gómez , T. Puig , X. Obradors , Ultramicroscopy 2019, 196, 186. 30439605 10.1016/j.ultramic.2018.10.014

[149] A. B. Churnside , T. T. Perkins , FEBS Lett. 2014, 588, 3621. 24801176 10.1016/j.febslet.2014.04.033

[150] J. V. Méndez‐Méndez , M. T. Alonso‐Rasgado , E. Correia Faria , E. A. Flores‐Johnson , R. D. Snook , Micron 2014, 66, 37. 25080275 10.1016/j.micron.2014.05.004

[151] I. Tessmer , P. Kaur , J. Lin , H. Wang , J. Nanobiotechnol. 2013, 11, 25. 10.1186/1477-3155-11-25 PMC3723498 23855448

[152] F. S. Ruggeri , J. Charmet , T. Kartanas , Q. Peter , S. Chia , J. Habchi , C. M. Dobson , M. Vendruscolo , T. P. J. Knowles , Nat. Commun. 2018, 9. 10.1038/s41467-018-06345-4 PMC6155325 30250131

[153] A. Miller , S. Chia , Z. Toprakcioglu , T. Hakala , R. Schmid , Y. Feng , T. Kartanas , A. Kamada , M. Vendruscolo , F. S. Ruggeri , T. P. J. Knowles , Sci. Adv. 2023, 9. 10.1126/sciadv.abq3151 PMC9839325 36638180

[154] H. Habibullah , Measurement 2020, 159, 107776.

[155] G. Dai , X. Hu , Nanomanuf. Metrol. 2022, 5, 412.

[156] S. Ito , G. Schitter , Ultramicroscopy 2018, 186, 9. 29245032 10.1016/j.ultramic.2017.12.007

[157] J. Dietrich , G. Raze , A. Deraemaeker , C. Collette , G. Kerschen , J. Vibration and Control. 2025, 31, 2624.

[158] H. Feng , A. Pang , H. Zhou , Sci. Rep. Uk 2022, 12, 10357. 10.1038/s41598-022-14332-5 PMC9209471 35725755

[159] H. Liu , Y. Li , Y. Zhang , Y. Chen , Z. Song , Z. Wang , S. Zhang , J. Qian , Micron 2018, 104, 26. 29054026 10.1016/j.micron.2017.09.009

[160] A. Farokh Payam , A. Passian , Sci. Adv. 2023, 9, eadg8292. 37379392 10.1126/sciadv.adg8292 PMC10306303

[161] S. Benaglia , C. A. Amo , R. Garcia , Nanoscale 2019, 11, 15289. 31386741 10.1039/c9nr04396a

[162] A. Chandrashekar , A. Givois , P. Belardinelli , C. L. Penning , A. M. Aragón , U. Staufer , F. Alijani , Soft Matter 2022, 18, 8748. 36349749 10.1039/d2sm00482h PMC9709660

[163] G. Bhattacharya , I. Lionadi , A. Stevenson , J. Ward , A. F. Payam , Adv. Sci. 2023, 10, 2303476. 10.1002/advs.202303476 PMC10667852 37867232

[164] I. Medalsy , U. Hensen , D. J. Muller , Angew. Chem., Int. Ed. 2011, 50, 12103. 10.1002/anie.201103991 22006839

[165] K. Sweers , K. van der Werf , M. Bennink , V. Subramaniam , Nanoscale Res. Lett. 2011, 6. 10.1186/1556-276X-6-270 PMC3211334 21711775

[166] J. Adamcik , A. Berquand , R. Mezzenga , Appl. Phys. Lett. 2011, 98.

[167] L. Li , P. Zhang , J. Li , Y. Wang , Y. Wei , J. Hu , X. Zhou , B. Xu , B. Li , Nanoscale 2019, 11, 4707. 30834915 10.1039/c8nr10354b

[168] H. Schillers , I. Medalsy , S. Hu , A. L. Slade , J. E. Shaw , J. Mol. Recognit. 2016, 29, 95. 26414320 10.1002/jmr.2510 PMC5054848

[169] G. Dai , L. Xu , K. Hahm , Meas. Sci. Technol. 2020, 31, 074011.

[170] A. A. Vekinis , V. Constantoudis , Micro Nano Eng. 2020, 8, 100067.

[171] B. Cheng , S. Yang , W. Li , S. Li , S. Shafique , D. Wu , S. Ji , Y. Sun , Z. Jiang , Microsyst. Nanoeng. 2021, 7, 1. 34721888 10.1038/s41378-021-00310-w PMC8519951

[172] B. Majhy , P. Priyadarshini , A. K. Sen , RSC Adv. 2021, 11, 15467. 35424027 10.1039/d1ra02402g PMC8698786

[173] J. Loughery , M. Cox , L. M. Smith , D. W. Meek , Nucleic Acids Res. 2014, 42, 7666. 24928858 10.1093/nar/gku501 PMC4081099

[174] M. K. Sivoňová , M. Vilčková , J. Kliment , S. Mahmood , J. Jurečeková , S. Dušenková , I. Waczulíková , P. Slezák , D. Dobrota , Biomed. Rep. 2015, 3, 707. 26405550 10.3892/br.2015.496 PMC4576486

[175] J. Dai , Y. Wang , J. Gong , Y. Yao , Colloids Surf., B 2020, 190, 110973. 10.1016/j.colsurfb.2020.110973 32199258

[176] A. Stylianou , S.‐V. Kontomaris , C. Grant , E. Alexandratou , Scanning 2019, 2019, e8452851. 10.1155/2019/6149247 PMC6537000 31217831

[177] V. Gkretsi , A. Stylianou , T. Stylianopoulos , Experiment. Cell Res. 2017, 352, 281. 10.1016/j.yexcr.2017.02.019 PMC5349498 28209486

[178] V. Gkretsi , D. P. Bogdanos , Experiment. Cell Res. 2015, 334, 219. 10.1016/j.yexcr.2015.03.002 25773778

[179] E. Nelsen , C. M. Hobson , M. E. Kern , J. P. Hsiao , E. T. O’Brien Iii , T. Watanabe , B. M. Condon , M. Boyce , S. Grinstein , K. M. Hahn , M. R. Falvo , R. Superfine , Sci. Rep.‐Uk 2020, 10, 8133. 10.1038/s41598-020-65205-8 PMC7234992 32424215

[180] M. Li , L. Liu , N. Xi , Y. Wang , X. Xiao , W. Zhang , Langmuir 2014, 30, 1609. 24495237 10.1021/la4042524

[181] S. Mylvaganam , S. A. Freeman , S. Grinstein , Curr. Biol. 2021, 31, R619. 34033794 10.1016/j.cub.2021.01.036

[182] C. Lara‐Cruz , J. E. Jiménez‐Salazar , E. Ramón‐Gallegos , P. Damian‐Matsumura , N. Batina , Int. J. Nanomed. 2016, 11, 5149. 10.2147/IJN.S108768 PMC5066869 27785020

[183] M. Petrov , I. Sokolov , Cells 2023, 12, 2536. 37947614 10.3390/cells12212536 PMC10650179

[184] S. Prasad , A. Rankine , T. Prasad , P. Song , M. E. Dokukin , N. Makarova , V. Backman , I. Sokolov , Adv. NanoBiomed Res. 2021, 1, 2000116.

[185] J. Yu , H. Gao , X. Si , H. Yang , Y. Wang , in SPIE‐CLP Conf. on Advanced Photonics, Vol. 12601, SPIE, Bellingham, Washington 2023.

[186] K. K. M. Sweers , I. M. J. Segers‐Nolten , M. L. Bennink , V. Subramaniam , Soft Matter 2012, 8, 7215.

[187] R. Limbocker , S. Chia , F. S. Ruggeri , M. Perni , R. Cascella , G. T. Heller , G. Meisl , B. Mannini , J. Habchi , T. C. T. Michaels , P. K. Challa , M. Ahn , S. T. Casford , N. Fernando , C. K. Xu , N. D. Kloss , S. I. A. Cohen , J. R. Kumita , C. Cecchi , M. Zasloff , S. Linse , T. P. J. Knowles , F. Chiti , M. Vendruscolo , C. M. Dobson , Nat. Commun. 2019, 10. 10.1038/s41467-018-07699-5 PMC6333784 30644384

[188] B. Mannini , J. Habchi , S. Chia , F. S. Ruggeri , M. Perni , T. P. J. Knowles , C. M. Dobson , M. Vendruscolo , ACS Chem. Neurosci. 2018, 9, 2959. 29986583 10.1021/acschemneuro.8b00141

[189] R. Khurana , C. Ionescu-Zanetti , M. Pope , J. Li , L. Nielson , M. Ramírez-Alvarado , L. Regan , A. L. Fink , S. A. Carter , Biophys. J. 2003, 85, 1135. 12885658 10.1016/S0006-3495(03)74550-0 PMC1303232

[190] S. E. Sanchez , D. R. Whiten , G. Meisl , F. S. Ruggeri , E. Hidari , D. Klenerman , ChemBioChem 2021, 22, 2867. 34383993 10.1002/cbic.202100285 PMC8518629

[191] P. N. Nirmalraj , T. Schneider , L. Lüder , A. Felbecker , Commun. Biol. 2023, 6, 251. 36890343 10.1038/s42003-023-04606-7 PMC9995532

[192] J. S. Jeong , A. Ansaloni , R. Mezzenga , H. A. Lashuel , G. Dietler , J. Mol. Biology. 2013, 425, 1765. 10.1016/j.jmb.2013.02.005 23415897

[193] J. Pansieri , I. A. Iashchishyn , H. Fakhouri , L. Ostojić , M. Malisauskas , G. Musteikyte , V. Smirnovas , M. M. Schneider , T. Scheidt , C. K. Xu , G. Meisl , T. P. J. Knowles , E. Gazit , R. Antoine , L. A. Morozova-Roche , Chem. Sci. 2020, 11, 7031. 34122996 10.1039/c9sc05905a PMC8159403

[194] R. Cataldi , S. Chia , K. Pisani , F. S. Ruggeri , C. K. Xu , T. Šneideris , M. Perni , S. Sarwat , P. Joshi , J. R. Kumita , S. Linse , J. Habchi , T. P. J. Knowles , B. Mannini , C. M. Dobson , M. Vendruscolo , Commun. Biol. 2021, 4. 10.1038/s42003-021-01680-7 PMC7838389 33500508

[195] S. Chia , R. L. Cataldi , F. S. Ruggeri , R. Limbocker , I. Condado-Morales , K. Pisani , A. Possenti , S. Linse , T. P. J. Knowles , J. Habchi , B. Mannini , M. Vendruscolo , ACS Chem. Neurosci. 2024, 15, 1125. 38416693 10.1021/acschemneuro.3c00718 PMC10958495

[196] A. Chiki , S. M. DeGuire , F. S. Ruggeri , D. Sanfelice , A. Ansaloni , Z. M. Wang , U. Cendrowska , R. Burai , S. Vieweg , A. Pastore , G. Dietler , H. A. Lashuel , Angew. Chem. Int. Edit. 2017, 56, 5202. 10.1002/anie.201611750 28334491

[197] E. Sanders , R. Csondor , D. Šulskis , I. Baronaitė , V. Smirnovas , L. Maheswaran , J. Horrocks , R. Munro , C. Georgiadou , I. Horvath , L. A. Morozova-Roche , P. T. F. Williamson , Int. J. Mol. Sci. 2023, 24, 13200. 37686007 10.3390/ijms241713200 PMC10488161

[198] P.‐P. Guan , L.‐L. Cao , Y. Yang , P. Wang , Front. Mol. Neurosci. 2021, 14. 10.3389/fnmol.2021.757515 PMC8674839 34924952

[199] S. S. Leal , I. Cardoso , J. S. Valentine , C. M. Gomes , J. Biol. Chem. 2013, 288, 25219. 23861388 10.1074/jbc.M113.470740 PMC3757185

[200] J. Habchi , S. Chia , C. Galvagnion , T. C. T. Michaels , M. M. J. Bellaiche , F. S. Ruggeri , M. Sanguanini , I. Idini , J. R. Kumita , E. Sparr , S. Linse , C. M. Dobson , T. P. J. Knowles , M. Vendruscolo , Nat. Chem. 2018, 10, 673. 29736006 10.1038/s41557-018-0031-x

[201] R. Tamulytė , E. Jankaitytė , Z. Toleikis , V. Smirnovas , M. Jankunec , Biochim. Biophys. Acta Biomembr. 2023, 1865, 184113. 36567033 10.1016/j.bbamem.2022.184113

[202] N. Galvanetto , M. T. Ivanović , A. Chowdhury , A. Sottini , M. F. Nüesch , D. Nettels , R. B. Best , B. Schuler , Nature 2023, 619, 876. 37468629 10.1038/s41586-023-06329-5 PMC11508043

[203] T. Ando , Curr. Opin. Chem. Biol. 2019, 51, 105. 31254806 10.1016/j.cbpa.2019.05.010

[204] S. Banerjee , Z. Sun , E. Y. Hayden , D. B. Teplow , Y. L. Lyubchenko , ACS Nano 2017, 11, 12202. 29165985 10.1021/acsnano.7b05434 PMC5752618

[205] N. Kodera , D. Noshiro , S. K. Dora , T. Mori , J. Habchi , D. Blocquel , A. Gruet , M. Dosnon , E. Salladini , C. Bignon , Y. Fujioka , T. Oda , N. N. Noda , M. Sato , M. Lotti , M. Mizuguchi , S. Longhi , T. Ando , Nat. Nanotechnol. 2021, 16, 181. 33230318 10.1038/s41565-020-00798-9

[206] G. Nishide , K. Lim , M. S. Mohamed , A. Kobayashi , M. Hazawa , T. Watanabe-Nakayama , N. Kodera , T. Ando , R. W. Wong , J. Phys. Chem. Lett. 2021, 12, 3837. 33852305 10.1021/acs.jpclett.1c00697

[207] Y. Sakiyama , A. Mazur , L. E. Kapinos , R. Y. H. Lim , Nat. Nanotechnol. 2016, 11, 719. 27136131 10.1038/nnano.2016.62

[208] R. Roy , S. Hohng , T. Ha , Nat. Methods 2008, 5, 507. 18511918 10.1038/nmeth.1208 PMC3769523

[209] H. R. Saibil , Mol. Cell 2022, 82, 274. 35063096 10.1016/j.molcel.2021.12.016

[210] P. N. Osuchowska , P. Wachulak , W. Kasprzycka , A. Nowak-Stępniowska , M. Wakuła , A. Bartnik , H. Fiedorowicz , E. A. Trafny , Int. J. Mol. Sci. 2021, 22, 7279. 34298899 10.3390/ijms22147279 PMC8306697

[211] D. Lyumkis , J. Biol. Chem. 2019, 294, 5181. 30804214 10.1074/jbc.REV118.005602 PMC6442032

[212] S. A. Kotler , J. R. Brender , S. Vivekanandan , Y. Suzuki , K. Yamamoto , M. Monette , J. Krishnamoorthy , P. Walsh , M. Cauble , M. M. B. Holl , E. N. G. Marsh , A. Ramamoorthy , Sci. Rep. 2015, 5. 10.1038/srep11811 PMC4490348 26138908

[213] W. Li , J. Wang , J. Zhang , W. Wang , Curr. Opin. Struct. Biol. 2015, 30, 25. 25523438 10.1016/j.sbi.2014.11.006

[214] M. M. Dedmon , K. Lindorff‐Larsen , J. Christodoulou , M. Vendruscolo , C. M. Dobson , J. Am. Chem. Soc. 2005, 127, 476. 15643843 10.1021/ja044834j

[215] T. Ando , S. Fukuda , K. X. Ngo , H. Flechsig , Annu. Rev. Biophys. 2024, 53, 19. 38060998 10.1146/annurev-biophys-030722-113353

[216] A. Marchesi , K. Umeda , T. Komekawa , T. Matsubara , H. Flechsig , T. Ando , S. Watanabe , N. Kodera , C. M. Franz , Sci. Rep. 2021, 11, 13003. 34155261 10.1038/s41598-021-92365-y PMC8217563

[217] G. R. Heath , E. Kots , J. L. Robertson , S. Lansky , G. Khelashvili , H. Weinstein , S. Scheuring , Nature 2021, 594, 385. 34135520 10.1038/s41586-021-03551-x PMC8697813

[218] M. S. Mohamed , A. Kobayashi , A. Taoka , T. Watanabe-Nakayama , Y. Kikuchi , M. Hazawa , T. Minamoto , Y. Fukumori , N. Kodera , T. Uchihashi , T. Ando , R. W. Wong , ACS Nano 2017, 11, 5567. 28530826 10.1021/acsnano.7b00906

[219] M. S. Mohamed , M. Hazawa , A. Kobayashi , L. Guillaud , T. Watanabe-Nakayama , M. Nakayama , H. Wang , N. Kodera , M. Oshima , T. Ando , R. W. Wong , Biomaterials 2020, 256, 120198. 32622019 10.1016/j.biomaterials.2020.120198

[220] M. Shibata , T. Uchihashi , T. Ando , R. Yasuda , Sci. Rep. 2015, 5, 8724. 25735540 10.1038/srep08724 PMC4348644

[221] B. R. Sabari , A. Dall’Agnese , A. Boija , I. A. Klein , E. L. Coffey , K. Shrinivas , B. J. Abraham , N. M. Hannett , A. V. Zamudio , J. C. Manteiga , C. H. Li , Y. E. Guo , D. S. Day , J. Schuijers , E. Vasile , S. Malik , D. Hnisz , T. I. Lee , II Cisse , R. G. Roeder , P. A. Sharp , A. K. Chakraborty , R. A. Young , Science 2018, 361. 10.1126/science.aar3958 PMC6092193 29930091

[222] K. Matsumoto , D. K. Ikliptikawati , K. Makiyama , K. Mochizuki , M. Tobita , I. Kobayashi , D. C. Voon , K. Lim , K. Ogawa , I. Kashiwakura , H. I. Suzuki , H. Yoshino , R. W. Wong , M. Hazawa , J. Radiat. Res. 2024, 65, 482. 38874522 10.1093/jrr/rrae044 PMC11262858

[223] A. Elosegui‐Artola , I. Andreu , A. E. M. Beedle , A. Lezamiz , M. Uroz , A. J. Kosmalska , R. Oria , J. Z. Kechagia , P. Rico-Lastres , A.-L. L. Roux , C. M. Shanahan , X. Trepat , D. Navajas , S. Garcia-Manyes , P. Roca-Cusachs , Cell 2017, 171, 1397. 29107331 10.1016/j.cell.2017.10.008

[224] M. Hazawa , D. K. Ikliptikawati , Y. Iwashima , D.-C. Lin , Y. Jiang , Y. Qiu , K. Makiyama , K. Matsumoto , A. Kobayashi , G. Nishide , L. Keesiang , H. Yoshino , T. Minamoto , T. Suzuki , I. Kobayashi , M. Meguro-Horike , Y.-Y. Jiang , T. Nishiuchi , H. Konno , H. P. Koeffler , K. Hosomichi , A. Tajima , S.-i. Horike , R. W. Wong , Cell Chem. Biol. 2023, 5. 10.1016/j.chembiol.2023.10.005 37924814

[225] T. Watanabe‐Nakayama , K. Ono , M. Itami , R. Takahashi , D. B. Teplow , M. Yamada , Proc. Natl. Acad. Sci. 2016, 113, 5835. 27162352 10.1073/pnas.1524807113 PMC4889376

[226] T. Watanabe‐Nakayama , M. Nawa , H. Konno , N. Kodera , T. Ando , D. B. Teplow , K. Ono , ACS Nano 2020, 14, 9979. 32678577 10.1021/acsnano.0c03074

[227] H. E. Davis , L. McCorkell , J. M. Vogel , E. J. Topol , Nat. Rev. Microbiol. 2023, 21, 133. 36639608 10.1038/s41579-022-00846-2 PMC9839201

[228] G. Nishide , K. Lim , M. Tamura , A. Kobayashi , Q. Zhao , M. Hazawa , T. Ando , N. Nishida , R. W. Wong , J. Phys. Chem. Lett. 2023, 14, 8385. 37707320 10.1021/acs.jpclett.3c01440 PMC10544025

[229] A. Franco , P. Gracia , A. Colom , J. D. Camino , J. Á. Fernández-Higuero , N. Orozco , A. Dulebo , L. Saiz , N. Cremades , J. M. G. Vilar , A. Prado , A. Muga , Proc. Natl. Acad. Sci. 2021, 118, e2105548118. 34462355 10.1073/pnas.2105548118 PMC8433526

[230] D. G. Cheirdaris , Adv. Exp. Med. Biol. 2021, 1339, 187. 35023106 10.1007/978-3-030-78787-5_24

[231] L. Medina , B. Kaehr , R. E. Serda , Methods Mol. Biol. 2024, 2720, 209. 37775668 10.1007/978-1-0716-3469-1_15

[232] N. Gao , Z. Liu , H. Zhang , C. Liu , D. Yu , J. Ren , X. Qu , Angew. Chem., Int. Ed. 2022, 61, e202115336. 10.1002/anie.202115336 35137505

[233] C. Marcuello , R. de Miguel , C. Gómez‐Moreno , M. Martínez‐Júlvez , A. Lostao , Protein Eng. Des. Sel. 2012, 25, 715. 23081837 10.1093/protein/gzs086

[234] C. Marcuello , L. Foulon , B. Chabbert , M. Molinari , V. Aguié‐Béghin , Langmuir 2018, 34, 9376. 30037232 10.1021/acs.langmuir.8b01892

[235] E. T. Hennessy , M. D. Palkowitz , J. Saurí , A. C. Sather , Org. Lett. 2022, 24, 6331. 36001635 10.1021/acs.orglett.2c02555

[236] S. Pérez‐Domínguez , S. Caballero-Mancebo , C. Marcuello , M. Martínez-Júlvez , M. Medina , A. Lostao , Antioxidants 2022, 11, 537. 35326186 10.3390/antiox11030537 PMC8944804

[237] T. Ichikawa , D. Wang , K. Miyazawa , K. Miyata , M. Oshima , T. Fukuma , Commun. Biol. 2022, 5, 1. 35595960 10.1038/s42003-022-03437-2 PMC9122943

[238] C. Lo Giudice , A. C. Dumitru , D. Alsteens , Anal. Bioanal. Chem. 2019, 411, 6549. 31410537 10.1007/s00216-019-02077-6

[239] A. Fiasconaro , F. Falo , Phys. Rev. E 2023, 107, 024501. 36932488 10.1103/PhysRevE.107.024501

[240] S. Moreno‐Flores , Microsc. Res. Tech. 2016, 79, 1045. 27488016 10.1002/jemt.22742

[241] Z. Xu , Q. Li , Y. Huang , K. Guo , B. Xue , Y. Cao , Y. Li , Int. J. Mol. Sci. 2023, 24, 12414. 37569789 10.3390/ijms241512414 PMC10419274

[242] O. H. Olubowale , S. Biswas , G. Azom , B. L. Prather , S. D. Owoso , K. C. Rinee , K. Marroquin , K. A. Gates , M. B. Chambers , A. Xu , J. C. Garno , ACS Omega 2021, 6, 25860. 34660949 10.1021/acsomega.1c03829 PMC8515370

[243] A. Ebner , F. Kienberger , G. Kada , C. M. Stroh , M. Geretschläger , A. S. M. Kamruzzahan , L. Wildling , W. T. Johnson , B. Ashcroft , J. Nelson , S. M. Lindsay , H. J. Gruber , P. Hinterdorfer , ChemPhysChem 2005, 6, 897. 15884073 10.1002/cphc.200400545

[244] F. Kienberger , A. Ebner , H. J. Gruber , P. Hinterdorfer , Acc. Chem. Res. 2006, 39, 29. 16411737 10.1021/ar050084m

[245] L. A. Chtcheglova , P. Hinterdorfer , Semin. Cell Dev. Biol. 2018, 73, 45. 28807883 10.1016/j.semcdb.2017.08.025

[246] S. Senapati , S. Lindsay , Acc. Chem. Res. 2016, 49, 503. 26934674 10.1021/acs.accounts.5b00533

[247] L. R. J. Scarratt , K. Kubiak , P. Maroni , G. Trefalt , M. Borkovec , Langmuir 2020, 36, 14443. 33202133 10.1021/acs.langmuir.0c02917

[248] E. Evans , K. Ritchie , Biophys. J. 1997, 72, 1541. 9083660 10.1016/S0006-3495(97)78802-7 PMC1184350

[249] O. K. Dudko , G. Hummer , A. Szabo , Proc. Natl. Acad. Sci. 2008, 105, 15755. 18852468 10.1073/pnas.0806085105 PMC2572921

[250] R. Tapia‐Rojo , C. Marcuello , A. Lostao , C. Gómez-Moreno , J. J. Mazo , F. Falo , Phys. Chem. Chem. Phys. 2017, 19, 4567. 28124058 10.1039/c6cp07508h

[251] C. Jarzynski , Phys. Rev. Lett. 1997, 78, 2690.

[252] X. Qi , S. Lin , M. Li , Nanoscale 2025, 17, 4695. 39865849 10.1039/d4nr04033c

[253] S. Li , C. Sampson , C. Liu , H.‐l. Piao , H.‐X. Liu , Cell Commun. Signaling 2023, 21, 266. 10.1186/s12964-023-01264-4 PMC10537162 37770930

[254] Z. Li , C. Sampson , C. Liu , H.-l. Piao , H.-X. Liu , Nanoscale 2017, 9, 19039.29188243

[255] G. Caligiuri , D. A. Tuveson , Cancer Cell 2023, 41, 434. 36917949 10.1016/j.ccell.2023.02.015 PMC11022589

[256] V. M. Laurent , A. Duperray , V. S. Rajan , C. Verdier , Plos One 2014, 9, e98034. 24857933 10.1371/journal.pone.0098034 PMC4032264

[257] L. Xie , Z. Sun , Z. Hong , N. J. Brown , O. V. Glinskii , K. Rittenhouse-Olson , G. A. Meininger , V. V. Glinsky , PLoS One 2018, 13, e0204418. 30235349 10.1371/journal.pone.0204418 PMC6147572

[258] L. Xiao , Q. Chen , Y. Wu , X. Qi , A. Zhou , Biochim. Biophys. Acta 2015, 1848, 1988. 26002322 10.1016/j.bbamem.2015.05.007

[259] L. Goicoechea , F. Arenas , F. Castro , S. Nuñez , S. Torres , C. Garcia-Ruiz , J. C. Fernandez-Checa , STAR Protocols 2022, 3, 101068. 35024626 10.1016/j.xpro.2021.101068 PMC8728529

[260] H. Kim , K. Ishibashi , M. Iijima , S. Kuroda , I. C. Nakamura , Sensors 2020, 20, 5723. 33050090 10.3390/s20195723 PMC7582537

[261] T. Shi , S. Zhu , H. Guo , X. Li , S. Zhao , Y. Wang , X. Lei , D. Huang , L. Peng , Z. Li , S. Xu , Front. Oncol. 2021, 11, 567978. 33708622 10.3389/fonc.2021.567978 PMC7940546

[262] D. P. Carbone , M. Reck , L. Paz-Ares , B. Creelan , L. Horn , M. Steins , E. Felip , M. M. van den Heuvel , T.-E. Ciuleanu , F. Badin , N. Ready , T. J. N. Hiltermann , S. Nair , R. Juergens , S. Peters , E. Minenza , J. M. Wrangle , D. Rodriguez-Abreu , H. Borghaei , G. R. Blumenschein , L. C. Villaruz , L. Havel , J. Krejci , J. Corral Jaime , H. Chang , W. J. Geese , P. Bhagavatheeswaran , A. C. Chen , M. A. Socinski , N. Engl. J. Med. 2017, 376, 2415. 28636851 10.1056/NEJMoa1613493 PMC6487310

[263] F. T. Hane , R. Hayes , B. Y. Lee , Z. Leonenko , PLoS One 2016, 11, e0147488. 26808970 10.1371/journal.pone.0147488 PMC4726707

[264] Z. Lv , R. Roychaudhuri , M. M. Condron , D. B. Teplow , Y. L. Lyubchenko , Sci. Rep. 2013, 3, 2880. 24096987 10.1038/srep02880 PMC3791449

[265] G. Bitan , M. D. Kirkitadze , A. Lomakin , S. S. Vollers , G. B. Benedek , D. B. Teplow , Proc. Natl. Acad. Sci. 2003, 100, 330. 12506200 10.1073/pnas.222681699 PMC140968

[266] S. Maity , E. Viazovkina , A. Gall , Y. L. Lyubchenko , Phys. Chem. Chem. Phys. 2017, 19, 16387. 28621364 10.1039/c7cp02691a PMC5536842

[267] E. Y. Choi , S. S. Kang , S. K. Lee , B. H. Han , Biomol. Ther. Seoul 2020, 28, 145. 31697876 10.4062/biomolther.2019.113 PMC7059817

[268] H. Frentrup , M. S. Allen , Nanotechnology 2011, 22, 295703. 21673383 10.1088/0957-4484/22/29/295703

[269] H. Schillers , C. Rianna , J. Schäpe , T. Luque , H. Doschke , M. Wälte , J. J. Uriarte , N. Campillo , G. P. A. Michanetzis , J. Bobrowska , A. Dumitru , E. T. Herruzo , S. Bovio , P. Parot , M. Galluzzi , A. Podestà , L. Puricelli , S. Scheuring , Y. Missirlis , R. Garcia , M. Odorico , J.-M. Teulon , F. Lafont , M. Lekka , F. Rico , A. Rigato , J.-L. Pellequer , H. Oberleithner , D. Navajas , M. Radmacher , Sci. Rep. 2017, 7, 5117. 28698636 10.1038/s41598-017-05383-0 PMC5505948

[270] F. Rico , P. Roca-Cusachs , N. Gavara , R. Farré , M. Rotger , D. Navajas , Phys. Rev. E Stat. Nonlin. Soft Matter. Phys. 2005, 72, 021914. 16196611 10.1103/PhysRevE.72.021914

[271] S. V. Kontomaris , A. Malamou , A. Stylianou , Micron. 2022, 155, 103228. 35124406 10.1016/j.micron.2022.103228

[272] I. N. Sneddon , Int. J. Eng. Sci. 1965, 3, 47.

[273] J. Shen , D. Zhang , F.‐H. Zhang , Y. Gan , Appl. Surf. Sci. 2017, 422, 482.

[274] B. V. Derjaguin , V. M. Muller , Y. P. Toporov , J. Colloid Interface Sci. 1975, 53, 314.

[275] K. L. Johnson , K. Kendall , A. D. Roberts , D. Tabor , Proc. R. Soc. London, Ser. A 1997, 324, 301.

[276] P. M. Theiler , C. Ritz , A. Stemmer , J. Appl. Phys. 2021, 130, 244304.

[277] B. Cappella , G. Dietler , Surf. Sci. Rep. 1999, 34, 1.

[278] M. LeClaire , J. Gimzewski , S. Sharma , Nano Select 2021, 2, 1.

[279] A. Nonn , B. Kiss , W. Pezeshkian , T. Tancogne-Dejean , A. Cerrone , M. Kellermayer , Y. Bai , W. Li , T. Wierzbicki , J. Mech. Behav. Biomed. Mater. 2023, 148, 106153. 37865016 10.1016/j.jmbbm.2023.106153

[280] A. Božič , A. Šiber , Proc. Natl. Acad. Sci. 2020, 117, 26600. 33028678 10.1073/pnas.2011084117 PMC7604445

[281] Y. Javanmardi , H. Colin‐York , N. Szita , M. Fritzsche , E. Moeendarbary , Commun. Phys. 2021, 4, 1. 10.1038/s42005-021-00740-y PMC7612038 34841089

[282] R. H. Pritchard , P. Lava , D. Debruyne , E. M. Terentjev , Soft Matter. 2013, 9, 6037.

[283] S. Chiodini , S. Ruiz-Rincón , P. D. Garcia , S. Martin , K. Kettelhoit , I. Armenia , D. B. Werz , P. Cea , Small 2020, 16, 2000269. 10.1002/smll.202000269 32761794

[284] O. Goodman , B. Derby , Acta Mater. 2011, 59, 1790.

[285] A. Castellanos‐Gomez , M. Poot , A. Amor-Amorós , G. A. Steele , H. S. J. van der Zant , N. Agraït , G. Rubio-Bollinger , Nano Res. 2012, 5, 550. 10.1186/1556-276X-7-233 PMC3359267 22533903

[286] P. D. Garcia , R. Garcia , Biophys. J. 2018, 114, 2923. 29925028 10.1016/j.bpj.2018.05.012 PMC6026379

[287] B. L. Doss , M. Pan , M. Gupta , G. Grenci , R.-M. Mège , C. T. Lim , M. P. Sheetz , R. Voituriez , B. Ladoux , Proc. Natl. Acad. Sci. 2020, 117, 12817. 32444491 10.1073/pnas.1917555117 PMC7293595

[288] D. Pérez‐Calixto , S. Amat-Shapiro , D. Zamarrón-Hernández , G. Vázquez-Victorio , P.-H. Puech , M. Hautefeuille , Polymers 2021, 13, 629. 33672475 10.3390/polym13040629 PMC7923444

[289] T. J. Paul , Z. Hoffmann , C. Wang , M. Shanmugasundaram , J. DeJoannis , A. Shekhtman , I. K. Lednev , V. K. Yadavalli , R. Prabhakar , J. Phys. Chem. Lett. 2016, 7, 2758. 27387853 10.1021/acs.jpclett.6b01066 PMC5956519

[290] J. Peng , J. Guo , R. Ma , Y. Jiang , Surf. Sci. Rep. 2022, 77, 100549.

[291] J. K. Wychowaniec , J. Moffat , A. Saiani , J. Mech. Behav. Biomed. Mater. 2021, 124, 104776. 34479107 10.1016/j.jmbbm.2021.104776

[292] S. Abuhattum , D. Mokbel , P. Müller , D. Soteriou , J. Guck , S. Aland , IScience 2022, 25. 10.1016/j.isci.2022.104016 PMC8931349 35310950

[293] S. Moreno‐Flores , R. Benitez , M. D. Vivanco , J. L. Toca‐Herrera , J. Biomech. 2010, 43, 349. 19772964 10.1016/j.jbiomech.2009.07.037

[294] D.‐L. Chen , P.‐F. Yang , Y.‐S. Lai , Microelectron. Reliab. 2012, 52, 541.

[295] J. H. Kim , D. Yang , S. Park , J. Biomech. 2024, 162, 111908. 38142667 10.1016/j.jbiomech.2023.111908 PMC10842778

[296] A. Weber , D. Tyrakowski , J. L. Toca‐Herrera , Langmuir 2022, 38, 15552. 36484724 10.1021/acs.langmuir.2c02172 PMC9776528

[297] A. Raman , S. Trigueros , A. Cartagena , A. P. Z. Stevenson , M. Susilo , E. Nauman , S. A. Contera , Nat. Nanotechnol. 2011, 6, 809. 22081213 10.1038/nnano.2011.186

[298] W. Fan , K. Adebowale , L. Váncza , Y. Li , M. F. Rabbi , K. Kunimoto , D. Chen , G. Mozes , D. K.-C. Chiu , Y. Li , J. Tao , Y. Wei , N. Adeniji , R. L. Brunsing , R. Dhanasekaran , A. Singhi , D. Geller , S. H. Lo , L. Hodgson , E. G. Engleman , G. W. Charville , V. Charu , S. P. Monga , T. Kim , R. G. Wells , O. Chaudhuri , N. J. Török , Nature 2024, 626, 635. 38297127 10.1038/s41586-023-06991-9 PMC10866704

[299] J. Rother , H. Nöding , I. Mey , A. Janshoff , Open Biol. 2014, 4, 140046. 24850913 10.1098/rsob.140046 PMC4042852

[300] I. Rezaei , A. Sadeghi , Biochem. Cell Biol. 2023, 101, 531. 37437307 10.1139/bcb-2022-0322

[301] M. Cieśluk , K. Pogoda , P. Deptuła , P. Werel , A. Kułakowska , J. Kochanowicz , Z. Mariak , T. Łysoń , J. Reszeć , R. Bucki , Int. J. Nanomed. 2020, 15, 7509. 10.2147/IJN.S270147 PMC7547774 33116485

[302] A. P. Rickel , H. J. Sanyour , N. A. Leyda , Z. Hong , ACS Appl. Bio Mater. 2020, 3, 2360. 10.1021/acsabm.0c00100 PMC8318011 34327310

[303] A. V. Vakhrusheva , A. V. Murashko , E. S. Trifonova , Y. M. Efremov , P. S. Timashev , O. S. Sokolova , Eur. J. Cell Biol. 2022, 101, 151241. 35653881 10.1016/j.ejcb.2022.151241

[304] A. Amiri , C. Dietz , A. Rapp , M. C. Cardoso , R. W. Stark , Nanoscale 2023, 15, 15008. 37668423 10.1039/d3nr02226a

[305] S. G. Kulkarni , S. Pérez‐Domínguez , M. Radmacher , J. Mol. Recognit. 2023, 36, e3018. 37025035 10.1002/jmr.3018

[306] M. Dessard , J.‐B. Manneville , J.‐F. Berret , Nanoscale Adv. 2024, 6, 1727. 38482035 10.1039/d4na00003j PMC10929591

[307] A. I. Alalawy , Cancer Cell Int. 2024, 24, 244. 39003454 10.1186/s12935-024-03415-0 PMC11245874

[308] A. Weber , M. Vivanco , D. J. L. Toca‐Herrera , Sci. Rep. Uk 2023, 13, 3087. 10.1038/s41598-023-30156-3 PMC9947176 36813800

[309] A. Botet‐Carreras , et al., Colloids Surf., B 2023, 221, 112968. 10.1016/j.colsurfb.2022.112968 36335823

[310] V. Y. Bairamukov , A. S. Bukatin , R. A. Kamyshinsky , V. S. Burdakov , E. B. Pichkur , T. A. Shtam , M. N. Starodubtseva , Biochim. Biophys. Acta, Gen. Subj. 2022, 1866, 130139. 35390487 10.1016/j.bbagen.2022.130139

[311] C. Gabbutt , W. Shen , J. Seifert , S. Contera , Sci. Rep. Uk 2019, 9, 19473. 10.1038/s41598-019-55519-7 PMC6923397 31857622

[312] M. C. Piontek , R. B. Lira , W. H. Roos , Biochim. Biophys. Acta, Gen. Subj. 2021, 1865, 129486. 31734458 10.1016/j.bbagen.2019.129486

[313] H. P. Wampler , A. Ivanisevic , Micron 2009, 40, 444. 19286387 10.1016/j.micron.2009.01.002

[314] P. Lemoine , C. Dooley , A. Morelli , E. Harrison , D. Dixon , Appl. Surf. Sci. 2022, 574, 151386.

[315] Q. Gao , Y. Fang , S. Zhang , H. S. H. Wong , Y. E. Chan , S. S. M. Wong , K. K. L. Yung , K. W. C. Lai , J. Biomech. 2019, 86, 79. 30770196 10.1016/j.jbiomech.2019.01.046

[316] A. A. Ungureanu , I. Benilova , O. Krylychkina , D. Braeken , B. De Strooper , C. Van Haesendonck , C. G. Dotti , C. Bartic , Sci. Rep. 2016, 6, 25841. 27173984 10.1038/srep25841 PMC4865860

[317] E. Lipiec , F. S. Ruggeri , C. Benadiba , A. M. Borkowska , J. D. Kobierski , J. Miszczyk , B. R. Wood , G. B. Deacon , A. Kulik , G. Dietler , W. M. Kwiatek , Nucleic Acids Res. 2019, 47. 10.1093/nar/gkz630 PMC6765102 31562528

[318] D. E. Otzen , M. S. Dueholm , Z. Najarzadeh , T. P. J. Knowles , F. S. Ruggeri , Small Methods 2021, 5. 10.1002/smtd.202001002 34927901

[319] T. Dou , Z. Li , J. Zhang , A. Evilevitch , D. Kurouski , Anal. Chem. 2020, 92, 11297. 32683857 10.1021/acs.analchem.0c01971

[320] S. Y. Kim , D. Khanal , B. Kalionis , W. Chrzanowski , Nat. Protoc. 2019, 14, 576. 30651586 10.1038/s41596-018-0109-3

[321] F. S. Ruggeri , C. Marcott , S. Dinarelli , G. Longo , M. Girasole , G. Dietler , T. P. J. Knowles , Int. J. Mol. Sci. 2018, 19. 10.3390/ijms19092582 PMC6163177 30200270

[322] S. Kenkel , M. Gryka , L. Chen , M. P. Confer , A. Rao , S. Robinson , K. V. Prasanth , R. Bhargava , Proc. Natl. Acad. Sci. U.S.A. 2022, 119. 10.1073/pnas.2210516119 PMC9704695 36375054

[323] R. Chikkaraddy , V. A. Turek , N. Kongsuwan , F. Benz , C. Carnegie , T. van de Goor , B. de Nijs , A. Demetriadou , O. Hess , U. F. Keyser , J. J. Baumberg , Nano Lett. 2018, 18, 405. 29166033 10.1021/acs.nanolett.7b04283 PMC5806994

[324] X. Chen , P. Liu , Z. Hu , L. Jensen , Nat. Commun. 2019, 10, 2567. 31189893 10.1038/s41467-019-10618-x PMC6561954

[325] C. Blum , T. Schmid , L. Opilik , S. Weidmann , S. R. Fagerer , R. Zenobi , J. Raman Spectrosc. 2012, 43, 1895.

[326] X. Li , S. P. Pujari , J. van der Gucht , H. Zuilhof , F. S. Ruggeri , Nat. Commun. 2025, 16, 6761. 40695796 10.1038/s41467-025-62041-0 PMC12284071

[327] A. Dazzi , F. Glotin , R. Carminati , J. Appl. Phys. 2010, 107.

[328] G. Ramer , V. A. Aksyuk , A. Centrone , Anal. Chem. 2017, 89, 13524. 29165992 10.1021/acs.analchem.7b03878 PMC5841475

[329] B. Lahiri , G. Holland , A. Centrone , Small 2013, 9, 439. 23034929 10.1002/smll.201200788

[330] A. Dazzi , R. Prazeres , E. Glotin , J. M. Ortega , Opt. Lett. 2005, 30, 2388. 16196328 10.1364/ol.30.002388

[331] A. C. VD Dos Santos , N. Hondl , V. Ramos-Garcia , J. Kuligowski , B. Lendl , G. Ramer , ACS Meas. Sci. Au 2023. 10.1021/acsmeasuresciau.3c00010 PMC10588935 37868358

[332] F. Lu , M. A. Belkin , Opt Express 2011, 19, 19942. 21997003 10.1364/OE.19.019942

[333] F. Lu , M. Jin , M. A. Belkin , Nat. Photon. 2014, 8, 307.

[334] M. Jin , F. Lu , M. A. Belkin , Light:Sci. Appl. 2017, 6, e17096. 30167276 10.1038/lsa.2017.96 PMC6062223

[335] N. Piergies , M. Oćwieja , J. Maciejewska-Prończuk , R. Kosydar , C. Paluszkiewicz , W. M. Kwiatek , Nanoscale 2023, 15, 11693. 37387227 10.1039/d3nr01218b

[336] P. Henrot , A. Leroux , C. Barlier , P. Génin , Diagn. Interv. Imag 2014, 95, 141. 10.1016/j.diii.2013.12.011 24525087

[337] M. Petay , M. Cherfan , E. Bouderlique , S. Reguer , J. Mathurin , A. Dazzi , M. L’Heronde , M. Daudon , E. Letavernier , A. Deniset-Besseau , D. Bazin , Cr. Chim. 2022, 25, 553.

[338] C. Paluszkiewicz , N. Piergies , M. C. Guidi , E. Pięta , W. Ścierski , M. Misiołek , B. Drozdzowska , P. Ziora , G. Lisowska , W. M. Kwiatek , Biochim. Biophys. Acta, Gen. Subj. 2020, 1864, 129677. 32634535 10.1016/j.bbagen.2020.129677

[339] E. Kennedy , R. Al‐Majmaie , M. Al‐Rubeai , D. Zerulla , J. H. Rice , J. Biophoton. 2015, 8, 133. 10.1002/jbio.201300138 24307406

[340] M. Roman , T. P. Wrobel , A. Panek , C. Paluszkiewicz , W. M. Kwiatek , Nanotechnology 2019, 30. 10.1088/1361-6528/ab31dd 31300624

[341] M. Roman , T. P. Wrobel , C. Paluszkiewicz , W. M. Kwiatek , J. Biophotonics 2020, 13. 10.1002/jbio.201960094 31999078

[342] C. Policar , J. B. Waern , M. A. Plamont , S. Clède , C. Mayet , R. Prazeres , J. M. Ortega , A. Vessières , A. Dazzi , Angew. Chem. Int. Edit 2011, 50, 860. 10.1002/anie.201003161 20941714

[343] S. Clède , F. Lambert , C. Sandt , S. Kascakova , M. Unger , E. Harté , M. A. Plamont , R. Saint-Fort , A. Deniset-Besseau , Z. Gueroui , C. Hirschmugl , S. Lecomte , A. Dazzi , A. Vessières , C. Policar , Analyst 2013, 138, 5627. 23897394 10.1039/c3an00807j

[344] M. Schulz , W. H. Binder , Macromol. Rapid Comm. 2015, 36, 2031. 10.1002/marc.201500344 26457675

[345] M. Kang , M. Tuteja , A. Centrone , D. Topgaard , C. Leal , Adv. Funct. Mater. 2018, 28. 10.1002/adfm.201704356 PMC6508631 31080383

[346] M. Tuteja , M. Kang , C. Leal , A. Centrone , Analyst 2018, 143, 3808. 29878001 10.1039/c8an00838h PMC6215448

[347] N. Piergies , J. Mathurin , A. Dazzi , A. Deniset-Besseau , M. Oćwieja , C. Paluszkiewicz , W. M. Kwiatek , Appl. Surf. Sci. 2023, 609, 155217.

[348] N. Piergies , A. Dazzi , A. Deniset-Besseau , J. Mathurin , M. Ocwieja , C. Paluszkiewicz , W. M. Kwiatek , Nano Res. 2020, 13, 1020.

[349] Y. Wang , F. Heinemann , S. Top , A. Dazzi , C. Policar , L. Henry , F. Lambert , G. Jaouen , M. Salmain , A. Vessieres , Dalton T. 2018, 47, 9824. 10.1039/c8dt01582a 29993046

[350] A. Nagalingam , P. Kuppusamy , S. V. Singh , D. Sharma , N. K. Saxena , Cancer Res. 2014, 74, 2617. 24732433 10.1158/0008-5472.CAN-13-2081 PMC4009451

[351] D. Galante , F. S. Ruggeri , G. Dietler , F. Pellistri , E. Gatta , A. Corsaro , T. Florio , A. Perico , C. D’Arrigo , Int. J. Biochem. Cell Biol. 2016, 79, 261. 27592450 10.1016/j.biocel.2016.08.037

[352] F. S. Ruggeri , A. M. Miller , M. Vendruscolo , T. P. J. Knowles , Nanoscale Infrared Vib. Spectrosc. Bio‐Protoc. 2021, 11. 10.21769/BioProtoc.4122 PMC8413583 34541041

[353] Y. Shen , F. S. Ruggeri , D. Vigolo , A. Kamada , S. Qamar , A. Levin , C. Iserman , S. Alberti , P. S. George-Hyslop , T. P. J. Knowles , Nat. Nanotechnol. 2020, 15, 841. 32661370 10.1038/s41565-020-0731-4 PMC7116851

[354] L. Zhou , D. Kurouski , Anal. Chem. 2020, 92, 6806. 32347706 10.1021/acs.analchem.0c00593

[355] M. Matveyenka , A. Ali , C. L. Mitchell , H. C. Brown , D. Kurouski , ACS Chem. Neurosci. 2024, 15, 4075. 39469734 10.1021/acschemneuro.4c00501 PMC11587506

[356] K. Zhaliazka , A. Ali , D. Kurouski , ACS Chem. Neurosci. 2024, 15, 371. 38166409 10.1021/acschemneuro.3c00671 PMC12152213

[357] K. Zhaliazka , D. Kurouski , ACS Chem. Neurosci. 2024, 15, 3344. 39222387 10.1021/acschemneuro.4c00275 PMC11413849

[358] T. Dou , D. Kurouski , ACS Chem. Neurosci. 2022, 13, 2380. 35904551 10.1021/acschemneuro.2c00355 PMC10405296

[359] T. Dou , L. Zhou , D. Kurouski , J. Phys. Chem. Lett. 2021, 12, 4407. 33945282 10.1021/acs.jpclett.1c00820

[360] K. Zhaliazka , D. Kurouski , Protein Sci. 2023, 32, e4598. 36823759 10.1002/pro.4598 PMC10019452

[361] S. Banerjee , B. Holcombe , S. Ringold , A. Foes , T. Naik , D. Baghel , A. Ghosh , J. Phys. Chem. B 2022, 126, 5832. 35914320 10.1021/acs.jpcb.2c04797 PMC9612939

[362] S. Banerjee , T. Naik , D. Baghel , A. Ghosh , J. Phys. Chem. B 2023, 127, 5799. 37363988 10.1021/acs.jpcb.3c01869 PMC10691422

[363] Y. Shen , T. Schmidt , A. Diz‐Muñoz , STAR Protoco. 2020, 1, 100167. 10.1016/j.xpro.2020.100167 PMC7757366 33377061

[364] M. Shimizu , C. Okamoto , K. Umeda , S. Watanabe , T. Ando , N. Kodera , Rev. Sci. Instrum. 2022, 93, 013701. 35104993 10.1063/5.0072722

[365] T. Zhang , H. Yu , J. Shi , X. Wang , H. Luo , D. Lin , Z. Liu , C. Su , Y. Wang , L. Liu , Adv. Sci. 2022, 9, 2103902. 10.1002/advs.202103902 PMC9036010 35224895

[366] S. Salucci , M. Battistelli , S. Burattini , F. Sbrana , E. Falcieri , Microsc. Res. Tech. 2020, 83, 1464. 32681811 10.1002/jemt.23539

[367] V. Novotna , J. Horak , M. Konecny , V. Hegrova , O. Novotny , Z. Novacek , J. Neuman , Microsc. Today 2020, 28, 38.

[368] P. Beekman , A. Enciso-Martinez , H. S. Rho , S. P. Pujari , A. Lenferink , H. Zuilhof , L. W. M. M. Terstappen , C. Otto , S. L. Gac , Lab Chip 2019, 19, 2526. 31292600 10.1039/c9lc00081j

[369] Y. Yang , D. Arseni , W. Zhang , M. Huang , S. Lövestam , M. Schweighauser , A. Kotecha , A. G. Murzin , S. Y. Peak-Chew , J. Macdonald , I. Lavenir , H. J. Garringer , E. Gelpi , K. L. Newell , G. G. Kovacs , R. Vidal , B. Ghetti , B. Ryskeldi-Falcon , S. H. W. Scheres , M. Goedert , Science 2022, 375, 167. 35025654 10.1126/science.abm7285 PMC7612234

[370] C. O. S. Sorzano , J. L. Vilas , E. Ramírez-Aportela , J. Krieger , D. d. Hoyo , D. Herreros , E. Fernandez-Giménez , D. Marchán , J. R. Macías , I. Sánchez , L. d. Caño , Y. Fonseca-Reyna , P. Conesa , A. García-Mena , J. Burguet , J. G. Condado , J. M. García , M. Martínez , A. Muñoz-Barrutia , R. Marabini , J. Vargas , J. M. Carazo , Faraday Discuss. 2022, 240, 210. 35861059 10.1039/d2fd00059h

[371] C. Berger , N. Premaraj , R. B. G. Ravelli , K. Knoops , C. López-Iglesias , P. J. Peters , Nat. Methods 2023, 20, 499. 36914814 10.1038/s41592-023-01783-5

[372] C. C. Moura , A. Miranda , R. O. C. Oreffo , P. A. A. De Beule , Biochem. Biophys. Res. Commun. 2020, 529, 392. 32703441 10.1016/j.bbrc.2020.06.037

[373] K. Beton‐Mysur , B. Brożek‐Płuska , Anal. Methods 2023, 15, 5199. 37781815 10.1039/d3ay01040f

[374] A. Colom , E. Derivery , S. Soleimanpour , C. Tomba , M. D. Molin , N. Sakai , M. González-Gaitán , S. Matile , A. Roux , Nat. Chem. 2018, 10, 1118. 30150727 10.1038/s41557-018-0127-3 PMC6197433

[375] C. Roffay , J. M. García-Arcos , P. Chapuis , J. López-Andarias , F. Schneider , A. Colom , C. Tomba , I. Di Meglio , K. Barrett , V. Dunsing , S. Matile , A. Roux , V. Mercier , Nat. Protoc. 2024, 19, 3457. 39210094 10.1038/s41596-024-01027-6

[376] J. Jumper , R. Evans , A. Pritzel , T. Green , M. Figurnov , O. Ronneberger , K. Tunyasuvunakool , R. Bates , A. Žídek , A. Potapenko , A. Bridgland , C. Meyer , S. A. A. Kohl , A. J. Ballard , A. Cowie , B. Romera-Paredes , S. Nikolov , R. Jain , J. Adler , T. Back , S. Petersen , D. Reiman , E. Clancy , M. Zielinski , M. Steinegger , M. Pacholska , T. Berghammer , S. Bodenstein , D. Silver , O. Vinyals , A. W. Senior , K. Kavukcuoglu , P. Kohli , D. Hassabis , Nature 2021, 596, 583. 34265844 10.1038/s41586-021-03819-2 PMC8371605

[377] L. Puppulin , D. Kanayama , N. Terasaka , K. Sakai , N. Kodera , K. Umeda , A. Sumino , A. Marchesi , W. Weilin , H. Tanaka , T. Fukuma , H. Suga , K. Matsumoto , M. Shibata , ACS Appl. Mater. Interfaces 2021, 13, 54817. 34766499 10.1021/acsami.1c17708

[378] Y. Qiu , E. S. Sajidah , S. Kondo , S. Narimatsu , M. I. Sandira , Y. Higashiguchi , G. Nishide , A. Taoka , M. Hazawa , Y. Inaba , H. Inoue , A. Matsushima , Y. Okada , M. Nakada , T. Ando , K. Lim , R. W. Wong , Cells 2024, 13, 279. 38334671 10.3390/cells13030279 PMC10855070

[379] A. Magazzù , C. Marcuello , Nanomaterials 2023, 13, 963. 36985857 10.3390/nano13060963 PMC10053849

[380] J. Hu , S. Chen , D. Huang , Y. Zhang , S. Lü , M. Long , Biophys. Rep. 2020, 6, 9.

