# Supplementary material for: Nanoscale Analysis beyond Imaging by Atomic Force Microscopy: Molecular Perspectives on Oncology and Neurodegeneration
Source: Small Sci. 2025 Oct 12;5(11):2500351. doi: 10.1002/smsc.202500351 (PMC12622512; doi:10.1002/smsc.202500351)
Supplement: Supplementary file 1 — Supplementary Material [file SMSC-5-2500351-s001.pdf]

Nanoscale analysis beyond imaging by atomic force microscopy: molecular perspectives on oncology and neurodegeneration

Carlos Marcuello[1,2,*], KeeSiang Lim[3], Giacomo Nisini[4,5], Vadim S. Pokrovsky[6], João Conde[7], Francesco Simone Ruggeri[4,5,*]

[1]Laboratorio de Microscopias Avanzadas (LMA), Universidad de Zaragoza, Zaragoza 50018, Spain

[2]Biofisika Institute (CSIC, UPV/EHU), 48940 Leioa, Spain

[3]WPI-Nano Life Science Institute, Kanazawa University, Ishikawa 920-1192, Japan

[4]Wageningen University, Laboratory of Organic Chemistry, Stippeneng 4, 6708 WE, Wageningen, the Netherlands

[5]Wageningen University, Physical Chemistry and Soft Matter, Stippeneng 4, 6708 WE, Wageningen, the Netherlands

[6]Patrice Lumumba People's Friendship University, 117198 Moscow, Russia

[7]ToxOmics, NOVA Medical School (NMS), Faculdade de Ciências Médicas (FCM), Universidade Nova de Lisboa 1099-085 Lisboa, Portugal

cmarcuel@unizar.es, simone.ruggeri@wur.nl

Supplementary Tables

Table 1. Adhesion force values obtained for the studied amyloidogenic protein and tumour cell systems at a specific loading rate. The terms *F** and *r* refer to the most probable rupture force of a single formed complex and the loading rate of the AFM lever, respectively. HBME, HBCC, and PDL1 refer to the human bone marrow endothelium, human breast cancer cells, and programmed death ligand-1, respectively. N.D. corresponds to those "non-described" conditions.

Sample	F* (pN)	r (nN/s)	k_{off} (s^{-1})	REF
$A\beta_{42}$:$A\beta_{42}$-Hepes	~110	10.0	12.5 ± 9.6	[1]
$A\beta_{42}$:$A\beta_{42}$-Cu^{2+} (20 nM)	~170	10.0	23.4 ± 9.7	[1]
$A\beta_{42}$:$A\beta_{42}$-Zn^{2+} (20 nM)	~120	10.0	57.3 ± 56.3	[1]
$A\beta_{14-23}$: $A\beta_{14-23}$-PBS	185 ± 42	15.0	8.9 ± 2.2	[2]
$A\beta_{40}$:$A\beta_{40}$-PBS	63.4 ± 3.2	6.0	9.0 ± 2.4	[3]
[VPV]$A\beta_{40}$:[VPV]$A\beta_{40}$-PBS	79.3 ± 1.5	6.0	1.9 ± 0.1	[3]
$A\beta_{42}$:$A\beta_{42}$-PBS	66.2 ± 0.8	6.0	5.7 ± 0.3	[3]
[VPV]$A\beta_{42}$:[VPV]$A\beta_{42}$-PBS	67.9 ± 1.0	6.0	1.6 ± 0.3	[3]
$A\beta_{14-23}$ (tetramer) pH 7.0	45 ± 19	15.0	N.D.	[4]
$A\beta_{14-23}$ (tetramer) pH 3.7	74 ± 27	15.0	N.D.	[4]
Endothelial cell:RT112 bladder	20.8 ± 0.7	5.0	N.D.	[5]
Endothelial cell:J82 bladder	31.6 ± 1.0	5.0	N.D.	[5]
HBME:HBCC(MB231)-0.5 s	23.3 ± 5.7	48.0	N.D.	[5]
HBME:HBCC(MB231)-300 s	553.6 ± 86.1	~17.5	N.D.	[5]
MCF10A breast-Tris pH 7.5	141 ± 39	N.D.	N.D.	[6]
MCF10AT breast-Tris	139 ± 64	N.D.	N.D.	[6]
MCF10CA1a breast-Tris	129 ± 38	N.D.	N.D.	[6]
SKBR3:SKBR3 breast	370 ± 140	15.0	N.D.	[7]
MDA-MB231:MDA-MB231 breast	840 ± 190	15.0	N.D.	[7]
FP10SC2: FP10SC2 (highly) breast-0s	~400	300.0	N.D.	[7]
4T1-LM:4T1-LM (low) breast-0s	~300	300.0	N.D.	[7]
FP10SC2: FP10SC2 (highly) breast-60s	~3500	300.0	N.D.	[7]
4T1-LM:4T1-LM (low) breast-60s	~1500	300.0	N.D.	[7]
T cell:PDL1 on lung cancer-0 s	~250	300.0	N.D.	[8]
T cell:PDL1 (Nivolumab)-0s	~300	300.0	N.D.	[8]
T cell:PDL1 on lung cancer-60 s	~900	300.0	N.D.	[8]
T cell:PDL1 (Nivolumab)-60s	~1500	300.0	N.D.	[8]

Table 2. Classification of the existing elastic and viscoelastic models according to AFM tip geometry. E^* corresponds to the apparent Young's modulus of the indented sample, F is the load force, δ is the indentation depth, α is the half-angle of the AFM tip with a conical or pyramidal shape, R is radius of curvature of the probe tip, w is the work of adhesion. For the viscoelastic equations, E_0 is the instantaneous elastic modulus, t is the scaling time, υ is the Poisson's ratio of the sample, v is the AFM lever approach velocity, β is the power-law exponent, also known as fluidity, Γ is the gamma function, and ξ is the tip geometry coefficient.

Model	AFM tip geometry	Equation	[REF]
Elastic Hertz	Spherical ($\delta \ll R$) / Parabolic	$F_H(\delta) = \frac{4}{3} E^* R^{1/2} \delta^{3/2}$	[9,10]
Elastic Hertz	4-sided regular pyramid	$F_H(\delta) = \frac{tan(\alpha)}{\sqrt{2}} E^* \delta^2$	[10,11]
Elastic Sneddon	Conical	$F_{sn}(\delta) = \frac{2tan(\alpha)}{\pi} E^* \delta^2$	[12, 9]
Elastic DMT	Spherical	$F_{DMT}(\delta) = F_H(\delta) - 2\pi R w$	[13, 10,14]
Elastic JKR	Spherical	$F_{JKR}(\delta) = F_H(\delta) - \sqrt{6\pi w E^* R \delta} + 3\pi R w$	[15, 10,16]
Viscoelastic	Parabolic	$F(t) = \frac{v^{3/2}}{\xi} E_0 \frac{t_0^{\beta} 3\sqrt{\pi}\, \Gamma(1-\beta)}{4\Gamma\left(\frac{5}{2}-\beta\right)} t^{\frac{3}{2}-\beta}$ $\xi = \frac{3}{4} \frac{(1-\upsilon^2)}{\sqrt{R}}$	[17]
Viscoelastic	Conical/4-sided regular pyramid	$F(t) = 2\frac{v^2}{\xi} E_0 \frac{t_0^{\beta} \Gamma[2]\, \Gamma[1-\beta]}{\Gamma(3-\beta)} t^{2-\beta}$ $\xi = \frac{1.3\,(1-\upsilon^2)}{\tan\alpha}$ (4-sided reg. pyram.) $\xi = \frac{\pi(1-\upsilon^2)}{2\tan\alpha}$ (Conical)	[17]

Table 3. Poisson ratio (υ) values used to ascertain the Young's modulus of different biology systems and drug carriers. The examined samples were ordered by increasing Poisson's ratio and alphabetical order.

Sample	Poisson ratio (υ)	[REF]
Lung cancer cells	0.25	[18]
Brain endothelial cells: $A\beta_{42}$	0.30	[19]
Amyloid oligomers/fibrils	0.3	[20]
Biomolecular condensates	0.5	[21]
Cartilage tissue	0.30	[22]
DNA Origami	0.30	[23]
Lipid vesicles	0.30	[24]
Lung parenchyma tissue	0.34	[25]
Osteocytes and cancer cells	0.35	[26]
Gold NPs	0.40	[27]
Lung tissue	0.40	[28]
Breast cancer cells	0.50	[29]
Breast cancer cells	0.50	[30]
Breast cancer cells	0.50	[31]
Breast tissue (health)	0.50	[32]
Chondrocyte cells	0.50	[33]
Lung tissue	0.50	[34]

Table 4. Estimated elastic modulus of amyloidogenic proteins, tissues, and cells associated with cancer malignancies and some suitable drug deliveries presented in alphabetical order. "A" and "L" subscripts correspond to those nanoindentation measurements carried out in air and liquid conditions, respectively. The applied force and indentation depth parameters are defined as "Force" and "Ind.," respectively. N.D. refers to those "non-described" conditions.

Samp. nature	Sample	Force/Ind.	Elastic modulus	[Ref]
Amyl. protein	$A\beta_{40\ A}$	150 nN/N.D.	1.8 ± 0.4 GPa	[35]
Amyl. protein	$A\beta_{40\ L}$	150 nN/N.D.	19.7 ± 8.6 MPa	[35]
Amyl. protein	$A\beta_{42\ A}$	N.D./N.D.	3.2 ± 0.8 GPa	[19,36,37]
Amyl. protein	$A\beta_{42}$:human brain endothelial cell $_L$	50 pN/1 µm	649.2 ± 7.7 Pa	[19]
Amyl. protein	$A\beta_{42}$:hyppocampus neurons $_L$	2 nN/N.D.	302.4 ± 41.3 Pa	[38]
Amyl. protein	$A\beta_{42}$:human neuroblastoma (1 h)$_L$	3 nN/600 nm	1.8 ± 0.2 kPa	[39]
Amyl. protein	$A\beta_{42}$:human neuroblastoma (72 h)$_L$	3 nN/600 nm	2.0 ± 0.3 kPa	[39]
Amyl. protein	$A\beta_{42}$(1 µM):h. neuroblastoma $_L$	3 nN/600 nm	1.4 ± 0.2 kPa	[39]
Amyl. protein	$A\beta_{42}$(10 µM):h. neuroblastoma $_L$	3 nN/600 nm	0.7 ± 0.1 kPa	[39]
Amyl. protein	α-syn $_L$	2 nN/ <5 nm	1.6 ± 0.3 GPa	[40]
Amyl. protein	α-syn $_A$	2 nN/ <5 nm	2.1 ± 0.5 GPa	[36,37,40]
Amyl. protein	Exon1 Huntingtin 14Q $_A$	N.D./N.D.	1.5 ± 0.7 GPa	[41]
Amyl. protein	Exon1 Huntingtin 22Q Nt17-truncated $_A$	N.D./N.D.	1.72 ± 0.79 GPa	[41]
Amyl. protein	Exon1 Huntingtin 22Q $_A$	N.D./N.D.	2.03 ± 0.77 GPa	[41]
Amyl. protein	Exon1 Huntingtin 28Q Nt17-truncated $_A$	N.D./N.D.	1.9 ± 0.75 GPa	[41]
Amyl. protein	Exon1 Huntingtin 28Q $_A$	N.D./N.D.	2.3 ± 0.75 GPa	[41]
Amyl. protein	Exon1 Huntingtin 42Q Nt17-truncated $_A$	N.D./N.D.	2.7 ± 0.82 GPa	[41]
Amyl. protein	Exon1 Huntingtin 42Q $_A$	N.D./N.D.	2.8 ± 0.77 GPa	[41]
Amyl. protein	ILQINS $_A$	N.D./N.D.	2.5 ± 0.6 GPa	[42,43]
Amyl. protein	IFQINS $_A$	N.D./N.D.	2.5 ± 0.7 GPa	[42,43]
Amyl. protein	Insulin fibril $_A$	N.D./N.D.	3.2 ± 0.6 GPa	[36,43]
Amyl. protein	Insulin fibril (ϕ = 3 nm) $_L$	1 nN/2 nm	9.6 ± 1.0 MPa	[43]
Amyl. protein	Insulin fibril (ϕ = 6 nm) $_L$	1 nN/2 nm	12.6 ± 1.4 MPa	[43]
Amyl. protein	Josephin domain of ataxin-3 $_A$	N.D./N.D.	1.70 ± 0.65 GPa	[44,45]
Amyl. protein	Lysozyme fibrils $_A$	N.D./<1 nm	12.0 ± 2.0 GPa	[44]
Amyl. protein	Lysozyme fibrils (sonication)$_A$	N.D./<1 nm	11.9 ± 2.5 GPa	[44]
Amyl. protein	Peptide F8 $_A$	N.D./N.D.	0.79 ± 0.14 GPa	[46]
Amyl. protein	Peptide EF8E $_A$	N.D./N.D.	0.99 ± 0.18 GPa	[46]
Amyl. protein	Tau $_A$	N.D./N.D.	3.4 ± 0.7 GPa	[46 36]
Cellular	Brain (health) $_A$	5 nN/1 µm	1.2 ± 0.2 GPa	[47]
Cellular	Glioblastoma $_A$	5 nN/1 µm	380 ± 90 MPa	[47]
Tissular	Brain (health) $_L$	4 nN/1 µm	66.4 ± 39.2 Pa	[48]

Tissular	Glioblastoma $_L$	4 nN/1 µm	168.8 ± 32.2 Pa	[48]
Tissular	Meningothelial meningioma $_L$	4 nN/1 µm	52.7 ± 0.6 Pa	[48]
Tissular	Fibrous meningioma $_L$	4 nN/1 µm	69.8 ± 1.7 Pa	[48]
Tissular	Metastatic adenocarcinoma $_L$	4 nN/1 µm	242.2 ± 1.1 Pa	[48]
Tissular	Cortical bone (health) $_L$	0.5 nN/600 nm	~ 0.3 kPa	[49]
Tissular	Growth plate bone (health) $_L$	0.5 nN/600 nm	~ 91 Pa	[49]
Tissular	Bone marrow (health) $_L$	0.5 nN/600 nm	~ 6.7 Pa	[49]
Cellular	Bone (health) $_L$	0.2 nN/200 nm	0.7 ± 0.1 kPa	[50]
Cellular	Bone tumour $_L$	0.2 nN/300 nm	0.3 ± 0.1 kPa	[50]
Cellular	Adaptative bone tumour $_L$	0.2 nN/300 nm	0.2 ± 0.1 kPa	[50]
Cell.-MCF-10A	Benign breast epithelial $_L$	0.4 nN/4 nm	2.94 ± 1.35 kPa	[51]
Cell.-MCF-7	Malign breast cancer $_L$	0.4 nN/4 nm	1.94 ± 1.02 kPa	[51]
Cell.-MCF-10A	Benign breast epithelial drug resistant	0.3 nN/<5 nm	~ 2.2 kPa	[52]
Cell.-MCF-10A	Benign epithelial cancer $_L$	0.5nN/<50nm	5.3 ± 1.1 kPa	[6]
C.-MCF-10AT	Premalignant breast cancer $_L$	0.5nN /<50nm	3.7 ± 1.3 kPa	[6]
C-MCF10CA1a	Malign breast cancer $_L$	0.5nN /<50nm	10.7 ± 3.1 kPa	[6]
Cell.-MCF-10A	Benign breast epithelial $_L$	0.7nN/>50nm	37.5 ± 18.2 kPa	[6]
C.-MCF-10AT	Premalignant breast cancer $_L$	0.7nN />50nm	20.5 ± 5.0 kPa	[6]
C-MCF10CA1a	Malign breast cancer $_L$	0.7nN />50nm	15.9 ± 2.7 kPa	[6]
Tissular	Liver (health) $_L$	2-3 nN/2 µm	1.19 ± 0.58 kPa	[53]
Tissular	Paraneoplastic liver $_L$	2-3 nN/2 µm	1.10 ± 0.20 kPa	[53]
Tissular	Neoplastic liver $_L$	2-3 nN/2 µm	0.42 ± 0.17 kPa	[53]
Tissular	Liver cirrhosis $_L$	2-3 nN/2 µm	1.06 ± 0.49 kPa	[53]
Tissular	Hepatocarcinoma $_L$	2-3 nN/2 µm	0.58 ± 0.40 kPa	[53]
Cellular L02	Liver (health) $_L$	0.8 nN/0.5 µm	~ 1.0 kPa	[53]
C.-SMMC7721	Liver-low metastatic $_L$	0.8 nN/0.5 µm	~ 1.5 kPa	[53]
Cell.-HCCLM3	Liver–high metastatic $_L$	0.8 nN/0.5 µm	~ 0.6-0.7 kPa	[53]
Cellular Calu6	Lung $_L$	N.D./N.D.	~ 33 Pa	[54]
Cellular A549	Lung $_L$	N.D./N.D.	~ 1225 Pa	[54]
Cellular Calu6	Lung:cisplatin (48h)$_L$	N.D./N.D.	~ 1105 Pa	[54]
Cellular A549	Lung:cisplatin (48h)$_L$	N.D./N.D.	~ 2375 Pa	[54]
Cellular Calu6	Lung:cetuximab (48h)$_L$	N.D./N.D.	~ 309 Pa	[54]
Cellular A549	Lung:cetuximab (48h)$_L$	N.D./N.D.	~ 12609 Pa	[54]
Tissular	Skin (health) $_A$	2.9µN/0.5µm	401 ± 148 MPa	[55]
Tissular	Patient skin (benign nevus)$_A$	2.9µN/0.5µm	575 ± 107 MPa	[55]
Tissular	Patient skin (melanoma)$_A$	2.9µN/0.5µm	188-787 MPa	[55]

Cellular-IOSE	Ovarian (non-malignant) $_L$	15 nN/1 µm	2.47 ± 2.0 kPa	[56]
Cell.-OVCAR4	Ovarian cancer $_L$	5 nN/1 µm	1.12 ± 0.86 kPa	[56]
Cell.-HEY A8	Highly invasive ovarian cancer $_L$	5 nN/1 µm	0.49 ± 0.22 kPa	[56]
Cell.-SKOV-3	Ovarian cancer (health) $_L$	0.6 nN/N.D.	2.99 ± 0.18 kPa	[57]
Cell.-SKOV-3	Ovarian cancer:docetaxel $_L$	0.6 nN/N.D.	4.69 ± 0.23 kPa	[57]
C.-MDA PCa2b	Prostate (health) $_A$	10 µN/1 µm	24.3 ± 8.4 MPa	[58]
C.-MDA PCa2b	Prostate (health) $_A$	10µN/0.5µm	27.7 ± 19.3 MPa	[58]
Cellular	Prostate tumoroids $_A$	10 µN/1 µm	5.8 ± 2.1 MPa	[58]
Cellular	Prostate tumoroids $_A$	10µN/0.5µm	5.7 ± 0.7 MPa	[58]
Cellular	Prostate tumoroids (+20 days)$_A$	10 µN/1 µm	5.1 ± 1.5 MPa	[58]
Cellular	Prostate tumoroids (+20 days)$_A$	10 µN/0.5 µm	4.7 ± 0.7 MPa	[58]
Cellular	Red blood (health) $_L$	1-2.5nN/<200nm	7.57 ± 3.25 kPa	[59]
Cellular	Neutrophils (health) $_L$	800 pN/3 µm	156 ± 87 Pa	[60]
Cellular-Jurkat	Lymphoid $_L$	800 pN/3 µm	48 ± 35 Pa	[60]
Cellular-HL60	Myeloid $_L$	800 pN/3 µm	855 ± 670 Pa	[60]
Cellular-Raji	Lymphoblast $_L$	200 pN 0.5 µm	~ 0.2-0.4 kPa	[61]
Cellular-HuT	T-cell lymphoma $_L$	200 pN 0.5 µm	~ 1.0-1.4 kPa	[61]
Tissular	Thyroid papillary (P) (health)$_L$	0.4nN/0.3-1.5µm	0.54 ± 0.28 kPa	[32]
Tissular	Thyroid P cancer $_L$	0.4nN/0.3-1.5µm	1.70 ± 1.70 kPa	[32]
Tissular	Thyroid P-Anaplastic (health)$_L$	0.4nN/0.3-1.5µm	1.4 ± 1.6 kPa	[32]
Tissular	Thyroid P-Anaplastic cancer $_L$	0.4nN/0.3-1.5µm	0.07 ± 0.06 kPa	[32]
Drug carrier	DNA Origami (monolayer)$_L$	100 pN/4 nm	9.6 ± 1.9 MPa	[23]
Drug carrier	DNA Origami (bilayer)$_L$	100 pN/4 nm	18.1 ± 4.1 MPa	[23]
Drug carrier	DNA origami (monolayer)$_A$	900 pN/<5nm	535 ± 68 MPa	[62]
Drug carrier	G-5 Poly(amidoamine) dendrimer $_A$	6.0 nN/1 nm	~150 MPa	[63]
Drug carrier	Agg. G5-PAMAM dendrimer $_A$	6.0 nN/3 nm	~700 MPa	[63]
Drug carrier	Exosomes (50-170 nm) $_L$	190-500pN/4nm	~22.4 MPa	[64]
Drug carrier	Exosomes (<30nm) $_L$	190-500pN/4nm	6.6 ± 0.6 MPa	[64]
Drug carrier	Gold NPs $_A$	200 nN/3 nm	~140 GPa	[27]
Drug carrier	PEGylated gold NPs $_A$	30 nN/<5nm	2.6 ± 0.7 GPa	[65]
Drug carrier	Gold NPs conjugated streptavidin $_A$	5 µN/4 nm	1.3 ± 0.6 GPa	[66]
Drug carrier	Gold NPs conjugated BSA $_A$	5 µN/4 nm	9.5 ± 7.7 GPa	[66]
Drug carrier	Poly(lactic-co-glycolic) NPs $_L$	2.0 nN/<10nm	1.0 ± 2.0 MPa	[67]
Drug carrier	PLGA NPs $_A$	2.0 nN/4 nm	2.9 ± 0.7 GPa	[68]

REFERENCES

1 Hane, F. T., Hayes, R., Lee, B. Y. & Leonenko, Z. Effect of Copper and Zinc on the Single Molecule Self-Affinity of Alzheimer's Amyloid-β Peptides. *PLOS ONE* **11**, e0147488, (2016).

2 Maity, S. & Lyubchenko, Y. L. Probing of Amyloid Aβ (14–23) Trimers by Single-Molecule Force Spectroscopy. *Jacobs J Mol Transl Med* **1**, 004, (2016).

3 Lv, Z., Roychaudhuri, R., Condron, M. M., Teplow, D. B. & Lyubchenko, Y. L. Mechanism of amyloid β-protein dimerization determined using single-molecule AFM force spectroscopy. *Sci Rep-Uk* **3**, 2880, (2013).

4 Maity, S., Viazovkina, E., Gall, A. & Lyubchenko, Y. L. Single-molecule probing of amyloid nano-ensembles using the polymer nanoarray approach. *Phys. Chem. Chem. Phys.* **19**, 16387-16394, (2017).

5 Laurent, V. M., Duperray, A., Rajan, V. S. & Verdier, C. Atomic Force Microscopy Reveals a Role for Endothelial Cell ICAM-1 Expression in Bladder Cancer Cell Adherence. *PLOS ONE* **9**, e98034, (2014).

6 Dumitru, A. C. *et al.* Label-Free Imaging of Cholesterol Assemblies Reveals Hidden Nanomechanics of Breast Cancer Cells. *Advanced Science* **7**, 2002643, (2020).

7 Smolyakov, G. *et al.* Elasticity, Adhesion, and Tether Extrusion on Breast Cancer Cells Provide a Signature of Their Invasive Potential. *ACS Appl. Mater. Interfaces* **8**, 27426-27431, (2016).

8 Kim, H., Ishibashi, K., Iijima, M., Kuroda, S. i. & Nakamura, C. Influence of Nivolumab for Intercellular Adhesion Force between a T Cell and a Cancer Cell Evaluated by AFM Force Spectroscopy. *Sensors* **20**, 5723, (2020).

9 Kontomaris, S. V., Malamou, A. & Stylianou, A. The Hertzian theory in AFM nanoindentation experiments regarding biological samples: Overcoming limitations in data processing. *Micron* **155**, 103228, (2022).

10 Butt, H.-J., Cappella, B. & Kappl, M. Force measurements with the atomic force microscope: Technique, interpretation and applications. *Surface Science Reports* **59**, 1-152, (2005).

11 Rico, F. *et al.* Probing mechanical properties of living cells by atomic force microscopy with blunted pyramidal cantilever tips. *Phys Rev E Stat Nonlin Soft Matter Phys* **72**, 021914, (2005).

12 Sneddon, I. N. The relation between load and penetration in the axisymmetric boussinesq problem for a punch of arbitrary profile. *International Journal of Engineering Science* **3**, 47-57, (1965).

13 Derjaguin, B. V., Muller, V. M. & Toporov, Y. P. Effect of contact deformations on the adhesion of particles. *Journal of Colloid and Interface Science* **53**, 314-326, (1975).

14 Fujinami, S., Ueda, E., Nakajima, K. & Nishi, T. Analytical methods to derive the elastic modulus of soft and adhesive materials from atomic force microcopy force measurements. *Journal of Polymer Science Part B: Polymer Physics* **57**, 1279-1286, (2019).

15 Johnson, K. L., Kendall, K., Roberts, A. D. & Tabor, D. Surface energy and the contact of elastic solids. *Proceedings of the Royal Society of London. A. Mathematical and Physical Sciences* **324**, 301-313, (1997).

16 Efremov, Y. M., Bagrov, D. V., Kirpichnikov, M. P. & Shaitan, K. V. Application of the Johnson–Kendall–Roberts model in AFM-based mechanical measurements on cells and gel. *Colloids and Surfaces B: Biointerfaces* **134**, 131-139, (2015).

17 Brückner, B. R., Nöding, H. & Janshoff, A. Viscoelastic Properties of Confluent MDCK II Cells Obtained from Force Cycle Experiments. *Biophys J* **112**, 724-735, (2017).

18 Zitnay, R. G. *et al.* Mechanics of lung cancer: A finite element model shows strain amplification during early tumorigenesis. *PLOS Computational Biology* **18**, e1010153, (2022).

19 Kulkarni, T., Angom, R. S., Das, P., Bhattacharya, S. & Mukhopadhyay, D. Nanomechanical insights: Amyloid beta oligomer-induced senescent brain endothelial cells. *Biochimica et Biophysica Acta (BBA) - Biomembranes* **1861**, 183061, (2019).

20 Adamcik, J. & Mezzenga, R. Study of amyloid fibrils via atomic force microscopy. *Curr Opin Colloid In* **17**, 369-376, (2012).

21 Lau, H. K. *et al.* Microstructured Elastomer-PEG Hydrogels via Kinetic Capture of Aqueous Liquid–Liquid Phase Separation. *Advanced Science* **5**, 1701010, (2018).

22 Jin, H. & Lewis, J. L. Determination of Poisson's ratio of articular cartilage by indentation using different-sized indenters. *J Biomech Eng* **126**, 138-145, (2004).

23 Li, L. *et al.* Measurement of nanomechanical properties of DNA molecules by PeakForce atomic force microscopy based on DNA origami. *Nanoscale* **11**, 4707-4711, (2019).

24 Tang, Y., Chen, X., Yoo, J., Yethiraj, A. & Cui, Q. Numerical Simulation of Nanoindentation and Patch Clamp Experiments on Mechanosensitive Channels of Large Conductance in Escherichia coli. *Exp Mech* **49**, 35-46, (2009).

25 Birzle, A. M., Martin, C., Uhlig, S. & Wall, W. A. A coupled approach for identification of nonlinear and compressible material models for soft tissue based on different experimental setups - Exemplified

and detailed for lung parenchyma. *Journal of the Mechanical Behavior of Biomedical Materials* **94**, 126-143, (2019).
26 Ti, F., Chen, X., Yang, H., Liu, S. & Lu, T. J. A theory of mechanobiological sensation: strain amplification/attenuation of coated liquid inclusion with surface tension. *Acta Mech. Sin.* **37**, 145-155, (2021).
27 Li, H., Han, Y., Duan, T. & Leifer, K. Size-dependent elasticity of gold nanoparticle measured by atomic force microscope based nanoindentation. *Appl Phys Lett* **115**, 053104, (2019).
28 Sicard, D. *et al.* Aging and anatomical variations in lung tissue stiffness. *Am J Physiol Lung Cell Mol Physiol* **314**, L946-L955, (2018).
29 Acerbi, I. *et al.* Human breast cancer invasion and aggression correlates with ECM stiffening and immune cell infiltration. *Integr Biol (Camb)* **7**, 1120-1134, (2015).
30 Islam, M. T. *et al.* Non-invasive imaging of Young's modulus and Poisson's ratio in cancers in vivo. *Sci Rep-Uk* **10**, 7266, (2020).
31 Kim, H., Ishibashi, K., Okada, T. & Nakamura, C. Mechanical Property Changes in Breast Cancer Cells Induced by Stimulation with Macrophage Secretions in Vitro. *Micromachines* **10**, 738, (2019).
32 Levillain, A. *et al.* Mechanical properties of breast, kidney, and thyroid tumours measured by AFM: Relationship with tissue structure. *Materialia* **25**, 101555, (2022).
33 Ofek, G., Wiltz, D. C. & Athanasiou, K. A. Contribution of the Cytoskeleton to the Compressive Properties and Recovery Behavior of Single Cells. *Biophys J* **97**, 1873-1882, (2009).
34 Jorba, I. *et al.* Nonlinear elasticity of the lung extracellular microenvironment is regulated by macroscale tissue strain. *Acta Biomaterialia* **92**, 265-276, (2019).
35 Paul, T. J. *et al.* Structural and Mechanical Properties of Amyloid Beta Fibrils: A Combined Experimental and Theoretical Approach. *J. Phys. Chem. Lett.* **7**, 2758-2764, (2016).
36 Adamcik, J. *et al.* Measurement of intrinsic properties of amyloid fibrils by the peak force QNM method. *Nanoscale* **4**, 4426-4429, (2012).
37 Ruggeri, F. S. *et al.* Influence of the beta-Sheet Content on the Mechanical Properties of Aggregates during Amyloid Fibrillization. *Angew Chem Int Edit* **54**, 2462-2466, (2015).
38 Li, D. *et al.* Nanomechanical Profiling of Aβ42 Oligomer-Induced Biological Changes in Single Hippocampus Neurons. *Acs Nano* **17**, 5517-5527, (2023).
39 Gao, Q. *et al.* Dynamic effect of beta-amyloid 42 on cell mechanics. *Journal of Biomechanics* **86**, 79-88, (2019).
40 Sweers, K., van der Werf, K., Bennink, M. & Subramaniam, V. Nanomechanical properties of α-synuclein amyloid fibrils: a comparative study by nanoindentation, harmonic force microscopy, and Peakforce QNM. *Nanoscale Res Lett* **6**, (2011).
41 Ruggeri, F. S. *et al.* Nanoscale studies link amyloid maturity with polyglutamine diseases onset. *Sci Rep-Uk* **6**, (2016).
42 Adamcik, J. *et al.* Evolution of Conformation, Nanomechanics, and Infrared Nanospectroscopy of Single Amyloid Fibrils Converting into Microcrystals. *Advanced Science* **8**, (2021).
43 Guo, S. & Akhremitchev, B. B. Packing Density and Structural Heterogeneity of Insulin Amyloid Fibrils Measured by AFM Nanoindentation. *Biomacromolecules* **7**, 1630-1636, (2006).
44 Kozell, A. *et al.* Modulating amyloids' formation path with sound energy. *Proceedings of the National Academy of Sciences* **120**, e2212849120, (2023).
45 Ruggeri, F. S. *et al.* Infrared nanospectroscopy characterization of oligomeric and fibrillar aggregates during amyloid formation. *Nature Communications* **6**, (2015).
46 Wychowaniec, J. K., Moffat, J. & Saiani, A. Quantitative nanomechanical properties evaluation of a family of β-sheet peptide fibres using rapid bimodal AFM. *Journal of the Mechanical Behavior of Biomedical Materials* **124**, 104776, (2021).
47 Ciasca, G. *et al.* Nano-mechanical signature of brain tumours. *Nanoscale* **8**, 19629-19643, (2016).
48 Cieśluk, M. *et al.* Nanomechanics and Histopathology as Diagnostic Tools to Characterize Freshly Removed Human Brain Tumors. *International Journal of Nanomedicine* **15**, 7509-7521, (2020).
49 Chen, X. *et al.* Mechanical Heterogeneity in the Bone Microenvironment as Characterized by Atomic Force Microscopy. *Biophys J* **119**, 502-513, (2020).
50 Lee, T. Mechanical and Mechanosensing Properties of Tumor Affected Bone Cells Were Inhibited via PI3K/Akt Pathway. *J Bone Metab* **26**, 179-191, (2019).
51 Nguyen, N., Shao, Y., Wineman, A., Fu, J. & Waas, A. Atomic force microscopy indentation and inverse analysis for non-linear viscoelastic identification of breast cancer cells. *Mathematical Biosciences* **277**, 77-88, (2016).
52 Park, S. Mechanical Alteration Associated With Chemotherapeutic Resistance of Breast Cancer Cells. *Journal of Cancer Prevention* **23**, 87-92, (2018).

53 Tian, M. *et al.* The nanomechanical signature of liver cancer tissues and its molecular origin. *Nanoscale* **7**, 12998-13010, (2015).
54 Rezaei, I. & Sadeghi, A. The effects of cetuximab and cisplatin anti-cancer drugs on the mechanical properties of the lung cancerous cells using atomic force microscope. *Biochem. Cell Biol.* **101**, 531-537, (2023).
55 Jeon, B. *et al.* Melanoma Detection by AFM Indentation of Histological Specimens. *Diagnostics* **12**, 1736, (2022).
56 Xu, W. *et al.* Cell Stiffness Is a Biomarker of the Metastatic Potential of Ovarian Cancer Cells. *PLOS ONE* **7**, e46609, (2012).
57 Hou, Y., Zhao, C., Xu, B., Huang, Y. & Liu, C. Effect of docetaxel on mechanical properties of ovarian cancer cells. *Experimental Cell Research* **408**, 112853, (2021).
58 Molla, M. D. S., Katti, D. R. & Katti, K. S. Mechanobiological evaluation of prostate cancer metastasis to bone using an in vitro prostate cancer testbed. *Journal of Biomechanics* **114**, 110142, (2021).
59 Barns, S. *et al.* Investigation of red blood cell mechanical properties using AFM indentation and coarse-grained particle method. *BioMedical Engineering OnLine* **16**, 140, (2017).
60 Rosenbluth, M. J., Lam, W. A. & Fletcher, D. A. Force microscopy of nonadherent cells: a comparison of leukemia cell deformability. *Biophys J* **90**, 2994-3003, (2006).
61 Li, M. *et al.* Atomic force microscopy imaging and mechanical properties measurement of red blood cells and aggressive cancer cells. *Sci. China Life Sci.* **55**, 968-973, (2012).
62 Postigo, A. *et al.* Folding and Functionalizing DNA Origami: A Versatile Approach Using a Reactive Polyamine. *Journal of the American Chemical Society* **147**, 3919-3924, (2025).
63 Tomczak, N. & Vancso, G. J. Elasticity of Single Poly(amido amine) Dendrimers. *Macromol Rapid Comm* **28**, 1640-1644, (2007).
64 Bairamukov, V. Y. *et al.* Nanomechanical characterization of exosomes and concomitant nanoparticles from blood plasma by PeakForce AFM in liquid. *Biochimica et Biophysica Acta (BBA) - General Subjects* **1866**, 130139, (2022).
65 Lemoine, P., Dooley, C., Morelli, A., Harrison, E. & Dixon, D. AFM study of organic ligand packing on gold for nanoparticle drug delivery applications. *Applied Surface Science* **574**, 151386, (2022).
66 Wampler, H. P. & Ivanisevic, A. Nanoindentation of gold nanoparticles functionalized with proteins. *Micron* **40**, 444-448, (2009).
67 Dols-Perez, A. *et al.* Effect of surface functionalization and loading on the mechanical properties of soft polymeric nanoparticles prepared by nano-emulsion templating. *Colloids and Surfaces B: Biointerfaces* **222**, 113019, (2023).
68 Alsharif, N., Eshaghi, B., Reinhard, B. M. & Brown, K. A. Physiologically Relevant Mechanics of Biodegradable Polyester Nanoparticles. *Nano Lett.* **20**, 7536-7542, (2020).
